# Supplementary material for: Limbic Expression of mRNA Coding for Chemoreceptors in Human Brain—Lessons from Brain Atlases
Source: Int J Mol Sci. 2021 Jun 25;22(13):6858. doi: 10.3390/ijms22136858 (PMC8267617; doi:10.3390/ijms22136858)
Supplement: Supplementary file 1 [file ijms-22-06858-s001.zip › caption of supplemantaty.pdf]

Figure S1: **Expression of chemoreceptor families in other databases.** The global brain expression (fluorescence intensity, gcRMA normalization) of class I and II olfactory receptors, type 1 and 2 taste receptors, sour chemosensors, vomeronasal receptors, formyl peptide receptors, and trace amine-associated receptors (black boxes) were compared to the reported expression in the tongue (grey boxes). Dots represent individual values.

Figure S2: **Expression of outliers in the class II olfactory receptors and type 2 taste receptor families.** *OR2L13* mean expression – the highest one in all cerebral areas studied – is exceeded by *OR10A2* in the amygdala (top). Similarly, *TAS2R14* mean expression is exceeded by *TAS2R31* in the basal forebrain and the hypothalamus of a 31-year-old Caucasian male (bottom).

Figure S3: **Expression of outliers in the Gαs and Gαq family genes.** *Gαs* mean expression – the highest one in all cerebral areas studied – is exceeded by *Gαolf* in the amygdala, basal ganglia, and thalamus of a 31-year-old Caucasian male (top). Similarly, *Gαq* mean expression is exceeded by *Gα11* in the amygdala, basal forebrain, basal ganglia, and hypothalamus of the same subject (bottom).
