# Supplementary material for: Limbic Expression of mRNA Coding for Chemoreceptors in Human Brain—Lessons from Brain Atlases
Source: Int J Mol Sci. 2021 Jun 25;22(13):6858. doi: 10.3390/ijms22136858 (PMC8267617; doi:10.3390/ijms22136858)
Supplement: Supplementary file 1 [file ijms-22-06858-s001.zip › table S1.pdf]

OR Expression (log2)		AMYGDALA																							
		basolateral nucleus						basomedial nucleus						central nucleus						lateral nucleus					
Probe-name	Gene-symbol	24 years	31 years	39 years	49 years	55 years	57 years	24 years	31 years	39 years	49 years	55 years	57 years	24 years	31 years	39 years	49 years	55 years	57 years	24 years	31 years	39 years	49 years	55 years	57 years
**A_24_P409230**	**OR10A2**	**3.3069**	**2.4955**	**2.9007**	**1.5535**	**5.0789**	**2.5170**	**3.0794**	**4.3968**	**3.7809**	**3.3977**	**4.0438**	**2.0578**	**2.6464**	**3.4103**	**3.3104**	**3.3090**	**3.9974**	**1.9073**	**3.0318**	**2.1900**	**4.1681**	**1.6098**	**4.6697**	**1.8263**
**CUST_5622_PI416261804**	**OR10A2**	**5.0264**	**4.8543**	**4.9542**	**4.0720**	**5.8943**	**4.5243**	**5.0544**	**5.4380**	**5.5037**	**4.9433**	**4.9041**	**3.5788**	**4.0906**	**4.9643**	**5.0739**	**4.0043**	**4.3001**	**3.8022**	**4.7482**	**4.8532**	**5.2106**	**4.6582**	**5.4322**	**4.0972**
CUST_257_PI416573500	OR10A3	1.9538	2.0697	1.6605	1.1849	1.0049	1.8891	2.1596	3.4173	1.9144	0.7432	1.4329	1.7352	1.7300	1.9108	1.4423	1.3608	0.8005	2.1745	1.8191	2.7749	2.5147	1.2280	1.6761	3.4874
CUST_265_PI416379584	OR10A3	2.4745	2.3140	1.9774	1.0168	1.7948	1.8006	2.5386	3.1114	1.8306	3.3289	1.5999	1.6925	1.7717	1.8331	1.4988	1.2515	1.0148	2.5091	1.9777	3.0479	2.0774	1.0198	1.2603	3.7807
A_23_P75867	OR10A4	1.7871	2.1960	2.3284	1.2930	2.7208	2.0076	1.8333	1.7527	2.1999	1.0482	1.9380	1.9806	1.7140	2.0244	2.1192	1.3078	1.4678	1.7719	1.7296	2.1002	1.5140	1.3297	3.0150	1.5986
CUST_1625_PI416261804	OR10A4	3.9833	4.3215	4.2031	3.0999	4.7991	2.7182	3.6604	4.5419	4.5306	4.6296	3.4615	2.9578	2.7539	3.2446	3.4681	3.3669	3.9102	2.5990	3.3603	3.8609	4.2195	3.5389	5.3606	3.9571
A_23_P315991	OR10A5	5.7241	5.7020	5.7528	5.0732	6.5916	4.5980	5.9704	5.0741	5.8601	6.1881	6.0454	1.7680	3.2613	4.8323	4.6663	3.5945	5.2396	2.1455	6.2123	4.9353	5.1452	5.0982	7.8475	3.8681
CUST_1211_PI416261804	OR10A5	4.4203	2.1254	4.1241	1.8768	5.1368	1.7103	3.8773	5.1700	4.0683	4.1705	4.0550	1.5444	3.5955	3.9298	3.6347	1.8136	3.7490	1.5967	3.8058	2.5935	4.7528	2.4142	5.1313	2.2178
CUST_221_PI416573500	OR10A6	2.1549	2.0940	1.7216	1.1484	0.9650	1.7965	2.2617	1.7032	1.6698	2.1108	1.5688	1.7327	1.7677	1.8611	1.6602	1.2280	0.9894	1.5905	1.8728	1.9531	2.2658	1.1627	1.2425	1.7548
CUST_227_PI416379584	OR10A6	2.0886	2.2037	2.9634	1.6366	1.8258	2.4413	2.1594	1.7651	2.6843	1.4116	1.6552	2.0205	1.9051	2.2045	2.7280	1.6046	2.1108	1.8579	1.9557	1.9796	1.7852	1.8158	3.0996	1.7195
CUST_304_PI416573500	OR10A7	1.7621	1.9975	1.6539	1.1831	1.0695	1.8054	1.9527	1.5857	1.5117	0.7432	1.4101	1.7573	1.7033	1.8728	1.6994	1.3302	0.9004	1.5420	1.6931	1.8549	1.9184	1.1908	2.6190	1.4944
CUST_312_PI416379584	OR10A7	1.9782	2.0743	1.8713	0.9163	0.8064	1.5099	2.3828	2.4300	2.1811	1.0889	1.6286	2.3191	1.7039	2.8590	1.4586	1.1193	0.8599	1.4656	1.7789	1.7182	3.7529	1.2358	0.9364	1.8360
CUST_203_PI416573500	OR10AD1	1.7651	2.0795	1.6190	1.1350	1.0679	2.3668	1.9505	1.6475	1.6652	1.7765	1.4548	1.8182	1.6955	1.9347	1.6186	1.3105	0.9442	1.5756	1.6659	1.9157	1.9043	1.1326	1.3783	1.5160
CUST_208_PI416379584	OR10AD1	1.9232	4.1527	2.4348	1.4609	2.5453	1.9991	2.0648	2.3926	2.2936	0.8985	1.7027	1.9108	1.7649	1.9861	1.6954	1.2765	0.8299	1.9912	1.8238	2.2516	2.3982	1.3337	1.8575	1.9690
CUST_288_PI416573500	OR10AG1	2.7900	2.8696	3.3038	1.6083	1.5717	2.1028	2.8969	2.3310	3.3258	1.6723	1.5389	1.8813	2.2767	2.6270	3.3399	1.5371	0.9982	1.8595	2.3920	1.9803	1.6149	1.4752	2.1015	2.9170
CUST_296_PI416379584	OR10AG1	2.8351	3.6881	1.8432	1.5911	1.1302	2.1070	1.9258	2.4936	1.5485	0.7432	1.6078	2.5778	1.6457	3.7281	1.5635	1.5012	0.9488	1.5674	1.6657	2.5084	2.6132	1.3811	1.5901	1.5604
A_24_P60680	OR10C1	2.9738	2.0476	4.0548	3.1135	4.5090	1.9246	3.5584	3.6678	4.3158	3.3982	3.5008	1.7378	2.4507	2.4440	4.1375	1.1019	3.7736	2.4065	3.6529	1.8487	3.7151	3.2910	3.9083	2.3801
CUST_1194_PI416261804	OR10C1	7.8221	6.6750	8.0067	7.1350	8.1748	7.1009	7.2742	6.7580	8.1611	7.4630	6.7702	7.0841	6.1223	7.2772	8.6037	6.1133	6.7450	6.7958	7.5103	6.7499	7.8158	7.8031	7.3892	6.8651
CUST_398_PI416573500	OR10G2	2.5036	2.2987	3.4773	2.7982	4.4559	2.4314	2.7449	3.4020	3.8467	3.8079	3.0175	2.0657	3.0197	2.2225	3.7804	1.6117	3.5408	2.0111	3.6072	1.9891	3.6259	1.8586	4.2543	1.9514

CUST_407_PI4 16379584	OR10G2	5.0795	3.4256	6.1022	4.5224	5.9609	4.9950	4.7013	4.7482	5.6896	4.9199	4.5980	5.2314	2.2150	4.4228	6.2893	0.9717	4.8009	4.0777	4.6334	3.8273	5.7982	5.0139	5.0678	4.0246
CUST_332_PI4 16573500	OR10G3	3.4455	2.1740	3.2113	1.6816	2.4513	1.7583	3.5846	1.4801	3.0219	1.3763	1.8403	1.7107	2.9383	2.9640	1.7814	1.2580	1.4821	1.9191	3.2606	3.7129	3.8941	3.2984	3.1207	3.4890
CUST_341_PI4 16379584	OR10G3	1.6770	1.9353	2.2917	1.1697	2.7461	1.8040	1.7072	1.5694	2.2271	0.7432	1.3709	1.6859	1.6020	1.8492	1.6744	1.1446	1.4609	1.4820	1.5790	1.7488	1.8299	1.1748	1.0385	1.4584
CUST_313_PI4 16573500	OR10G4	1.5594	1.7083	1.9236	0.8272	1.6267	1.4243	1.8371	1.9113	1.9859	0.7432	1.1563	1.4918	1.6835	1.5479	1.6513	1.0804	0.9341	1.4197	1.6097	1.5862	2.2431	0.7432	1.3066	1.4776
CUST_321_PI4 16379584	OR10G4	1.6346	2.2212	2.1140	2.1122	1.0587	1.8857	1.8023	2.9005	2.4591	0.7432	1.5069	1.7205	1.5603	3.2633	1.4690	1.4218	0.8956	2.2886	1.5699	3.3293	2.5531	2.7579	0.9342	2.1448
CUST_154_PI4 16573500	OR10G7	1.8864	1.8775	1.6530	0.9210	0.8656	1.4818	2.2563	2.5068	2.1956	1.3296	1.7937	1.6396	1.8458	1.7789	1.7495	1.2640	3.2801	1.5460	1.7398	1.7327	2.4475	0.9026	0.9297	1.7204
CUST_159_PI4 16379584	OR10G7	2.0848	2.0522	1.6235	1.0975	1.2283	1.7228	2.3837	1.6965	1.7473	2.0124	2.3981	1.6106	1.7870	1.7974	1.5058	1.2687	1.0047	1.6063	1.8693	1.8522	2.2037	1.0600	1.2731	1.7824
**A_23_P125204**	**OR10G8**	**4.2808**	**3.4388**	**4.0032**	**0.9461**	**4.9411**	**2.2582**	**3.7294**	**4.5429**	**4.7232**	**2.7788**	**2.8574**	**1.4692**	**2.4629**	**2.5428**	**3.5313**	**1.2165**	**3.1590**	**1.8665**	**4.0293**	**3.2862**	**3.9872**	**1.9965**	**5.0881**	**2.6395**
**CUST_1990_PI 416261804**	**OR10G8**	**1.9360**	**2.1521**	**2.8082**	**1.2407**	**1.3789**	**1.9178**	**2.1703**	**2.7338**	**2.6825**	**0.7432**	**1.5802**	**1.8968**	**1.8674**	**1.9781**	**1.8881**	**2.1486**	**1.2759**	**1.7660**	**1.8659**	**2.1171**	**2.6408**	**1.2440**	**2.1142**	**1.7639**
CUST_385_PI4 16573500	OR10G9	1.8593	1.9182	1.6407	1.1367	1.1225	1.7185	2.0827	1.5036	1.5784	1.2478	1.4517	1.7896	1.8484	1.8967	1.7411	1.4014	2.0215	1.9303	1.7711	1.8046	1.5608	1.1229	1.6820	1.5655
CUST_394_PI4 16379584	OR10G9	2.1831	2.5196	2.4206	1.4919	0.7777	3.2460	2.2269	1.6837	2.4115	0.7432	2.9293	2.5223	1.6727	2.1702	1.4549	1.0359	0.7279	2.3267	2.6086	2.6381	3.1755	1.9227	1.3731	3.8868
A_23_P27917	OR10H1	2.1870	1.8041	3.1952	1.0245	4.0086	1.7057	2.5833	1.4467	2.6312	2.5861	3.5599	1.6532	2.0237	1.6965	2.3211	1.0483	1.7650	1.6763	2.0492	1.5585	3.1693	2.2069	2.8514	1.8684
CUST_843_PI4 16261804	OR10H1	1.6549	1.9711	1.8064	0.9859	2.1243	1.5883	1.9065	2.1597	2.3641	0.7432	1.4249	2.0438	1.6258	2.2295	1.6784	1.2222	0.9586	1.6962	1.5450	1.7806	2.8536	0.9621	3.4873	1.4754
A_23_P50799	OR10H2	9.4788	7.7976	9.4089	8.4316	8.8851	7.6371	9.6101	8.1932	9.4921	9.1334	8.0877	7.9489	7.1886	8.1610	9.8696	7.3120	8.0378	7.3965	8.9893	7.5842	8.9216	8.7097	8.4882	7.3709
CUST_836_PI4 16261804	OR10H2	6.0303	6.4340	7.6023	6.9921	7.0544	4.8936	5.0596	6.9958	7.6568	7.6415	5.9536	6.3860	4.9102	6.5231	7.8714	5.1844	6.2858	6.0201	6.0275	5.7183	7.4492	7.1235	6.6405	5.3354
A_23_P39410	OR10H3	1.7762	2.4846	2.1303	1.5008	3.0460	2.1336	1.7923	1.8534	4.0357	0.7432	2.9958	2.1073	1.7237	2.1893	1.9813	1.5281	1.3661	1.7259	1.6521	2.2287	2.5709	1.4473	2.4293	1.5582
CUST_1652_PI 416261804	OR10H3	2.8060	3.5887	1.5159	1.1108	2.5392	1.7818	2.5238	2.4139	2.1721	2.2956	1.9953	1.9047	1.8386	1.8504	1.5817	0.9997	2.1558	2.0933	2.2147	2.8162	3.0815	2.1739	2.0943	2.1662
A_23_P5258	OR10H4	1.9848	2.0587	2.4595	1.4395	3.5166	1.7610	2.3598	1.6721	2.5333	2.7237	2.6408	1.7277	2.2565	1.9018	1.7527	1.2707	2.0340	1.5290	1.8322	1.8919	2.5498	1.1573	2.3811	1.6634
CUST_758_PI4 16261804	OR10H5	4.1665	4.8164	5.7782	5.2531	5.5942	4.2804	5.7060	4.6465	5.7957	5.4565	4.8070	5.0209	4.6399	4.9383	6.0661	4.5908	5.2780	4.6893	5.2187	4.2755	5.1677	5.6210	5.3071	5.1642
A_23_P397468	OR10J1	1.8373	2.2710	2.0799	1.2097	1.9947	1.9599	1.9520	1.8108	1.8501	1.2872	1.7364	1.9923	1.7573	2.0302	1.8195	1.3516	2.9416	1.7823	1.7720	2.2127	1.5406	1.2146	2.0799	1.6780
CUST_10854_P I416261804	OR10J1	1.7962	2.3242	1.7483	1.2650	1.3158	2.0802	1.8545	1.8325	1.6773	0.8755	1.7069	2.0495	1.7608	2.1133	2.0232	1.3347	1.2133	1.8624	1.7503	2.2610	1.5992	1.2941	1.4239	1.6698
A_32_P385645	OR10J3	1.6520	2.0502	2.7286	1.5730	2.7644	2.0089	1.4934	2.0480	2.7449	3.0151	2.0795	1.8488	1.8313	1.9916	2.8768	1.2688	2.1487	1.6192	1.6945	1.8615	1.4709	1.4173	2.0783	2.4176
CUST_15805_P I416261804	OR10J3	2.0465	2.2468	1.6334	4.2040	0.9254	2.0405	3.0224	1.8277	1.5760	0.7432	1.6630	1.7964	1.7581	1.9622	1.6071	1.3112	0.8878	1.8721	1.8402	1.9769	1.5006	1.2983	1.2709	1.7131

A_23_P126031	OR10J5	2.2030	2.0476	2.0497	1.1981	2.2249	1.8782	2.0270	1.5817	2.1226	1.3490	1.7132	1.8495	2.0940	1.9124	1.8069	1.2003	0.8589	1.6491	1.7564	1.9124	1.9815	1.2298	3.3094	1.6550
CUST_11004_PI416261804	OR10J5	2.5279	2.2368	2.3763	1.2028	2.1681	1.8288	2.9865	1.8638	2.5128	0.7432	1.6828	2.7336	1.9626	1.9787	1.5383	1.7080	1.2381	2.2248	2.9481	3.1404	3.0660	2.7023	1.1145	2.6692
CUST_228_PI416573500	OR10K1	2.3463	2.1069	3.9137	3.4963	3.8986	2.2601	2.6058	2.2209	3.8318	3.6706	3.5011	1.9371	2.4488	2.0881	4.0995	2.3990	2.8595	1.8900	2.3465	2.4380	3.5258	3.5568	4.4931	1.8189
CUST_235_PI416379584	OR10K1	2.1395	1.9782	1.4530	0.9903	0.8645	2.7724	3.1613	2.8469	1.4286	0.7432	1.2048	1.4973	1.7650	3.5116	1.4182	1.1480	0.7166	1.6898	2.2644	1.7368	1.4480	2.0916	0.9202	2.6464
CUST_229_PI416573500	OR10K2	1.9569	2.2927	6.1027	1.3967	0.9622	2.1041	2.0229	1.6817	1.6909	0.7432	1.7183	1.9624	1.8316	2.0594	1.8046	1.3766	1.0262	1.7512	1.8139	2.0062	1.6488	1.4139	1.3835	1.7064
CUST_236_PI416379584	OR10K2	1.6962	2.0713	1.5189	2.0834	0.9465	4.5374	1.9480	2.0296	1.4394	0.7432	1.3228	1.7643	2.0610	1.9048	1.4720	1.2146	1.6995	2.0726	1.6428	1.8588	1.4196	1.2429	1.0212	1.4956
A_24_P167377	OR10P1	3.2244	3.1947	4.1272	3.7928	5.0960	3.0766	3.6904	4.0917	4.4263	3.6728	3.2904	3.3705	2.8435	4.2097	3.6861	4.2567	4.2257	2.0541	3.3354	2.0717	3.8659	3.9304	4.7904	2.5685
CUST_1722_PI416261804	OR10P1	1.6186	2.0051	1.9694	1.1369	2.6632	1.7170	1.6548	1.5981	2.4215	0.7432	1.5329	1.8527	1.7079	1.9908	1.9126	1.3179	1.2584	1.6521	1.5717	1.8423	2.2370	1.1411	2.5334	1.4551
CUST_318_PI416573500	OR10Q1	5.5381	1.6170	4.6782	3.4601	4.1861	3.2906	5.3983	1.1493	4.2830	3.2982	2.2840	3.9417	3.0413	1.5872	4.1668	4.1121	3.7836	2.8602	5.0215	1.9920	4.5587	1.0434	3.3679	3.7649
CUST_327_PI416379584	OR10Q1	1.9217	2.2194	1.6783	1.2750	1.0612	1.9282	2.0668	1.7323	1.6236	0.7432	1.7014	1.8344	2.3648	1.9812	1.7186	1.3263	1.1399	1.6398	1.7669	2.0392	2.1774	1.2737	1.3124	1.6681
A_23_P705	OR10R2	1.7732	2.2395	1.6335	1.2659	1.2034	2.0541	1.8168	1.8165	1.5654	0.8656	1.6701	1.9265	1.6651	2.0075	1.7306	1.2909	1.0084	1.7029	1.6996	2.1110	1.4474	1.3221	1.2820	1.5762
CUST_2013_PI416261804	OR10R2	1.8318	2.2271	1.8465	1.3102	2.8341	2.0754	1.8861	1.7740	2.4093	0.8787	1.6685	1.9978	1.7316	2.0417	1.8177	1.3003	1.2676	1.8102	1.7605	2.1238	1.5264	1.3784	3.5060	1.6442
CUST_319_PI416573500	OR10S1	2.1663	2.0666	2.3769	1.7517	2.7872	2.1697	2.6018	1.7998	2.2286	1.3058	1.6949	3.3325	1.9295	4.5967	1.7096	1.5321	1.4728	1.6143	4.4831	1.9071	2.9675	0.9701	3.4613	2.9394
CUST_328_PI416379584	OR10S1	1.8539	2.0956	1.6795	1.2814	0.9188	1.9085	1.9963	1.6761	2.0554	0.7432	1.6014	1.7865	1.7512	1.9604	1.7305	1.2783	1.0477	1.6178	1.7252	1.9317	1.5319	1.2795	1.2796	1.5982
A_23_P23507	OR10T2	1.6989	2.0167	2.4029	1.1436	1.2428	1.8555	1.7429	1.6369	1.5207	0.9113	1.4066	1.7033	1.6020	1.8282	1.6314	3.6672	0.7960	1.5363	1.6310	1.8597	1.4275	1.2071	1.0857	1.4983
CUST_2268_PI416261804	OR10T2	2.6011	1.9148	2.4127	0.9660	1.3523	1.5860	2.6312	1.5757	2.5942	1.5771	1.3521	1.5777	1.8413	1.7133	1.5228	1.2917	0.8990	1.5307	2.0116	1.7727	2.7708	0.9708	3.0497	1.8741
CUST_223_PI416573500	OR10V1	4.5438	2.8113	5.4469	5.1079	6.1771	4.8370	5.5861	5.0220	5.6395	5.2880	5.9879	4.7372	3.8481	5.2199	5.1715	4.7223	5.6004	3.9606	4.8400	4.0062	4.9014	4.9138	6.3583	5.3294
CUST_229_PI416379584	OR10V1	2.1137	3.4565	1.8584	1.3408	0.8969	1.9013	2.3664	1.7745	1.5032	2.1089	1.5963	1.8706	1.7589	2.1400	1.4917	1.2681	0.9387	2.9331	1.9719	1.9325	2.3621	1.4887	1.2072	2.6408
CUST_294_PI416573500	OR10W1	1.9503	2.2037	3.4755	1.5908	3.1367	2.1473	2.0793	2.6989	3.2705	3.1789	2.6909	2.0047	1.9069	2.1487	1.9325	1.6007	3.0979	1.8461	1.9524	2.0310	2.6811	1.5513	3.9625	1.7828
CUST_302_PI416379584	OR10W1	1.6412	1.9692	1.4843	1.0982	0.9159	1.8564	1.6643	1.5545	1.4234	0.7432	1.2775	1.7046	1.5303	1.7750	1.7104	1.1312	1.3505	1.4676	1.5919	1.8098	1.3723	1.1464	0.9336	1.4420
CUST_439_PI416573500	OR10X1	1.6650	1.9897	1.5521	1.1352	1.0176	1.8299	1.6840	1.5667	1.4984	0.7432	1.3948	1.7945	1.6029	1.8539	1.6804	1.1919	0.8573	1.5537	1.6181	1.8711	1.4352	1.1657	1.0369	1.4729
CUST_448_PI416379584	OR10X1	2.0779	2.1316	2.1805	1.1303	1.7685	2.2988	2.3353	1.7250	1.4820	0.7432	1.5214	1.6111	1.7616	1.8325	1.4458	1.1884	0.9011	1.5305	1.8526	1.8912	2.5444	2.1234	1.2089	2.3045
CUST_456_PI416573500	OR10Z1	1.6400	1.9606	1.5343	1.0988	0.9087	1.7366	1.6723	1.5445	1.5474	0.7432	1.3953	1.7117	1.6013	1.7974	1.7149	1.1403	0.9007	1.5203	1.5932	1.8304	1.4534	1.1149	1.0221	1.4692
CUST_465_PI416379584	OR10Z1	2.7906	1.8682	2.0850	0.8823	0.8166	1.4811	2.1395	1.5302	1.6809	0.7432	1.3756	1.5280	1.7040	1.5973	1.9846	1.1223	0.9465	1.4356	1.7211	1.7548	1.9941	0.8293	0.9735	2.5923

A_23_P59099	OR11A1	5.8918	2.9700	6.7247	5.8447	7.0044	4.6692	6.4299	5.8567	6.6374	6.3409	5.9028	4.4311	4.5797	5.5027	6.8817	5.2414	5.6784	4.3584	6.0142	4.3807	6.1714	5.9435	6.4509	5.2108
CUST_1360_PI 416261804	OR11A1	1.7023	2.1580	1.5582	2.6299	1.1037	2.0242	1.7290	1.6946	1.4654	0.7432	1.3828	1.8208	1.5799	1.9620	1.5189	1.2482	0.6991	1.5480	1.6427	1.9521	2.0792	1.2804	1.0642	1.4528
A_23_P37231	OR11G2	1.8175	2.2380	1.7251	1.3358	1.0727	1.9953	1.8832	1.7438	1.7065	0.7432	1.7136	1.9217	1.7499	2.0292	1.8045	6.1705	1.1459	1.7474	1.7291	2.0901	1.5804	1.3752	1.3543	1.6155
CUST_2155_PI 416261804	OR11G2	1.6672	2.0680	1.6721	1.1789	1.6733	1.8103	1.7419	1.7362	1.5873	0.7432	1.5479	1.8540	1.6663	1.9770	1.7719	1.2685	1.1523	1.6366	1.5981	1.8767	1.4440	1.1875	1.0641	1.4991
**CUST_2185_PI 416261804**	**OR11H1**	**1.7722**	**2.1248**	**1.6415**	**1.1946**	**1.1678**	**2.4216**	**1.8771**	**1.6895**	**1.5844**	**0.7432**	**1.5588**	**1.9051**	**1.7203**	**1.9712**	**1.7619**	**1.2714**	**2.4215**	**1.7053**	**1.7064**	**2.0333**	**1.4890**	**1.2310**	**1.8901**	**1.6006**
A_23_P128868	OR11H12	3.5491	1.9458	3.8528	2.6528	2.7311	2.8094	3.3809	2.1124	3.3742	0.7432	1.2964	1.6075	2.2449	1.7044	3.9195	5.7792	0.7903	2.3718	3.0284	1.8257	3.5109	0.9408	2.7205	3.7143
CUST_8751_PI 416261804	OR11H12	7.8178	6.8031	6.9693	5.0993	7.4488	6.7297	7.8078	6.7516	6.8804	6.1662	6.5908	5.6878	5.6831	6.8796	6.8075	4.3401	6.2477	5.6695	7.5822	6.4059	6.4673	5.2437	7.6274	5.6294
CUST_231_PI4 16573500	OR11H4	2.0081	2.0766	3.3005	1.1550	2.7226	2.5636	2.2301	1.6487	2.9086	2.3755	2.9080	1.8570	1.7916	1.9157	2.3923	1.2411	2.2801	1.7023	1.8716	1.9880	2.7367	1.1964	3.1392	1.7590
CUST_238_PI4 16379584	OR11H4	1.6460	1.9729	1.4836	1.1183	0.7450	3.7963	1.6908	1.5996	1.4797	0.7432	1.3495	1.7120	1.5866	1.8159	1.6031	1.0962	0.7829	1.4746	1.5747	1.7242	2.2617	1.1131	0.9803	1.4511
CUST_323_PI4 16573500	OR11H6	2.4855	2.0689	2.4808	1.4231	2.9282	2.1302	2.3080	1.7388	2.6067	3.8569	2.5588	1.8628	1.8946	2.0406	2.8956	1.4802	1.8591	1.7611	1.9851	2.5531	2.4772	3.4786	2.6976	1.7072
CUST_332_PI4 16379584	OR11H6	2.1759	2.0829	1.7955	1.9882	0.8317	2.1610	2.0147	3.1700	1.6555	2.5584	1.1871	1.9205	1.5894	2.6856	1.4938	1.0753	0.7112	2.6474	1.5966	2.2669	2.4052	2.1798	1.7081	3.2585
CUST_388_PI4 16573500	OR11L1	1.9841	2.1710	1.8689	1.4343	2.0658	2.0627	2.1708	1.7760	2.5479	0.7432	1.6280	1.9247	1.8167	2.0977	2.4399	1.6480	3.4983	1.6935	1.8777	2.0138	1.9808	1.4587	2.6831	1.6089
CUST_397_PI4 16379584	OR11L1	1.9970	2.3207	1.8746	1.0218	1.0127	3.2361	2.7545	1.8074	1.7257	0.7432	1.9956	1.7970	1.7214	1.8522	1.5317	1.2508	1.0582	1.6433	1.8145	2.1815	2.8341	2.2201	1.1643	1.8133
A_23_P93295	OR12D2	1.7355	2.0706	1.6172	1.2129	0.9597	1.8487	1.8775	1.7283	1.6210	0.7432	1.5472	1.8580	1.6793	2.0018	1.7684	1.2956	1.0527	1.6657	1.6239	1.8830	1.5970	1.2543	1.6039	1.5050
CUST_4660_PI 416261804	OR12D2	1.9869	2.2131	1.7248	2.1365	1.2564	2.6922	2.1115	3.2562	1.6151	0.9897	1.5964	1.9286	1.8315	2.5518	1.6841	1.5769	0.9011	1.9755	1.8189	2.0026	1.7551	2.1903	1.1647	2.4973
A_23_P259555	OR12D3	1.6743	1.8102	1.6421	0.9236	0.8892	1.5729	1.9171	1.4679	1.5165	0.7432	1.2299	1.5415	1.6430	1.6242	1.5149	1.1771	0.8214	1.4413	1.5989	1.6771	1.3812	0.9358	0.8345	1.4666
CUST_1925_PI 416261804	OR12D3	1.9245	2.2897	1.7825	1.6068	1.3794	2.3059	2.0270	1.8852	1.6839	0.8834	1.7253	2.0039	1.7312	2.1597	1.7825	1.7271	0.9688	1.6724	1.8146	2.1165	1.5792	1.5865	1.2264	1.5138
CUST_413_PI4 16573500	OR13A1	8.2324	7.9813	8.8178	8.2168	8.4808	8.4065	8.0031	7.6652	8.7238	8.4299	7.1848	8.2409	6.0425	7.7103	9.2034	7.0254	7.8380	7.9278	7.7097	7.3474	8.1597	8.5758	7.7697	7.9522
CUST_422_PI4 16379584	OR13A1	2.4787	3.9544	2.5664	2.5532	3.7407	2.5900	2.8462	4.9039	2.6303	2.7462	3.5069	1.6621	2.3068	3.4056	2.3726	3.1776	2.7216	2.1859	2.2342	3.7461	3.0494	1.4896	2.8525	2.6994
CUST_166_PI4 16379584	OR13C2	3.2667	2.6772	2.8948	3.1011	0.9908	3.7940	2.6763	3.4586	3.3211	3.6822	2.5480	3.3635	3.4998	3.9224	2.1848	1.4478	0.9930	3.4517	2.5985	3.4556	4.0057	3.5434	1.5756	3.2463
CUST_367_PI4 16573500	OR13C3	2.6146	2.3492	1.8380	1.5311	1.3233	2.2994	2.2413	1.9280	2.0696	0.8909	2.4445	2.0870	2.9201	2.9961	1.7769	1.6718	1.0397	1.7654	2.2291	2.1489	1.6567	1.6694	1.3217	1.7231
CUST_376_PI4 16379584	OR13C3	1.7201	3.6935	1.6258	1.3420	1.0350	1.9233	1.8362	1.6465	1.4368	2.3092	1.3689	3.6789	1.7204	1.8812	1.5799	2.4634	0.7321	1.6337	1.6102	1.9403	1.3758	1.2015	1.0176	1.5633

A_23_P169207	OR13C4	1.6656	1.7558	1.5209	0.8877	1.6241	1.5674	1.8945	1.4091	1.4019	1.6868	1.1031	1.5078	1.6044	1.5968	1.5427	0.9719	0.6916	1.4388	1.7435	1.5733	2.2343	0.9389	1.8227	1.4446
CUST_1030_PI416261804	OR13C4	2.9664	3.2906	2.1665	3.8333	3.7009	2.1635	3.0944	3.8310	2.5320	3.7679	3.2412	1.8629	2.1022	2.4072	2.4363	1.1607	2.6385	1.8749	2.8287	2.3284	2.6293	4.2123	3.2404	3.8658
CUST_161_PI416573500	OR13C5	2.2335	2.1839	1.6668	1.3473	1.2315	1.9462	2.4974	2.9960	1.7506	0.7432	1.9633	2.5223	2.0908	2.4990	1.7109	1.4500	2.3494	1.7082	2.0114	1.9880	2.6518	1.3138	2.5506	2.4944
CUST_239_PI416379584	OR13C5	2.4233	4.7594	3.4489	4.4189	3.3064	4.5486	2.7371	4.0419	4.0300	4.6891	2.7042	4.6385	3.0287	4.6885	3.6597	4.8321	3.7682	4.7897	2.7792	4.6162	3.5270	5.5242	3.2654	4.8957
CUST_387_PI416573500	OR13C8	3.8705	3.3886	4.0769	2.3437	3.9198	3.2847	4.6908	2.7740	4.4920	3.4772	4.0450	2.9486	2.5845	3.1136	3.9709	3.3909	3.3729	2.3180	3.1272	1.5502	3.6501	2.3935	3.7371	2.9196
CUST_396_PI416379584	OR13C8	1.7931	1.9762	1.4428	0.9324	2.1580	1.6893	2.0062	1.6352	1.4180	0.7432	1.3539	3.2442	1.6229	2.0150	1.4638	1.0896	0.7654	1.4431	1.6407	1.8128	1.3126	0.9826	0.9905	1.5720
CUST_160_PI416573500	OR13C9	2.2315	2.1487	1.8338	2.3874	1.2656	2.0801	2.6137	1.6860	2.1654	2.4611	2.2914	1.8882	1.8592	2.0729	1.7398	1.5183	2.3160	1.8571	2.1073	1.9697	2.3269	1.4808	1.4947	1.7090
CUST_165_PI416379584	OR13C9	1.6931	2.0894	1.6423	1.1667	0.8828	5.3001	1.8220	1.7614	1.5458	0.7432	2.2235	1.8530	1.7937	1.9547	1.7226	1.2418	1.0509	1.6439	1.6195	1.8932	1.7037	1.1753	1.8419	1.5267
A_23_P32006	OR13D1	2.1790	2.5908	2.9315	1.0739	2.7356	1.8333	2.4959	2.1349	2.4742	0.7432	1.7608	1.7591	1.8838	2.4681	2.2140	1.1352	3.0534	1.7062	2.0094	1.8713	1.7221	1.6570	2.6884	1.9329
CUST_793_PI416261804	OR13D1	2.0221	2.2136	1.8424	1.4794	3.5749	2.2236	2.0844	1.7355	2.9215	2.9360	1.8576	1.9160	1.8018	2.0938	2.3431	1.2700	2.5915	2.7492	1.8621	2.9961	3.2133	1.6232	3.4042	2.2092
CUST_278_PI416573500	OR13F1	1.6160	1.7886	1.4938	0.8866	0.7798	1.5421	1.7525	1.4226	1.4782	0.7432	1.1556	1.5331	1.5910	1.6061	1.5883	1.0377	0.7372	1.4239	1.5482	1.6363	1.4010	0.9077	0.8372	1.4223
CUST_286_PI416379584	OR13F1	1.8989	2.2261	1.6911	1.3157	1.0992	1.9926	2.0563	1.9620	1.6770	0.7432	1.7457	1.9850	1.7498	2.1004	1.6872	1.5128	1.0849	3.0118	1.7021	2.0417	2.1412	1.3307	1.2389	1.9347
CUST_1177_PI416573500	OR13G1	1.7310	2.1248	1.6385	1.2115	1.1724	1.9292	1.7736	1.7054	1.5740	0.7432	1.5901	1.9083	1.6802	1.9699	1.7576	1.2707	1.0410	1.6919	1.6837	2.0316	1.4695	1.2507	1.2413	1.5509
CUST_1216_PI416379584	OR13G1	2.2294	1.8853	2.3540	0.8661	0.9058	1.4738	2.5087	1.5670	1.9506	1.9288	1.3821	1.5492	1.7644	1.6006	1.4257	1.1847	0.9367	1.4864	2.0731	1.7923	3.2766	0.7432	0.9794	2.3721
CUST_389_PI416573500	OR13H1	6.1272	4.4881	4.7253	4.4830	6.0146	2.0422	5.9113	5.0006	4.9843	4.7726	5.4756	2.7293	3.8305	5.4040	3.8637	4.6603	5.2852	2.0730	4.4163	3.9377	4.7239	4.7552	6.0558	2.9672
CUST_398_PI416379584	OR13H1	2.5676	2.1827	3.5793	2.0354	4.7368	3.1119	3.0066	2.4423	3.9158	2.8754	3.4363	1.9188	2.0882	2.6282	3.5958	4.3052	3.7086	1.8005	3.2495	1.9343	3.7261	1.3977	4.9456	3.3091
CUST_279_PI416573500	OR13J1	3.3326	2.1710	4.2138	2.2695	4.6446	3.1676	3.4057	2.1580	4.2892	3.9155	6.4592	2.1338	3.0310	2.2245	4.3885	1.7648	2.4418	2.4251	3.0375	1.9545	3.5228	1.7437	4.1894	2.4578
CUST_287_PI416379584	OR13J1	1.7738	1.9353	1.3602	0.8153	0.7230	1.4647	2.0604	1.3504	1.5792	0.7432	1.0881	1.4309	1.6689	1.4745	1.4353	0.9462	0.7196	1.4288	1.9401	1.5398	1.7574	0.7432	0.8774	1.5827
CUST_498_PI416573500	OR14A16	2.3574	2.1633	4.0262	2.2942	3.6920	2.0997	2.6197	3.5984	3.3947	3.3369	3.4007	1.8711	1.9549	2.1129	2.9823	2.5143	3.4507	1.9715	2.4866	1.9029	3.7863	2.7487	4.3658	1.7679
CUST_509_PI416379584	OR14A16	3.2828	2.6142	3.0682	1.6951	3.0242	2.5642	3.9332	3.1571	2.3711	2.0230	1.8519	1.7250	1.8645	3.1135	1.4773	1.2210	2.6504	2.7950	3.4156	2.3109	3.4170	0.8293	2.9284	4.0953
CUST_497_PI416573500	OR14C36	1.6369	1.9438	1.6780	1.0808	0.9070	1.7251	1.6648	1.5286	1.5305	0.7432	1.3629	1.7046	1.5911	1.7792	1.7168	2.4124	0.8731	1.5098	1.5896	1.8135	1.4448	1.0971	0.9912	1.4614
CUST_508_PI416379584	OR14C36	2.7323	3.2101	3.8877	4.6901	4.8234	2.3060	3.5390	4.4277	4.1733	4.9014	3.8200	1.8833	3.0032	3.4717	4.1932	1.6060	3.5111	1.6076	3.3446	3.5894	3.9691	4.7654	4.7205	3.0160
CUST_618_PI416573500	OR14I1	3.2013	3.0767	5.3263	3.9672	5.3484	2.7061	2.9751	4.3357	5.1957	4.3486	4.9024	3.8228	1.9172	2.9693	4.0650	2.3685	3.6247	1.9039	3.8028	5.5656	5.6781	3.9868	6.1803	5.5440
CUST_634_PI416379584	OR14I1	6.3915	7.4384	7.3479	7.0071	7.5544	7.8045	5.5451	7.2130	6.9143	6.4704	7.1416	7.4453	2.8799	3.9436	4.0140	4.5505	3.3873	3.8251	7.0454	8.7553	8.1572	6.8814	8.0546	8.4505

CUST_499_PI416573500	OR14J1	2.1836	4.9405	2.6698	1.4855	3.0430	2.1212	2.2515	2.1651	3.2973	2.2529	2.1530	1.9088	1.8781	2.9980	2.7097	1.6072	2.8733	1.7465	2.4329	2.7724	3.1814	1.5522	2.9282	2.0847
CUST_510_PI416379584	OR14J1	2.0653	3.0917	1.6659	1.3196	1.1033	3.3326	1.6789	1.8104	1.6286	0.7550	1.6861	1.9021	1.6185	2.0266	1.7210	1.2848	1.0333	1.6880	1.6518	2.0971	1.4828	1.4151	1.2559	2.2673
A_23_P141665	OR1A1	1.6255	1.8740	1.4436	0.9962	0.8455	1.6502	1.7273	1.4976	1.4599	0.7432	1.2843	1.6171	1.5999	1.6841	1.6027	1.0799	0.8123	1.4640	1.5847	1.7446	1.3906	1.0087	0.9520	1.4502
CUST_2246_PI416261804	OR1A1	2.0643	2.3910	2.9858	1.6761	1.1691	1.9476	2.2580	1.7406	2.3034	1.4862	1.9810	1.8849	1.8455	2.0146	1.7537	1.3340	1.1346	1.6837	2.1990	2.1037	2.2988	1.2820	1.4665	1.8427
A_23_P130281	OR1A2	1.7808	2.1632	2.1358	3.0929	2.3293	2.1067	1.7644	1.7027	2.6622	0.8954	1.7632	1.9716	1.6960	2.0339	1.9313	1.2482	0.8698	1.7945	1.7313	2.0483	1.7149	1.4085	2.9316	1.5913
CUST_1546_PI416261804	OR1A2	2.1659	1.6786	1.9522	1.0550	4.1047	1.8296	2.4999	1.2929	2.5795	0.7432	3.2774	1.5835	2.0554	1.6834	1.9764	3.9444	3.0437	1.7172	2.0879	1.4534	3.1070	1.1935	3.4010	1.8975
CUST_500_PI416573500	OR1B1	1.8215	2.0946	1.7314	1.3812	1.2343	2.0375	1.9484	1.6800	2.0038	0.7432	2.0508	1.8738	1.7537	2.0293	1.7382	1.4977	1.0009	1.6790	1.7567	1.9418	1.5721	1.4185	1.0706	1.5223
CUST_511_PI416379584	OR1B1	1.6309	1.9225	1.7017	1.0979	2.0591	1.7683	1.6696	1.7452	1.4749	0.7432	1.3967	1.7373	1.7665	1.7903	1.8365	1.1744	0.8118	1.4976	1.7651	1.7819	1.4602	1.1164	0.9304	1.4370
A_24_P299769	OR1C1	1.7014	2.0618	1.6250	1.2091	1.2233	1.8381	1.7215	1.6420	2.1804	0.7432	2.2918	1.7801	1.6435	1.9101	1.7747	1.2232	1.3723	1.5871	1.6243	1.9237	1.4971	1.2298	1.1593	1.4960
CUST_5283_PI416261804	OR1C1	1.7855	2.0463	1.6916	1.1942	2.6115	1.8111	1.9280	1.6349	1.6367	0.7432	1.4951	1.7587	2.1959	1.8974	1.7647	1.2168	0.9680	1.5744	1.8696	1.9013	1.8473	1.2032	1.1724	1.5318
A_23_P27239	OR1D2	2.5412	2.3652	4.2142	4.1611	4.2934	2.4133	2.8387	3.1532	3.7487	4.6090	3.9753	1.9792	2.0574	3.8273	4.3185	2.0258	4.0073	2.0314	2.4174	2.8182	3.5420	4.4591	5.0352	2.0063
CUST_1335_PI416261804	OR1D2	2.0313	1.6825	3.1245	2.0183	4.7083	1.6437	2.3698	1.3047	2.9420	2.7525	2.6769	1.5641	1.8821	1.6713	2.0669	0.9324	2.5775	1.6324	1.9622	1.4870	3.2201	1.0364	3.3154	1.7908
A_23_P15832	OR1D4	2.0008	2.0133	2.2434	1.2542	1.0378	1.9739	2.1884	1.6014	1.8557	0.7432	1.5029	1.9181	2.2966	2.0888	1.7545	1.4328	1.1868	1.8874	1.8592	1.8463	2.8169	1.4650	1.7534	1.7814
CUST_1575_PI416261804	OR1D4	1.6585	1.9463	2.9749	0.8999	0.9051	2.4787	2.4756	2.1876	2.6312	2.3776	2.1637	2.7821	1.5976	1.6887	1.4308	1.2127	1.1063	1.9547	1.5611	3.1831	3.7181	2.5048	0.8397	1.5582
CUST_1102_PI416261804	OR1D5	5.1042	4.4872	5.2057	5.6913	5.4809	3.4650	5.5935	4.3794	4.9146	5.1558	5.1196	4.1594	4.0960	4.2085	5.1696	4.6438	4.7305	3.4019	5.2293	4.7499	5.4424	5.7338	5.3260	4.4267
A_23_P101073	OR1E1	1.8831	1.9735	1.9470	1.0664	3.2605	1.7804	2.0838	2.9050	2.3582	0.7432	1.5094	1.6894	1.7490	2.7291	1.9531	1.0474	1.3764	1.5584	1.7609	2.0248	1.7957	1.1042	1.0490	1.6019
CUST_1977_PI416261804	OR1E1	3.0326	3.2171	5.1534	4.6649	5.7785	3.9313	3.3226	4.9000	4.8831	5.5468	4.1568	4.0693	3.1685	4.6936	5.3268	3.1367	3.8442	3.8734	4.5210	3.7429	4.7817	4.5787	4.8172	3.9806
CUST_678_PI416261804	OR1E2	1.9203	1.9232	2.1912	1.1854	4.1525	1.8341	2.1374	1.5274	2.9632	2.4287	2.8755	1.6999	1.7516	1.8530	2.9513	1.1453	2.7261	1.5568	2.0915	1.7232	2.9888	1.1919	2.7386	1.8241
A_23_P318890	OR1F1	3.5088	3.4763	4.1102	3.1970	3.7107	2.8696	3.7988	3.5763	4.0415	3.7159	3.4030	2.9556	4.1613	2.0001	2.3231	3.1945	2.9519	2.4600	3.5012	1.8612	4.2924	3.7942	3.0927	3.3000
CUST_1380_PI416261804	OR1F1	2.0532	2.9104	1.9560	1.5226	1.3834	2.2654	2.1978	2.7485	1.9984	0.7550	1.6660	2.0703	1.8763	2.2279	2.6396	1.7037	1.4743	1.7845	1.8920	2.0747	1.7079	1.6738	1.2565	1.7084
A_23_P382506	OR1F2P	1.6951	1.8594	3.5441	2.5075	2.5430	1.7156	1.8623	2.7671	2.2885	3.6962	1.9456	1.6213	1.6462	1.7037	3.2463	1.0087	1.5070	1.5161	1.9114	1.6727	2.6229	3.7487	1.7216	1.4807

A_24_P58247	OR1F2P	2.4966	2.3568	3.6408	2.4153	3.5948	1.9436	2.8170	2.0593	4.0941	4.0245	3.1558	1.9121	2.1345	2.1971	4.1672	2.0479	2.6879	3.2010	3.1501	2.7320	3.6713	3.3506	4.2105	3.4709
A_23_P38634	OR1G1	1.8127	2.5017	1.7450	2.3554	1.1611	2.3519	1.7986	3.0771	1.6382	1.2748	1.6154	2.0020	1.8968	2.1646	1.6622	1.4805	0.9287	2.9767	2.8605	2.0639	1.6089	1.6891	1.2262	3.1110
CUST_9374_PI416261804	OR1G1	1.8581	2.1877	1.6705	1.2635	1.0858	3.3038	1.9706	1.7272	1.8646	0.7432	1.6904	1.8899	1.7586	1.9722	1.7924	1.2966	1.1414	1.7263	1.7657	2.0656	1.5464	1.2940	1.3279	2.8859
CUST_152_PI416573500	OR1I1	1.9385	2.3478	1.9899	1.2860	1.8577	1.9191	2.7237	1.6216	2.2078	2.2217	1.4489	1.8486	3.0338	2.0081	1.6890	1.3953	1.8539	2.0489	1.9946	2.0063	2.3266	1.2832	2.8103	1.8767
CUST_157_PI416379584	OR1I1	3.9663	2.1173	4.0933	2.8421	3.2547	2.4741	3.7197	1.7609	3.8065	3.2893	3.7463	2.8541	2.2008	2.8362	3.1274	1.3992	3.1804	2.8578	3.6607	2.8559	4.6066	3.1395	4.1182	2.9151
CUST_311_PI416573500	OR1J1	1.6094	1.8370	1.4655	1.0445	1.6533	1.6765	1.6631	1.4650	1.5082	0.7432	1.2828	1.6043	1.5706	1.7406	2.1598	1.0416	0.7705	1.4460	1.5414	1.6458	1.4425	1.0449	0.9697	1.4316
CUST_319_PI416379584	OR1J1	2.7002	2.3364	2.3011	1.3463	1.2212	2.8132	3.3832	2.1400	1.6002	1.9777	1.6242	1.9265	2.7771	3.6902	1.5845	1.6101	1.0384	2.9586	2.9817	1.9802	2.4456	1.2920	1.2777	3.9508
CUST_183_PI416573500	OR1J2	1.7408	1.9860	1.8084	1.2291	1.0669	1.9051	1.8308	1.5694	1.5184	0.7432	1.3978	1.8102	1.7047	1.9371	1.5879	1.3147	0.8442	1.5939	1.6998	1.8093	1.5088	1.2784	1.2472	1.4588
CUST_188_PI416379584	OR1J2	2.5859	2.2034	2.4640	1.2593	2.4462	3.7464	3.0053	3.1066	2.9742	2.0430	2.4747	1.7729	2.8549	2.0276	1.7268	1.6422	1.0605	1.7755	2.3070	3.5776	3.1473	1.2368	2.1312	2.9758
CUST_377_PI416573500	OR1J4	3.8604	5.2530	3.7383	4.3286	4.6355	2.7739	2.9002	4.1040	3.6490	4.2451	4.1081	1.7483	2.2094	3.3683	3.6446	4.9708	4.2429	2.8335	2.9504	4.2655	3.3231	3.4205	4.2636	2.0862
CUST_386_PI416379584	OR1J4	4.4058	4.6661	5.7083	4.7185	6.0894	3.9475	4.3603	3.9544	5.6769	5.3121	4.8586	3.3880	2.3310	5.6481	5.6738	3.0898	5.0434	4.0060	5.3538	4.5148	5.0749	5.1441	5.9996	3.9757
CUST_162_PI416573500	OR1K1	4.9224	2.3696	4.4158	3.4874	4.8292	3.0882	5.1898	4.0757	4.6310	3.4456	4.0264	2.2609	3.2191	4.6192	4.4445	3.9664	3.9591	1.9804	5.0950	1.8992	3.9707	3.8530	5.1338	3.2410
CUST_167_PI416379584	OR1K1	1.8394	2.3434	1.7580	2.5279	1.3097	2.0859	1.8879	1.8015	1.7117	0.7550	1.8529	2.1433	1.7730	2.0894	1.9087	1.3976	2.3996	1.8677	1.7787	2.2365	1.6432	1.3872	1.4055	1.6697
CUST_300_PI416573500	OR1L1	1.6598	1.9540	1.8587	1.1648	0.9662	1.8391	1.7031	1.5262	1.4775	0.7432	1.3168	1.7361	1.5866	1.8287	1.6725	1.2312	0.7912	1.5106	1.6198	1.7976	1.4703	1.1949	0.9096	1.4288
CUST_308_PI416379584	OR1L1	4.0204	2.0622	2.7474	1.1389	0.6953	3.1642	3.1730	1.5468	2.1939	1.0992	1.2715	2.9800	1.9261	1.5827	1.3386	1.0436	0.8395	2.4288	2.3866	3.4598	3.4361	2.3234	0.9927	4.0774
A_23_P94647	OR1L3	1.6161	1.8909	1.4447	0.9900	0.8587	1.6528	1.7344	1.5189	1.4368	0.7432	1.2861	1.6127	1.5822	1.6786	1.5983	1.0868	0.7405	1.4404	1.5761	1.7558	1.3735	0.9897	1.8531	1.6054
CUST_2173_PI416261804	OR1L3	1.7517	3.2861	1.5135	0.9426	0.7674	1.7030	1.6584	1.9394	1.3799	0.7432	1.2568	1.5193	1.5377	3.3658	1.4873	1.5409	0.6745	1.6461	1.5225	1.6904	1.4856	0.9964	0.8422	1.5873
A_23_P83244	OR1L4	1.8276	2.4001	2.0077	1.1750	1.8379	1.9682	1.8880	2.0930	2.1890	0.7432	1.6926	2.0482	1.7692	2.0594	1.7285	1.4386	1.1063	1.6672	1.7141	2.2394	1.5595	1.1499	1.1706	1.7215
CUST_951_PI416261804	OR1L4	2.0181	4.1168	4.1880	3.4366	1.1175	4.8741	3.2009	2.9547	3.4394	1.3889	2.5194	4.1335	1.8370	4.2204	3.2309	4.3057	0.8741	4.6811	1.9833	3.7057	4.1163	3.1497	1.8117	4.0534
CUST_1649_PI416261804	OR1L6	1.8817	4.0273	2.9427	2.3963	0.9493	3.6612	2.9900	2.6198	2.5075	0.7432	1.9099	3.9426	1.6927	3.8009	1.6631	2.3866	1.0120	3.6074	2.1115	4.2099	3.7967	3.9380	1.2628	3.9545
CUST_435_PI416573500	OR1L8	1.5969	1.9663	1.6697	1.0936	1.1972	1.6721	1.6788	1.5628	1.8630	0.7432	1.3421	1.6941	1.5826	1.8213	1.7244	1.1444	0.8651	1.4843	1.5462	1.7393	2.1709	1.0537	0.9182	1.4300
CUST_444_PI416379584	OR1L8	1.5908	2.1375	1.5279	1.9265	1.5979	1.7150	1.7425	2.5212	1.5229	2.3017	2.0321	1.7865	1.6187	1.9288	1.6775	3.2644	1.1475	1.5368	1.5411	3.0845	1.3611	3.3515	1.0393	2.0944
CUST_312_PI416573500	OR1M1	2.1257	2.4423	1.7796	1.2377	1.1769	2.4783	2.3633	2.5535	2.1077	1.0034	2.9836	2.1109	1.9273	2.0541	1.9363	1.4233	1.4845	1.8182	1.9968	2.7843	1.8113	1.2066	1.3824	3.5059
CUST_320_PI416379584	OR1M1	1.7782	2.3193	1.7246	1.2165	1.2521	2.0391	1.8467	2.0059	1.6334	0.8231	1.6546	1.9952	1.7502	2.1006	1.8022	1.3052	1.1555	1.8301	1.7318	2.2515	1.5818	1.2562	1.4025	1.6619

CUST_265_PI416573500	OR1N1	2.0236	2.0759	3.2884	1.3003	3.3002	1.9992	2.1891	1.6284	2.8555	2.3606	1.4967	1.7851	1.8026	1.9582	2.2419	1.2474	2.6460	1.6745	1.8562	1.8952	2.7578	1.3298	2.1424	2.0360
CUST_273_PI416379584	OR1N1	2.7071	2.0741	2.1389	1.8071	3.5900	1.8594	2.6906	2.8016	3.0158	0.9414	1.9620	1.7601	2.5564	2.1352	1.8022	2.3171	0.7358	1.7114	2.4317	1.8668	2.4512	1.2524	3.0542	1.9273
A_23_P135226	OR1N2	2.2668	1.8775	2.7249	1.3653	2.7281	1.5857	2.6619	1.5446	2.2962	1.2779	2.7078	1.5649	1.8399	1.7177	1.5168	1.3244	0.8349	1.5769	1.9714	1.6855	3.1543	0.9964	3.3018	1.8756
CUST_2254_PI416261804	OR1N2	1.8408	2.2067	2.1216	1.1544	2.9567	1.8905	1.9964	1.6981	1.6699	0.8404	1.4426	1.8863	1.7659	1.9229	1.7089	1.1531	0.9594	4.5716	1.7926	1.9381	1.6444	1.1753	1.4197	1.6666
A_24_P283591	OR1Q1	1.6853	1.9832	1.6673	1.2617	0.8315	1.9439	1.5966	1.5221	1.6158	0.7432	1.2977	1.8170	1.6424	1.9520	1.8459	1.0810	0.7196	1.5985	1.6145	1.7661	1.6649	1.3035	0.9972	1.5166
CUST_1905_PI416261804	OR1Q1	2.7590	3.1852	2.4110	1.4067	1.9154	2.0130	3.2202	2.8955	2.3417	1.8882	1.5687	1.8777	2.0488	2.0550	1.8293	1.4720	0.9419	2.4792	2.7449	1.9966	3.1312	1.3375	1.3416	3.3248
CUST_220_PI416573500	OR1S1	3.9135	2.0718	4.0001	1.2963	3.4085	1.9785	3.8135	1.5795	4.0558	2.7575	3.0706	1.8001	2.1068	1.9712	3.6728	1.3429	0.8420	1.8227	3.4983	1.8784	3.0524	1.3699	3.7646	2.2891
A_23_P13294	OR1S2	2.3895	2.0139	3.5919	1.5634	3.1205	1.9576	2.7565	2.8242	2.8796	2.9408	3.3095	1.7818	2.0124	2.2756	1.7370	1.2621	3.2135	1.7817	2.4446	1.8266	2.4618	1.3472	3.7001	2.0494
CUST_1655_PI416261804	OR1S2	2.3565	3.2889	2.1018	1.2042	0.8223	4.2114	3.3063	2.6370	2.6009	1.4495	1.4992	1.8812	1.7916	2.2794	1.3703	1.1799	0.9222	3.7870	2.0641	3.0889	3.5201	2.3675	1.1286	2.9196
CUST_226_PI416379584	OR1S2	1.7830	3.3059	1.9881	2.2547	2.1605	1.7774	2.0494	3.1012	2.0107	0.7432	1.4853	1.6245	1.5530	3.7760	1.4158	3.0291	0.8566	2.4825	1.7007	2.6723	2.7855	1.3262	0.9795	3.6213
CUST_140_PI416573500	OR2A1	3.7027	3.1779	5.1506	4.0948	4.9569	3.1733	3.3395	4.4405	5.1616	5.1775	3.8199	3.9171	3.2052	6.5541	5.5058	3.4701	4.0764	2.4961	3.7043	3.5781	4.9572	4.3891	4.5539	3.7965
CUST_145_PI416379584	OR2A1	2.0666	2.1243	1.7752	3.0753	3.7830	1.9099	2.3315	1.8177	1.6866	4.2358	3.1700	1.9165	1.8953	2.0427	2.1526	1.3262	2.5140	1.7981	1.9033	1.9394	1.7388	1.3029	3.2248	2.4810
CUST_609_PI416573500	OR2A12	2.3701	2.3766	2.8563	2.6136	4.3143	1.9960	2.5788	2.9406	2.8470	3.5810	2.8155	1.9066	2.1061	2.9649	2.8272	2.0447	3.1912	1.9330	2.3867	2.2549	3.2216	1.1287	3.0814	2.0458
CUST_625_PI416379584	OR2A12	1.9273	1.9759	1.9478	0.8802	0.8702	1.5196	2.2774	2.6909	1.3454	0.7432	1.1805	2.4747	2.0631	1.5501	1.4182	1.1453	0.8153	1.4526	1.9361	2.8592	2.9336	0.8610	0.8487	2.0556
A_23_P59783	OR2A14	1.8502	2.1073	1.5593	1.1367	1.0773	1.9036	2.0136	1.6940	1.4828	0.7432	1.4843	1.8264	1.7172	1.9190	1.6167	1.2248	0.9027	1.6401	1.7349	1.9908	1.4408	1.1879	1.2280	1.9129
CUST_5278_PI416261804	OR2A14	5.2403	4.6338	5.4749	4.6246	6.4450	5.3130	5.7072	5.6032	5.5552	5.2451	5.3544	3.2102	3.5585	5.3163	4.4516	3.8608	4.8159	2.9720	4.9466	2.4085	5.0796	4.5496	7.3396	3.5548
CUST_608_PI416573500	OR2A2	1.7734	2.0779	1.8942	1.1890	1.4076	1.8479	1.8851	1.8053	2.4901	1.3267	1.4701	1.8606	1.6910	1.9142	1.8141	1.2512	2.8800	1.6530	1.7008	1.9739	2.1804	1.2083	3.9528	1.5629
CUST_624_PI416379584	OR2A2	4.7658	4.5587	4.6099	3.7812	4.8853	5.5450	4.3428	4.9826	5.1196	4.4843	4.3459	3.9896	3.5912	5.2143	3.9566	4.2557	3.6099	2.7804	4.1385	3.0921	5.3219	3.8523	5.1574	3.8730
A_24_P506816	OR2A20P	1.6139	1.8209	1.7996	0.9963	1.0402	1.6585	1.7179	1.4365	1.6316	0.7432	1.1619	1.5813	1.5762	1.6600	1.9857	1.5109	0.7022	1.4507	1.5755	1.6610	1.4091	1.0174	0.8523	1.4243
CUST_7315_PI416261804	OR2A20P	3.4028	2.4878	3.2961	2.8990	2.6666	1.7377	2.9604	1.2600	2.9596	3.4931	2.3849	2.6368	2.4256	1.6670	2.6601	3.4395	2.4714	1.6491	2.9472	1.4809	3.1842	1.2449	2.8352	2.4160
A_24_P52293	OR2A25	1.9160	2.3921	1.6875	1.2869	2.8635	2.0537	1.9129	1.6357	1.6468	0.7432	1.6751	2.0013	1.7876	1.9944	1.8113	1.3172	0.8491	1.7597	2.0001	1.9981	1.6776	1.3343	1.2870	1.7035
CUST_13131_PI416261804	OR2A25	3.0495	2.3170	2.2919	1.2028	1.1305	2.3722	3.1111	2.0262	1.4609	0.7432	1.4948	3.2149	2.0434	1.8852	1.4542	1.4795	1.0080	2.8906	2.3924	1.8588	2.5402	1.2335	1.1929	3.2613

A_23_P134566	OR2A4	1.7159	2.0573	2.3462	1.8395	1.0466	1.9539	1.7139	1.5923	2.3052	1.6480	1.9222	1.8619	1.6396	1.9544	1.6576	1.2094	0.7689	1.6437	1.6631	1.9124	1.9360	1.2806	2.6095	1.4987
CUST_767_PI416261804	OR2A4	1.8341	2.1648	2.5130	1.2455	3.3961	2.0474	1.9023	1.6596	2.4296	2.0476	1.4917	1.9799	1.8044	2.0615	1.8374	1.2459	2.7574	1.8785	1.7838	2.0838	2.5629	1.3196	2.8348	1.6916
CUST_508_PI416573500	OR2A5	3.8244	2.0780	4.5698	3.0484	2.6569	4.5670	3.4349	2.6577	4.0855	2.1294	1.5187	3.9767	2.4318	2.1390	1.5571	4.8411	0.8988	3.7099	3.6053	3.1240	4.3878	3.0703	2.2482	5.3966
CUST_519_PI416379584	OR2A5	1.8161	2.1508	1.7907	1.3021	1.0519	1.9297	1.9507	1.8585	1.6689	0.7432	1.6857	1.9725	1.7711	2.1026	1.7784	1.4409	1.1928	1.7513	1.6776	1.9735	1.4997	1.3262	1.1962	1.6354
A_23_P123172	OR2A9P	3.4885	1.9593	3.0600	3.8495	0.8283	1.8955	3.9791	2.1282	2.8966	2.6062	1.0653	1.6414	2.8594	1.7489	1.8130	4.6457	1.9071	1.9718	6.7354	1.7333	3.0740	1.8017	1.7226	2.9429
CUST_301_PI416573500	OR2AE1	1.7255	2.1628	1.6654	1.2072	2.3317	1.9516	1.7637	1.7036	1.5917	0.7432	2.5001	1.9284	1.6828	2.0215	1.7503	1.2742	1.0724	1.7004	1.6768	2.0531	2.3993	1.2502	2.4507	1.5285
CUST_309_PI416379584	OR2AE1	1.6865	2.9727	1.9854	1.0524	1.4470	3.0759	1.8967	1.6457	1.5999	0.7432	1.4966	1.8381	1.6490	2.7968	1.6717	1.2636	1.0262	2.2060	1.6659	1.9557	2.5582	1.1015	1.0628	1.5286
CUST_171_PI416573500	OR2AG1	1.6982	1.8962	1.9386	2.0528	2.5633	2.8003	1.8051	1.7954	2.2812	1.6144	1.6689	1.7035	1.9963	2.5199	2.0867	1.0905	0.6905	1.5395	1.6476	1.7427	1.5918	3.6866	2.5507	1.4839
CUST_176_PI416379584	OR2AG1	1.7796	1.9466	1.8605	0.9211	1.9203	2.1428	2.0764	2.4008	2.5588	2.8165	2.3515	1.5886	1.6299	1.6748	1.6087	1.1461	0.8047	1.7542	2.1420	2.7712	2.4153	2.6293	0.8836	1.5551
CUST_67_PI416573500	OR2AG2	1.9271	2.4859	1.9263	1.4885	1.2615	2.2122	1.9703	1.7941	2.3958	0.7688	1.8282	2.2524	2.0067	2.1720	1.9748	1.3906	1.2232	1.9349	3.0508	2.2791	1.7545	1.5676	1.5320	1.7304
CUST_71_PI416379584	OR2AG2	3.5461	3.5543	5.4296	4.3513	6.3703	4.2663	3.8823	4.8700	5.3170	4.6467	4.5861	1.8776	3.0421	4.6580	5.5814	3.3359	4.2846	3.4597	4.3726	4.1213	4.7816	4.3254	5.4891	3.8816
CUST_174_PI416573500	OR2AK2	1.7175	1.7452	1.5904	1.0400	1.7252	1.8093	1.7763	2.8493	1.6065	0.7432	1.4975	1.5523	1.6448	2.0392	1.5245	0.8618	2.4173	1.5618	1.7427	1.5087	1.5083	1.1753	1.7227	1.5324
CUST_179_PI416379584	OR2AK2	5.6481	6.0102	3.6384	2.6299	3.6505	3.7655	4.8484	5.9294	3.9800	3.9760	4.0871	3.8114	4.8333	5.4180	3.1940	3.6050	4.2931	4.2074	4.5874	5.8642	3.6884	3.2842	4.4978	4.4582
CUST_47_PI416573500	OR2AT4	2.3653	2.3161	4.0542	3.1041	4.5116	2.5513	2.3325	3.6336	3.9149	4.2893	3.4477	1.8894	2.2906	3.7947	2.2420	3.0976	4.1606	1.7570	2.7030	3.0184	3.5808	3.7692	4.4338	2.5476
CUST_50_PI416379584	OR2AT4	2.1424	1.9580	1.9556	2.8644	0.8441	2.1784	2.3496	3.3032	2.2387	2.9823	1.2897	1.4653	2.3694	1.6684	1.4486	2.2944	2.4124	2.0011	2.3216	3.2724	1.6032	3.1858	0.9506	2.3694
CUST_945_PI416573500	OR2B11	2.6140	2.0553	1.8291	1.3503	1.6444	1.9873	2.1143	1.4938	2.2889	1.2744	2.6614	1.8775	1.9018	2.0138	1.8869	1.2954	1.1545	1.7827	1.7953	1.8931	1.8196	1.3736	1.3762	1.6412
CUST_976_PI416379584	OR2B11	7.1754	6.6114	7.2898	6.4407	7.4128	6.1964	6.9804	6.5311	7.2863	6.9637	6.3699	6.2917	5.3698	6.4597	7.5657	5.3850	6.4845	5.8488	7.0648	6.5210	6.7417	6.8122	7.2713	6.0477
A_23_P111088	OR2B2	3.6072	2.3132	4.2254	4.5090	4.7398	2.0449	3.4951	3.9073	4.8576	4.0621	3.3873	1.9274	2.8874	3.5834	4.0409	3.4136	4.4191	2.4574	2.9562	2.1274	3.4284	3.2896	4.7769	2.5681
CUST_1445_PI416261804	OR2B2	1.9088	2.2874	1.7370	1.4258	0.9797	2.1940	1.9488	1.7819	1.7712	0.7432	1.6882	1.9265	1.7490	2.0625	1.9350	1.3481	0.9049	1.9484	1.7562	1.9925	1.5825	1.4926	1.3299	1.6069
CUST_1096_PI416573500	OR2B3	1.7582	2.0582	1.6549	1.2839	0.8482	3.2374	1.8461	1.6582	1.6407	0.7432	1.5706	1.7869	1.6950	1.9399	1.6742	1.2766	0.9485	1.5484	1.6452	1.8344	1.4917	1.2685	1.1933	1.4807
CUST_1132_PI416379584	OR2B3	1.7401	2.2653	1.6241	1.2060	1.1451	2.0775	1.7807	1.8126	1.5074	0.8785	1.5465	1.9030	1.6512	2.0416	1.6469	1.2619	0.9004	1.6904	1.6744	2.1349	1.4436	1.2833	1.2722	1.5595
A_23_P70468	OR2B6	1.9895	2.3071	1.8022	1.2820	1.2292	1.9940	2.1242	1.8759	2.0416	0.7432	1.7847	2.0874	1.9904	2.0315	1.8451	1.3606	1.3205	1.8275	1.8880	2.1903	1.6234	1.9997	2.2039	1.7743
CUST_1911_PI416261804	OR2B6	2.3421	2.5493	1.6368	1.2955	1.2259	1.8800	2.6324	2.1647	1.6063	0.7432	1.6268	1.7851	1.9628	2.0177	1.5746	1.5983	1.0816	1.6567	2.3167	1.8829	1.6105	1.2609	1.1903	1.9487

A_23_P163737	OR2C1	6.0477	1.9752	2.3471	1.5293	2.5033	1.8077	2.9921	3.0818	3.0380	1.0436	2.6584	2.6006	2.1622	3.1274	1.7027	2.8251	3.8568	2.5413	2.8348	2.4539	2.9476	1.2508	3.0842	2.5022
CUST_1710_PI416261804	OR2C1	2.8992	3.1810	4.0209	2.1872	4.8132	2.7317	4.1634	4.1056	4.5819	4.0349	3.9459	1.8857	3.0629	3.2550	4.1177	3.7682	3.5578	1.8510	2.6502	2.0281	4.3198	2.5239	4.3366	2.1524
A_23_P115563	OR2C3	1.9681	2.2914	1.6157	1.2392	1.0664	1.9415	2.1143	1.8152	1.5677	0.7432	1.7413	1.8903	1.7642	1.9729	1.6915	1.3064	1.1112	1.6843	1.8248	2.1275	1.5353	1.2802	1.3038	2.0933
**CUST_421_PI416573500**	**OR2D2**	**2.0109**	**2.1339**	**1.8921**	**1.2925**	**1.0018**	**2.0041**	**2.1715**	**1.8760**	**1.8706**	**2.5623**	**2.0253**	**2.0174**	**1.8996**	**2.1187**	**4.4633**	**1.4549**	**1.1586**	**1.8018**	**1.8030**	**1.9580**	**1.5570**	**1.3845**	**1.2851**	**1.8338**
**CUST_430_PI416379584**	**OR2D2**	**3.2238**	**2.4571**	**2.7703**	**1.7039**	**2.3632**	**2.8854**	**3.5139**	**4.2204**	**2.5740**	**2.2694**	**2.0049**	**3.6109**	**3.5970**	**2.2643**	**1.7942**	**1.8082**	**3.1573**	**1.7740**	**2.9060**	**3.5163**	**3.1798**	**1.4839**	**1.7885**	**3.3509**
CUST_436_PI416573500	OR2D3	1.8099	2.0964	2.0915	3.2677	1.1738	1.9502	1.9616	1.7060	1.5772	0.7432	1.5404	1.8433	1.7461	2.0050	1.7051	1.4650	1.0198	1.6555	1.7283	1.9383	1.5380	1.3402	1.0548	1.5340
CUST_445_PI416379584	OR2D3	2.8270	1.7974	2.4076	1.8521	1.9048	2.2508	2.8946	1.4395	2.4248	2.4886	2.2310	2.8036	1.8201	1.5420	1.4540	1.1494	0.7196	1.9898	2.2768	1.6467	3.3430	0.7688	2.4268	2.0398
A_23_P8716	OR2F1	2.0004	2.2738	2.4363	1.1817	3.0364	2.0622	2.0554	1.9212	2.0358	0.7432	1.6124	2.0002	1.9021	2.0250	1.6744	1.2812	1.9086	1.8186	1.8737	2.1013	1.8718	1.2604	2.6283	1.8703
CUST_5751_PI416261804	OR2F1	2.2608	2.0871	2.7939	1.1704	4.0514	1.8991	2.5553	1.8676	2.6865	2.2945	2.4023	2.2270	1.9660	2.0443	2.3869	1.4506	1.0116	1.7047	1.9648	1.9069	2.2136	1.3147	5.1648	2.0175
CUST_13_PI417507815	OR2F2	2.3132	2.2194	2.7184	1.5390	3.0063	2.3944	2.5127	2.7107	2.2832	0.8919	2.7016	2.0423	2.0000	2.2025	2.0889	1.4722	3.3018	1.9517	2.5761	1.9876	1.7563	1.8457	3.3101	1.9098
CUST_591_PI416573500	OR2G2	1.7274	2.0592	1.9260	1.2093	1.7627	1.9695	1.7640	1.6062	1.7353	0.7432	1.9302	1.8885	1.6804	1.9516	1.7774	1.2424	1.1395	1.6581	1.6821	1.9420	1.5064	1.2479	2.4478	1.6973
CUST_607_PI416379584	OR2G2	3.8804	3.2587	3.3284	4.1388	1.0956	2.8728	3.3122	2.7227	2.9714	0.7432	1.3466	2.8313	1.7820	3.4034	1.2790	1.2909	0.7795	3.2779	3.1202	3.3187	4.5433	3.1023	3.3234	4.6660
CUST_136_PI416573500	OR2G3	1.9879	2.6469	1.7974	2.1013	1.7234	1.8592	2.2353	1.8716	1.7011	1.1909	1.6698	1.9248	1.8426	2.0531	2.3255	1.4035	1.2208	1.7213	1.7815	1.9677	2.3934	1.2312	1.1949	1.8040
CUST_141_PI416379584	OR2G3	1.8228	1.8664	1.6073	0.9945	0.7825	1.8565	2.0810	1.5389	1.4191	0.7432	1.2760	1.4936	4.7528	1.5869	1.4196	1.0367	0.7630	1.4458	1.6782	1.7130	2.0682	0.9342	0.9731	2.5474
CUST_459_PI416573500	OR2G6	3.2515	2.2849	2.1840	2.7051	2.7942	1.7397	3.3553	2.6635	2.9726	3.8133	2.8734	1.8533	1.8362	2.2898	2.7168	1.3805	1.2991	1.7834	2.8721	3.4211	3.2371	1.0876	3.4229	2.3353
CUST_469_PI416379584	OR2G6	1.8079	1.9941	1.4209	0.9451	1.1315	1.6470	2.1056	1.9697	1.6004	0.7432	1.3753	1.6632	1.7257	1.7397	1.5589	1.1227	1.4265	1.8408	1.8909	2.7284	1.4186	1.4880	0.9813	2.4249
A_23_P111092	OR2H1	9.5472	8.8923	10.3272	9.6335	9.9758	8.7050	9.9908	8.7127	10.2799	9.8942	8.8389	8.6415	7.5909	9.0042	10.6430	8.4407	8.7488	8.6059	9.1329	8.6374	9.8942	10.3276	9.3230	8.2507
A_24_P940897	OR2H1	1.9953	3.7963	1.9152	2.7548	1.1337	3.8738	2.9317	1.7819	2.1068	2.7422	2.3104	1.8194	1.6248	3.5012	1.5434	1.5262	0.8646	1.9391	1.9659	4.2819	2.9465	1.3813	2.1960	2.8721
A_23_P122485	OR2H2	5.1678	2.5199	4.6849	3.9293	6.5675	3.4115	4.9808	5.9010	4.8033	5.1333	5.1428	1.9082	4.1801	5.6849	4.6750	4.1717	4.4211	3.2816	5.0687	3.2464	4.7255	5.0094	5.9659	3.2751
A_23_P307274	OR2H2	2.2057	1.9658	2.3849	0.9699	3.3996	1.5485	2.6315	1.6627	2.5791	0.7432	1.5943	1.7168	2.1825	1.8731	1.9769	1.3316	1.1735	1.6069	1.9223	1.8091	3.1778	0.9638	2.9396	1.9140
A_23_P19428	OR2J2	1.6086	1.9139	1.6209	1.0821	0.8912	1.7197	1.6596	1.4825	1.4777	0.7432	1.2587	1.6842	1.5542	1.7688	1.7023	1.1529	0.7591	1.4697	1.5747	1.7507	1.4579	1.0904	1.5446	1.4300
CUST_6419_PI416261804	OR2J2	1.6541	1.7899	1.4099	0.9938	0.7737	1.7028	1.7243	1.4089	1.4225	0.7432	1.1439	1.5820	1.6064	1.6465	1.5526	0.9763	0.7095	1.4941	1.6021	1.6438	1.4130	1.0642	0.9539	1.4756
CUST_692_PI416573500	OR2J3	1.6691	1.8089	2.3241	1.5359	2.9022	1.7764	1.7520	1.3964	2.0278	0.8652	1.0752	1.5747	1.5846	1.6506	1.9942	1.1953	0.6745	1.4863	1.6177	1.6220	1.4110	1.1158	2.3469	1.4588
CUST_717_PI416379584	OR2J3	1.7607	2.0845	1.5237	1.1535	0.9433	1.8902	1.8643	1.7109	1.5288	0.7432	1.4891	1.7133	1.6598	1.8474	1.6031	1.1675	0.8503	1.5534	1.6561	1.9229	1.4131	1.2167	1.1516	1.5198

CUST_157_PI416573500	OR2K2	1.7338	2.2756	1.8457	1.1275	0.8303	1.8617	1.8621	1.8075	1.8978	0.7432	1.5291	1.9031	1.7444	1.9397	1.7224	1.5835	1.0975	1.7395	1.7011	2.0353	1.5369	1.1314	1.9251	1.6406
CUST_162_PI416379584	OR2K2	2.1914	2.3456	1.4927	1.2311	0.8356	2.7589	2.5394	2.6077	1.4446	0.7432	2.2440	1.8348	1.8916	1.9432	1.6182	1.1475	2.6371	1.7684	2.0697	2.7958	1.9445	1.1862	1.6436	3.0898
A_23_P370786	OR2L13	2.7939	2.9999	3.5958	3.5102	4.3891	3.2386	3.0616	4.3656	3.5705	3.8132	3.9899	3.3633	3.3975	3.3833	3.0083	2.6274	4.0098	3.2324	2.6150	2.2486	3.7267	3.7202	4.0043	4.7390
CUST_1599_PI416261804	OR2L13	3.6517	4.0883	2.8961	2.4320	2.9228	2.8354	3.6268	4.5428	1.7703	1.9377	3.3837	1.9354	3.6002	3.9892	1.6669	1.3470	3.4104	2.4851	3.6517	4.6313	2.1319	1.2915	2.2458	2.5267
CUST_191_PI416573500	OR2L2	2.7955	3.0079	2.3738	1.1558	2.8555	2.0504	3.8673	3.3552	1.9276	3.1596	2.7334	1.6625	2.2014	2.2695	1.9457	1.0419	2.3580	2.1512	2.4977	2.8237	2.7467	1.9200	2.8103	4.6755
CUST_196_PI416379584	OR2L2	3.6055	4.7160	3.6775	3.2953	4.4453	3.9810	3.8671	4.4904	3.3424	4.1657	3.0024	3.5261	3.5406	4.8146	2.5768	3.0862	3.6922	3.1760	3.4185	5.1676	3.3727	2.2994	4.7330	3.3696
CUST_171_PI416379584	OR2L3	6.3308	7.7155	5.5855	5.6080	6.5338	6.0187	6.8139	7.5552	5.4516	5.3850	6.1339	5.4364	6.4627	7.7507	5.5933	4.5809	6.1662	5.2993	7.0144	7.4487	5.5010	4.2668	7.0697	6.8584
CUST_222_PI416573500	OR2L3	3.2476	3.6347	3.3479	1.9437	2.9815	2.4825	3.4368	4.3999	2.8706	4.0516	3.0312	3.5108	2.7299	2.1519	3.1174	2.2364	3.6769	2.2647	3.2261	4.6756	2.9573	1.8593	3.9231	3.8351
CUST_166_PI416573500	OR2L8	3.7904	2.9769	3.8292	3.3494	3.5357	4.0410	4.2221	4.5937	3.4020	3.9024	3.9056	2.4347	3.7507	3.6468	3.2507	2.8906	4.0941	3.8156	3.4493	5.0032	3.5068	2.2691	3.4432	4.8606
A_32_P310503	OR2M1P	1.6422	1.9542	1.8163	1.1048	2.7103	1.7962	1.7119	1.6267	1.5464	0.7432	1.3273	1.7034	1.6122	1.8191	1.6406	1.1017	0.7196	1.5167	1.5786	1.7499	1.3905	1.1334	0.9628	1.4612
CUST_7141_PI416261804	OR2M1P	3.5431	4.8120	3.4660	3.7764	4.7071	3.6730	3.8708	5.1334	3.8250	3.7936	4.1864	1.9939	3.3122	5.0299	3.1512	3.0765	3.8734	1.8678	3.0866	5.0572	4.1938	3.0247	4.5147	2.9569
CUST_1669_PI416261804	OR2M2	2.3548	2.1603	3.2555	2.6220	3.4551	2.0673	2.4385	1.6856	3.0055	2.5252	3.1155	1.9014	2.1754	2.0797	2.7152	1.4770	3.7580	1.7714	2.0253	1.9729	2.0233	1.4960	4.0440	1.7437
CUST_1114_PI416573500	OR2M3	1.8763	2.3993	2.7504	1.6965	4.4777	2.1339	1.9553	1.8796	3.2815	1.2081	3.0793	2.1136	1.7714	2.2243	3.4000	1.4500	2.5003	1.8747	2.4180	2.5308	2.6220	1.5858	4.1042	1.6127
CUST_1151_PI416379584	OR2M3	2.4244	2.0923	2.9412	1.3801	3.9905	1.9846	2.7070	3.2959	3.4938	3.5722	3.3338	1.8496	2.7898	2.0222	3.2164	2.0543	3.5849	1.7004	2.3485	1.8564	3.5624	1.4041	4.1893	1.7121
CUST_317_PI416573500	OR2M4	1.8049	2.0108	1.7682	1.1673	1.8218	1.8609	1.9009	1.5481	1.5558	0.7432	1.4063	1.7809	1.7043	1.8567	1.7083	1.1453	2.4973	1.6723	1.7282	1.8805	1.4884	1.2355	1.8300	1.5749
CUST_326_PI416379584	OR2M4	3.7455	5.3568	2.6663	1.4148	3.6055	2.7723	4.8988	5.0403	2.2475	2.4605	4.3082	3.4356	3.3588	5.1004	1.4582	2.7795	3.3589	2.2310	4.1901	5.4984	2.9615	2.3013	4.3033	4.2562
CUST_61_PI416379584	OR2M5	1.7113	3.0131	1.4314	1.0385	1.3636	2.3326	1.8984	2.9968	1.9597	0.7432	2.4764	1.6142	2.1356	1.7745	1.6173	1.0067	2.7292	1.5262	1.5910	1.6259	2.1843	1.0929	2.0413	1.4689
CUST_324_PI416379584	OR2M7	7.1999	5.7813	5.9641	4.7445	6.5079	5.7672	7.3514	6.7473	6.1527	5.7009	5.9472	4.4895	4.9104	6.1563	4.9547	5.0174	5.0612	4.0651	6.7011	4.9763	6.5845	4.7926	6.3608	4.8213
CUST_57_PI416573500	OR2M7	2.6717	2.2253	3.3485	2.7848	4.1503	2.3331	2.9858	3.9635	3.4453	3.3219	3.3303	1.9336	2.6990	2.6333	3.1194	1.4825	3.2265	2.2098	2.6928	3.0460	2.9395	3.1956	4.6531	3.3277
A_23_P135007	OR2S2	1.7955	2.1248	1.8139	1.3888	2.3942	1.9977	2.1650	1.6690	1.7390	1.0180	1.6220	1.9662	1.8242	2.1266	1.9101	1.5961	1.2221	1.9342	2.3621	1.9784	3.9313	1.4500	1.0564	1.5329
CUST_6600_PI416261804	OR2S2	2.4159	2.2914	1.9002	1.0781	2.5926	1.6761	2.2849	2.7434	2.1364	1.1110	1.9969	2.5984	2.3367	1.7903	1.9221	1.1789	1.8545	2.4993	1.8192	1.9479	2.9448	1.0546	2.9135	1.8282
**CUST_188_PI416573500**	**OR2T1**	**2.0397**	**2.1639**	**1.7012**	**1.2858**	**1.2378**	**1.9240**	**2.2746**	**1.9069**	**1.6653**	**0.7432**	**1.7222**	**1.9745**	**1.8611**	**2.1004**	**1.6897**	**1.5403**	**1.1681**	**1.7145**	**1.8304**	**1.9897**	**1.6029**	**1.3207**	**1.2471**	**1.8195**

**CUST_193_PI416379584**	**OR2T1**	**2.8074**	**2.1011**	**3.5922**	**2.0589**	**4.7338**	**2.1411**	**3.2257**	**3.2153**	**3.9741**	**3.9094**	**3.5009**	**1.8295**	**3.0156**	**2.6169**	**3.1623**	**1.1938**	**3.5439**	**1.9261**	**2.4899**	**1.8411**	**3.6849**	**2.6875**	**5.2001**	**2.2768**
CUST_383_PI416573500	OR2T10	2.0555	2.3084	2.0267	1.7562	1.5618	2.3252	1.9028	2.5267	1.8467	2.2801	1.7864	2.1909	2.1783	2.2976	1.9889	1.8497	2.2199	1.8114	1.9065	2.1383	1.7160	1.7550	2.8054	1.5323
CUST_392_PI416379584	OR2T10	1.9664	2.2496	1.6301	1.2398	2.2833	1.9784	2.1502	1.9958	1.5471	0.7432	1.7017	1.9756	3.0625	2.0342	1.7077	1.3604	1.0954	1.7372	1.7803	2.0894	1.4808	1.2457	1.2724	1.9199
CUST_321_PI416573500	OR2T11	1.8050	1.9781	2.4225	1.6027	3.0344	1.9209	2.1978	2.5648	2.4619	0.8618	1.3490	1.8050	1.7237	1.8646	1.7400	1.1174	0.7692	1.7264	1.7413	1.8545	1.9676	1.7744	2.8436	1.6094
CUST_330_PI416379584	OR2T11	1.8152	2.3005	1.6804	1.3199	1.2563	2.0862	1.8734	1.8466	1.6326	1.2034	1.7517	2.0005	1.7046	2.0578	1.7777	1.3344	1.1035	1.7645	1.7311	2.1700	1.4799	1.3743	1.3428	1.6200
CUST_233_PI416379584	OR2T12	2.9627	3.4805	3.6749	3.6827	5.1130	3.1562	3.2363	4.3889	3.8354	4.5510	4.2733	2.0074	3.0210	4.4413	3.5085	3.5360	4.1659	1.7622	2.5494	3.2273	3.1352	2.7663	4.5574	1.7416
CUST_141_PI416573500	OR2T2	1.9220	2.2157	2.0975	1.4332	1.2925	2.1211	2.0605	1.8228	1.6951	0.7432	1.8487	1.9974	1.8242	2.1586	1.7469	1.6401	1.1436	1.7472	1.7887	2.0380	1.6276	1.5587	1.1966	1.6508
CUST_146_PI416379584	OR2T2	1.9511	3.0619	3.1779	2.7229	1.8938	4.2686	1.7372	1.5856	3.3262	0.7432	3.4385	1.7733	2.0979	2.4063	2.6522	1.1657	3.6194	1.8472	2.2555	2.6946	2.9789	3.8552	1.0682	2.5749
CUST_308_PI416573500	OR2T27	2.2113	2.0721	1.8875	1.4372	1.2625	2.1907	2.4540	1.6582	1.9689	0.7432	1.5067	1.8828	2.0110	2.0736	1.6596	1.5122	1.0589	1.8228	2.0831	1.8949	1.7287	1.6048	1.4190	1.8459
CUST_316_PI416379584	OR2T27	3.0239	2.0923	3.3303	2.7728	3.3096	1.6243	3.9417	2.5509	3.7207	3.5168	4.4844	1.6308	2.1800	1.9942	2.7900	2.6367	3.1366	1.6471	2.5082	3.2634	2.5238	3.3681	3.9036	2.3287
**CUST_226_PI416573500**	**OR2T33**	**2.6897**	**2.2074**	**2.0343**	**1.4145**	**1.3088**	**2.1703**	**3.0406**	**1.7751**	**1.7739**	**0.7432**	**1.5851**	**1.9445**	**2.4824**	**2.1281**	**1.6721**	**2.3170**	**1.0601**	**1.7639**	**2.7229**	**2.0050**	**1.6380**	**1.5824**	**1.3123**	**1.7612**
**CUST_274_PI416573500**	**OR2T33**	**4.1399**	**2.2679**	**4.2413**	**3.1739**	**4.7397**	**2.0403**	**4.0846**	**2.3683**	**4.1407**	**4.1669**	**3.9602**	**1.8795**	**3.0588**	**2.8227**	**3.8532**	**3.0465**	**3.9448**	**2.3534**	**3.9306**	**1.9941**	**3.8206**	**3.7856**	**4.4992**	**3.0643**
**CUST_282_PI416379584**	**OR2T33**	**1.8958**	**2.0828**	**1.6163**	**1.2967**	**1.9820**	**1.9889**	**2.0194**	**1.6127**	**1.9939**	**0.7432**	**1.4512**	**1.8034**	**1.7251**	**1.9280**	**1.6338**	**1.2102**	**0.7931**	**1.5983**	**1.7839**	**1.7873**	**1.5390**	**1.3253**	**2.3693**	**1.6433**
CUST_250_PI416573500	OR2T34	2.1567	3.6003	3.1457	2.4898	3.4368	2.4715	2.6286	3.3083	3.2096	2.5529	3.1023	1.7858	1.8275	3.8395	1.7815	1.2580	2.7777	2.1498	2.4369	3.1533	3.0096	1.4219	4.1699	1.6143
CUST_258_PI416379584	OR2T34	2.1667	3.5436	1.8609	1.7825	2.1928	3.0966	3.5997	2.6635	1.8127	2.7747	1.7070	2.0178	2.5531	3.5280	1.8417	1.3315	1.2592	1.8790	2.1450	2.9170	2.4820	3.1219	2.6583	2.8419
CUST_384_PI416573500	OR2T4	1.5787	1.8325	1.4340	0.9785	0.8081	1.6182	4.4337	1.4419	1.5303	0.7432	1.1977	1.5996	1.5450	1.6632	1.6327	1.0546	0.7515	1.4333	1.5431	1.6896	1.4050	0.9801	0.8661	1.4224
CUST_393_PI416379584	OR2T4	2.8822	1.9390	3.2955	1.0243	2.8778	1.7510	3.0228	2.8099	3.5191	0.7432	2.5347	1.8056	1.9485	1.7609	2.5540	1.6123	0.6954	1.6693	2.4350	2.2074	3.3312	2.1916	3.0612	2.3214
A_24_P281439	OR2T5	2.4796	2.4688	3.4460	2.1833	3.2406	2.6832	2.3000	3.8934	3.8693	2.9409	3.6255	1.5475	1.9095	2.0797	2.3717	1.5813	1.5023	1.7081	1.8713	2.8440	3.0996	2.4763	2.7902	1.6804
CUST_774_PI416261804	OR2T5	1.5435	1.9420	1.4798	1.0046	0.8879	1.5779	1.6170	1.5516	1.5185	0.7432	1.3783	1.6603	1.5248	1.7301	1.7232	1.1704	0.8774	1.4211	1.5203	1.8136	1.4249	0.9393	2.4713	1.4117
CUST_334_PI416573500	OR2T6	1.8242	2.0612	1.6724	1.1553	1.0360	2.1734	1.9538	2.5097	2.0815	0.7432	3.3449	1.7675	1.7221	1.8627	1.8914	1.9704	1.7499	1.6392	1.7303	1.9280	1.4883	1.1993	2.5261	1.6023
CUST_343_PI416379584	OR2T6	6.2234	6.1423	6.6122	6.7836	7.5495	5.2279	6.7741	6.9153	6.7591	7.1632	6.9597	4.6231	4.8931	6.4016	5.8940	5.4637	6.0354	4.2546	6.8188	6.2453	5.9893	6.9341	7.9929	4.5440
A_24_P298724	OR2T8	1.7010	2.3683	1.6063	1.2138	1.0904	1.8807	1.7761	1.6122	1.7874	0.7432	1.4262	1.8540	1.6484	1.9487	1.6996	1.2925	0.8442	1.5576	1.7173	1.9053	1.4888	1.2259	1.0999	2.1482
CUST_413_PI416261804	OR2T8	2.0485	2.1889	1.6734	1.3291	2.4094	2.1079	2.2338	2.3866	1.6569	0.7432	2.1308	1.8208	1.7720	1.9942	1.7164	1.2954	1.0449	1.6580	1.8586	2.0089	1.9654	1.3548	1.3371	1.6759
A_23_P18913	OR2V2	1.8888	2.1966	1.8298	1.1737	1.3940	1.8984	2.0456	1.7672	2.1721	0.7432	1.6993	1.9265	4.2606	1.9735	1.7667	1.3166	2.1132	1.7529	1.8007	2.1442	1.5103	1.1847	1.3574	1.7418

CUST_1633_PI416261804	OR2V2	2.6373	1.9941	3.5303	1.8892	4.1640	1.8651	3.3877	2.6930	3.0595	2.9064	2.3483	1.9492	2.1857	2.0276	2.8082	1.3685	3.1697	1.8156	2.4698	1.7759	3.6910	3.8415	4.3436	2.2809
A_23_P255389	OR2W1	1.7867	2.1197	1.6580	1.2882	1.6916	1.9537	1.8610	1.6421	1.6383	0.7432	1.6423	1.8455	1.7162	1.9368	1.6579	1.2809	0.9111	1.5916	1.6974	1.8339	1.5312	1.2847	1.1962	1.4962
CUST_6437_PI416261804	OR2W1	1.7528	2.1746	1.6425	1.3861	1.0819	2.1276	1.8065	1.7205	1.5579	0.7432	1.4906	1.8808	1.6511	2.0573	1.5633	1.3958	0.7632	1.6200	1.7084	1.9350	1.5159	1.4655	1.0991	1.4548
A_24_P333993	OR2W3	1.9963	2.5330	2.4206	1.9024	3.6997	2.5108	1.9532	1.8829	2.5255	1.7550	2.7494	2.3127	3.2014	2.3838	2.1357	1.6012	1.5491	1.9542	1.8381	3.0081	2.2108	1.9316	2.2380	1.6345
CUST_1659_PI416261804	OR2W3	4.4108	2.7296	4.9264	4.0411	5.2922	2.9219	4.5715	3.8912	5.2386	4.8528	4.3385	3.3200	3.2164	2.1053	5.0798	3.8750	4.4855	4.3719	4.1109	2.7689	4.5689	4.4712	4.9074	3.6903
CUST_752_PI416573500	OR2W5	5.9412	4.3690	6.8530	4.7581	6.7634	4.5283	5.5377	4.9574	6.8002	5.3195	5.5229	5.0463	4.1484	5.3607	6.9478	4.1815	5.6801	4.0300	5.1250	4.5115	6.3014	5.3817	5.4583	4.2652
CUST_778_PI416379584	OR2W5	2.1175	2.0373	2.6319	1.2309	2.6280	1.9680	2.3409	1.5509	2.0510	1.1847	2.3852	1.7809	1.9729	1.8760	1.6764	2.9648	2.4490	1.8130	1.9843	1.8982	1.6809	1.3472	2.0554	1.8160
CUST_392_PI416573500	OR2Y1	1.9029	1.8352	2.6808	1.7522	1.4039	1.5994	2.1682	1.4472	2.6143	2.0607	1.7211	1.5745	1.6900	1.6310	2.0373	1.0523	1.8411	1.4747	1.7589	1.6926	2.6110	0.9511	3.1139	1.9487
CUST_401_PI416379584	OR2Y1	3.8048	2.4574	3.3029	2.6760	3.1469	1.7657	3.4793	3.5483	3.2544	3.2565	4.4756	1.5581	2.2197	3.8462	1.9861	2.3567	0.6745	1.8255	2.9619	2.7586	3.0545	1.7534	3.7610	4.3802
CUST_1020_PI416573500	OR2Z1	6.1566	4.8719	6.8540	6.1793	6.8174	5.5704	5.2585	6.1896	6.8290	6.8149	5.7229	6.1478	4.8317	5.9820	6.9696	5.4821	5.7851	6.1474	6.0316	4.5378	6.4395	6.2139	5.8954	5.6616
CUST_1052_PI416379584	OR2Z1	2.0828	2.2037	2.7765	0.9234	0.8478	4.1383	2.4969	1.7796	2.4012	0.7432	1.6861	1.7697	1.8916	1.8286	1.6991	1.3020	3.3260	1.5910	1.9149	1.9982	3.3668	0.7550	1.9249	1.9633
A_23_P50031	OR3A1	2.0501	2.1624	1.5640	1.2069	0.8956	1.9742	2.2682	1.8119	1.6386	1.1212	1.7155	1.8880	1.9613	1.9731	1.6702	1.2038	0.9822	1.7852	2.1181	1.9521	1.5178	1.2647	1.2548	1.8042
CUST_5004_PI416261804	OR3A1	8.7383	7.1093	7.7320	6.9569	7.9932	6.7705	8.7300	7.3733	7.9958	7.6597	7.3550	6.3765	6.5257	7.6338	7.7570	6.0714	7.2659	6.5411	8.6602	6.8188	7.7164	7.0831	8.6633	6.4448
A_23_P55504	OR3A2	2.0484	2.3402	1.8783	1.3914	2.2332	2.2922	2.3968	1.9722	1.7070	2.0954	1.4001	2.0475	2.8010	4.1207	1.7816	2.7910	3.3849	2.0429	2.0051	2.4310	1.6798	1.6267	2.5466	1.9496
CUST_5404_PI416261804	OR3A2	4.4227	2.1508	4.9175	4.8497	4.7919	4.3742	4.4083	3.8495	4.6863	4.5835	3.4481	2.9754	3.6055	4.0676	4.8010	3.2901	4.0211	3.7035	4.3620	2.7121	4.4721	2.6043	4.8165	3.6134
A_23_P107483	OR3A3	2.9552	2.1196	4.2084	2.2343	5.8559	3.2742	3.7460	1.8978	4.4490	3.6253	3.6609	2.0434	2.4350	2.2660	3.5848	1.4764	3.9986	2.0567	3.4430	1.8910	4.0432	1.6066	5.4693	2.4720
CUST_2232_PI416261804	OR3A3	6.6749	5.5315	6.8141	6.1209	6.8599	4.8785	7.2895	6.4602	6.9589	6.5562	5.8575	5.5898	5.2292	6.3254	7.3456	5.2451	5.8722	5.4207	6.9051	5.4838	6.4901	6.2349	6.5366	4.7827
A_23_P139099	OR4A15	3.6896	2.4384	4.1941	3.6981	5.6001	2.0719	3.7131	4.4192	4.7630	4.6008	4.5023	2.0173	2.8166	3.6061	3.6491	3.1156	4.2930	1.9648	2.9974	2.8427	3.8651	2.8992	5.5962	2.3466
CUST_1565_PI416261804	OR4A15	2.0179	2.1532	1.7839	1.5228	1.3646	2.1718	2.1494	1.7576	2.2107	0.7432	1.7692	1.9460	1.8856	2.1505	1.7259	1.6461	1.1111	1.7672	1.9151	1.9350	1.6663	1.5858	1.2235	1.6494
CUST_139_PI416573500	OR4A16	1.7073	2.0855	1.9569	1.2742	2.3468	1.8360	1.8310	1.7618	1.9883	2.0445	1.5352	1.8512	1.6956	1.9555	1.8568	1.2404	1.0290	1.6633	1.6317	1.8893	1.4431	1.1848	2.3239	1.5547
CUST_144_PI416379584	OR4A16	2.3215	2.4350	1.5352	2.4387	0.9694	1.9483	2.4840	1.7448	1.6742	0.9066	1.6463	1.8828	1.7655	2.2408	2.1893	1.2038	0.9658	1.6945	1.8778	2.2781	1.7835	2.7964	2.6283	1.8582

CUST_375_PI416573500	OR4A47	2.5066	2.1760	3.6878	3.5493	4.8107	1.9459	2.7606	4.0098	4.1515	4.8077	3.9988	1.7573	1.9708	2.1458	2.6484	3.0230	4.1002	1.8406	2.3722	2.6014	4.0391	3.9470	3.7314	2.4990
CUST_384_PI416379584	OR4A47	2.9863	2.2837	2.3354	1.3477	1.7785	2.1378	2.7489	2.5439	1.6016	2.7731	1.6091	1.8463	1.9422	3.2668	1.5958	1.6392	1.1206	2.0236	2.1071	2.5439	2.5129	3.2124	1.4663	3.6624
CUST_224_PI416573500	OR4A5	2.1038	2.1841	1.8079	1.4795	1.9945	2.1371	2.2988	1.7955	1.8102	0.7432	2.5274	1.9485	1.9506	2.1583	1.7521	1.6711	2.1533	1.7444	1.9677	1.9634	1.8387	1.5206	2.2712	1.7935
CUST_230_PI416379584	OR4A5	1.6680	1.9575	1.4668	1.0842	0.8567	1.8627	1.7356	1.5106	1.4233	0.7432	1.1850	1.6530	1.5510	1.7334	1.5513	1.0824	0.6745	1.4625	1.6141	1.7602	1.3900	1.1464	0.9437	1.6218
CUST_442_PI416573500	OR4B1	1.6236	1.9894	1.5705	1.1705	2.1233	1.7734	1.6222	1.6243	1.8283	0.7432	1.4400	1.7236	1.5441	1.8805	1.7655	1.1912	0.8502	1.4635	1.5544	1.8185	2.6785	1.1477	1.8132	1.4392
CUST_451_PI416379584	OR4B1	2.2546	3.0580	2.3938	1.9160	0.9370	3.5060	2.3683	3.8401	1.4179	1.3034	1.3333	4.2514	1.7701	2.7792	1.4180	1.2613	0.8567	2.0199	2.1998	3.1194	2.9438	1.0206	1.3492	2.9609
CUST_158_PI416573500	OR4C11	2.1523	2.3722	2.1352	1.4054	2.6346	6.0379	2.2108	1.5988	1.9458	1.4866	1.8314	2.0400	1.9009	2.0829	1.8675	1.2834	0.8526	1.8728	1.9522	2.0253	2.2218	1.5229	3.7465	3.1678
CUST_163_PI416379584	OR4C11	2.1359	3.3553	1.8028	2.0383	0.9597	2.6119	2.2304	1.7558	1.7229	0.7432	1.5935	1.8786	1.7408	2.9784	1.5346	2.8162	0.9788	2.4279	1.9428	3.5118	1.4452	2.0121	1.8673	2.8075
CUST_264_PI416573500	OR4C12	1.7004	2.0964	1.6822	2.0559	1.9120	1.9152	1.8645	1.9477	1.5440	0.7432	3.6094	1.8747	1.6623	2.6350	1.6390	1.2834	0.8502	1.9814	1.6606	1.9212	1.5138	1.2695	1.6214	1.4687
CUST_272_PI416379584	OR4C12	5.9078	2.7630	3.7368	5.9370	1.9449	4.5415	3.6813	2.1789	4.7796	4.4727	1.4362	4.9141	2.5217	3.3417	4.1964	6.4402	0.6745	4.6205	4.5299	1.9373	5.3426	1.1121	2.1747	5.2164
CUST_276_PI416573500	OR4C13	1.7615	2.1789	1.6264	1.2998	0.9501	2.0441	1.8199	1.8081	1.6167	0.7432	1.5497	1.7710	1.6347	1.9748	1.6497	1.2363	0.8522	1.5693	1.7562	1.9752	1.4562	1.3452	1.8993	1.5070
CUST_284_PI416379584	OR4C13	1.9395	2.1705	1.4696	3.7815	0.9293	5.2096	2.1905	1.6946	1.7824	0.7432	1.3750	1.6732	1.6977	2.0561	1.4059	1.1738	0.7636	1.5312	1.7654	3.3204	2.3605	1.0468	1.1246	2.5681
A_23_P52914	OR4C15	3.5658	2.0125	4.1358	2.9479	4.5108	2.7006	4.8439	4.1824	4.5104	4.3123	3.9081	1.5981	2.2779	2.1291	4.3478	4.1742	3.4756	1.6978	3.0600	1.7672	3.8338	1.6738	3.5318	3.0590
CUST_1556_PI416261804	OR4C15	1.7450	2.0474	1.6355	1.3483	1.1417	2.0293	1.7817	1.6247	1.5215	0.9009	1.4617	1.8552	1.6621	1.9776	1.6512	1.3732	0.8459	1.5958	1.7000	1.8762	1.5089	1.3620	1.0243	1.4665
CUST_351_PI416573500	OR4C16	1.8965	2.3470	1.6860	1.2954	1.0478	2.0444	1.9382	1.6746	1.5895	1.2564	1.6865	1.9367	1.7742	2.0143	1.7749	1.3167	0.9599	1.7033	1.7382	3.4738	1.6351	1.3193	1.9243	3.4985
CUST_360_PI416379584	OR4C16	1.8575	3.5520	2.3848	1.2940	0.9349	1.9910	2.2298	1.9259	1.6021	0.7432	1.8040	1.7693	2.5799	2.0263	1.6664	1.2902	0.9864	1.9282	1.6991	2.6507	3.1140	1.2970	1.6304	3.0191
A_23_P2077	OR4C3	1.8628	2.2347	2.0391	1.2268	2.0802	1.9115	1.9189	1.8895	1.6141	1.7526	1.8613	1.9762	1.7759	2.0569	1.7952	1.3273	1.1794	1.7734	1.7697	2.0500	1.5134	1.2327	1.1890	1.6768
CUST_2024_PI416261804	OR4C3	1.6019	1.8874	1.5947	1.0336	0.8310	1.6680	1.6617	1.4768	1.9569	0.7432	1.2379	1.6389	1.5544	1.7433	1.6745	1.1065	0.7665	1.4619	1.6686	1.7120	1.4374	1.0438	0.8567	1.4243
CUST_30_PI416573500	OR4C45	1.8219	1.9101	2.0349	1.0821	1.9532	1.7624	1.8177	1.4810	1.9481	0.7432	1.2191	1.6623	1.6361	1.7834	1.6480	2.2847	0.7300	1.5434	1.6204	1.7045	1.9846	1.1380	0.9276	1.4721
CUST_32_PI416379584	OR4C45	1.7985	2.2083	1.5945	1.2249	1.1394	3.3480	1.8863	1.7759	1.5184	2.3466	1.5507	1.8772	1.9043	1.9777	1.6659	1.2474	0.8831	1.8947	1.6873	2.0672	1.4164	1.3020	1.2417	1.5675
A_24_P281403	OR4C46	2.0114	2.1561	1.5835	2.9768	1.0456	2.5497	2.1206	2.9784	1.8266	2.8668	1.6131	1.8125	1.8862	1.9078	1.6798	1.2421	1.0344	1.6574	1.9569	2.0070	1.4785	1.7386	1.2585	1.9806
CUST_179_PI416261804	OR4C46	1.8745	1.9288	1.4677	1.0657	0.9236	2.6821	2.1352	1.5274	2.0526	0.7432	2.8315	1.6432	1.6873	1.7370	1.4887	1.2210	0.7496	1.4895	1.7452	1.7583	2.1474	1.0647	0.9396	2.0260
CUST_322_PI416573500	OR4C6	1.7063	1.9403	1.4438	1.0759	0.7772	1.8206	1.8660	1.5460	1.6932	0.7432	1.1838	1.6377	1.6209	1.7514	1.6175	1.4055	0.6745	1.5074	1.6327	1.7169	1.5540	1.1196	0.9617	1.5233
CUST_331_PI416379584	OR4C6	2.1838	3.4613	1.5591	1.2195	1.1532	1.7452	2.4420	1.8081	1.8062	1.7824	2.4745	1.8049	2.2195	1.9135	1.6450	1.3222	1.2473	2.0879	1.9524	3.1920	2.8360	0.9833	2.0603	3.4441
CUST_510_PI416573500	OR4D1	1.7839	2.4092	1.7337	1.2673	1.2161	2.1386	1.8110	1.7873	1.6536	0.7432	1.6953	2.1265	1.7664	2.0996	1.9153	1.3061	1.1820	1.8887	1.7562	2.3412	1.7509	1.2730	1.3699	1.6509

CUST_521_PI416379584	OR4D1	2.2866	2.3470	2.6638	1.2384	0.9139	1.9142	2.5699	3.7117	2.0407	0.7432	1.9515	2.1807	1.8775	2.0051	1.7147	1.4059	1.1270	1.8796	2.3193	3.8324	3.1928	1.3879	1.3205	2.7126
CUST_135_PI416573500	OR4D10	2.1342	1.9735	2.2835	0.8993	0.9901	1.5248	2.5119	1.6548	3.7465	1.3509	2.0618	1.6469	1.7900	1.7093	3.3555	1.2526	1.8502	1.4989	1.9377	1.9150	3.2941	0.7688	1.0852	1.9846
CUST_140_PI416379584	OR4D10	4.0579	3.7088	5.2840	4.8971	5.2858	3.9585	3.9236	4.6654	5.4893	5.8030	4.6753	3.3063	3.9933	2.9150	5.2041	3.9672	4.1655	3.5463	4.0132	3.0661	5.6776	5.3930	5.3145	3.8926
CUST_324_PI416573500	OR4D11	1.7079	1.8540	3.1188	1.9549	3.3216	1.5700	1.9645	3.6674	3.5643	2.2586	1.7341	1.5481	1.6442	1.6337	1.6272	1.0312	0.7338	1.4479	1.8652	2.2874	2.3170	3.2282	2.6233	1.5157
CUST_333_PI416379584	OR4D11	2.2702	2.0660	2.6033	1.1583	3.0317	2.1136	2.3821	1.6333	2.7397	2.4785	2.3325	1.7586	1.9500	1.8564	1.7451	1.2130	1.6898	1.6616	1.9594	1.9390	2.1002	1.1909	2.4497	1.7872
A_23_P77797	OR4D2	7.2695	7.1110	8.1811	7.2250	8.0300	7.0444	7.0913	7.4021	8.1366	7.5584	6.7809	7.2632	5.8575	7.3279	8.3460	6.2166	6.8718	6.8438	6.7622	6.7339	7.8250	7.5782	7.4371	6.5940
CUST_2198_PI416261804	OR4D2	2.9585	1.8784	3.6832	3.3253	3.6218	1.9395	3.3730	3.3004	3.4958	3.6053	3.0757	1.5581	2.0982	3.0825	2.8594	3.1291	2.9941	2.0303	2.8070	1.5917	3.1196	4.4055	4.3178	4.0194
A_23_P75663	OR4D5	1.7589	2.0078	1.9041	1.1047	1.8050	1.7823	1.9392	1.5905	2.3389	0.7432	1.4183	1.7940	1.7008	1.8717	1.6611	1.2467	0.9025	1.5555	1.6731	1.8856	1.9072	1.1140	1.0666	1.5637
CUST_1038_PI416261804	OR4D5	2.0278	2.3831	2.4188	2.9960	2.9839	2.1787	2.1490	1.8422	2.4480	2.7203	2.2431	2.0993	1.8899	2.1598	1.9193	1.3239	1.4613	1.9919	1.9479	2.3152	1.9939	1.3543	2.6112	1.8797
CUST_233_PI416573500	OR4D6	4.0590	2.2302	4.4892	2.1986	3.4166	4.0379	4.7179	3.4649	5.0085	3.7894	3.9859	1.7483	2.4828	2.2545	4.6757	3.1226	3.5582	2.5352	2.9289	3.9325	3.5999	2.6781	4.3510	3.9913
CUST_240_PI416379584	OR4D6	3.5422	4.8226	4.4860	4.3312	4.5933	2.5544	4.9060	4.3675	4.4808	4.5745	3.9275	3.5622	3.7212	3.3884	4.7084	3.6318	4.3444	2.4034	3.6818	3.2552	4.1373	4.6393	4.2271	3.6805
CUST_440_PI416573500	OR4D9	1.8800	2.2516	2.2769	1.1893	4.1866	1.9161	2.0219	3.4086	2.1481	0.7432	1.6258	1.9481	1.8017	2.0215	1.7426	1.3173	1.0929	1.7594	1.7383	2.0736	1.7518	1.2059	2.2677	1.7309
CUST_449_PI416379584	OR4D9	2.4156	3.5194	2.3487	2.4381	0.8519	3.4473	2.8763	1.7571	1.9701	1.1753	1.4647	3.3742	1.8076	2.5034	2.1801	3.5773	1.9297	2.6113	2.1482	3.1540	3.9923	1.9615	0.9791	4.8816
CUST_505_PI416573500	OR4E2	1.9886	6.8701	1.7901	1.5252	1.3947	4.1841	2.1481	1.7602	2.2433	0.7432	1.6532	1.9611	1.8521	2.1339	2.3070	1.6735	1.0969	1.8746	1.8925	1.9907	1.6375	1.5498	1.6624	1.6056
CUST_516_PI416379584	OR4E2	2.1368	2.3121	1.7736	1.1889	0.8616	1.8886	2.3569	1.6641	1.6911	2.6212	1.6655	3.2455	1.8627	1.9495	1.6877	1.3013	1.0708	2.7515	1.9715	2.4936	2.2880	2.8731	1.2965	1.9707
CUST_291_PI416573500	OR4F15	1.6681	1.9722	1.9993	1.1585	0.9913	1.8504	1.7132	1.5512	1.6190	2.6853	1.3363	1.7657	1.5862	1.8445	1.6212	1.2355	1.8741	1.4979	1.6223	1.8179	1.4341	1.1884	0.9420	1.4316
CUST_299_PI416379584	OR4F15	2.0645	2.1877	1.6901	1.2847	1.0911	1.9769	2.2988	1.9233	1.6157	1.0273	1.7115	2.4597	1.8426	2.0863	1.6246	1.5352	1.4154	1.6766	1.9302	2.6466	1.5210	1.3366	1.2586	1.8216
CUST_29_PI416379584	OR4F21	4.4749	4.3703	2.8175	2.9901	1.0291	4.0350	4.1293	4.1274	2.9807	3.1584	2.4778	3.5500	2.2373	1.8875	2.0334	2.2941	0.8093	2.9243	3.3978	4.5005	3.1065	1.2217	2.2029	4.9931
CUST_27_PI416573500	OR4F3	5.2735	5.3321	5.5655	5.2250	6.0498	3.8924	6.0233	5.6226	5.6347	5.3679	5.0771	3.7429	4.3416	5.8100	6.0356	4.0090	5.1670	4.9052	5.0485	5.3709	5.2201	5.7010	5.5279	4.0298
CUST_520_PI416379584	OR4F3	3.1852	3.4376	2.8802	2.4955	0.7542	3.5668	3.3328	3.2105	2.7255	0.7432	1.0638	2.3550	2.9905	3.0869	1.6892	1.7445	2.4453	3.3044	2.3337	3.4907	3.5366	2.7203	1.8150	3.0681
**A_24_P58501**	**OR4F4**	**1.6706**	**2.3514**	**1.6230**	**2.7776**	**1.9975**	**1.9347**	**1.6877**	**3.1833**	**2.0147**	**2.8359**	**1.6268**	**1.8844**	**1.5868**	**2.7667**	**1.7754**	**1.2993**	**0.9504**	**1.5775**	**1.6171**	**2.1013**	**1.4886**	**3.0285**	**1.1405**	**1.4569**
**CUST_1808_PI416261804**	**OR4F4**	**1.7096**	**3.1533**	**2.4440**	**0.9646**	**0.9131**	**1.6183**	**1.9872**	**2.1508**	**1.4167**	**0.7432**	**1.4109**	**1.5916**	**1.6282**	**1.7524**	**1.5248**	**1.2519**	**0.9280**	**1.7831**	**1.5709**	**1.8093**	**2.2363**	**0.9634**	**0.9133**	**1.5248**
CUST_964_PI416261804	OR4F5	3.6114	2.0964	2.5003	1.1365	1.1976	1.8245	3.1378	2.2267	2.3755	0.7432	1.6007	1.6607	2.0177	1.8814	1.4626	1.6133	1.1475	1.7427	2.3498	2.6691	3.3548	1.5910	1.1198	2.9329

CUST_1150_PI416573500	OR4F6	1.6559	2.0829	1.4940	1.2547	0.8184	1.8831	1.7170	1.7555	1.4643	0.7432	1.4250	1.7752	1.5841	1.8540	1.6326	1.1229	0.7975	1.5303	1.5973	1.8519	1.3911	1.1399	1.0261	1.4728
CUST_1188_PI416379584	OR4F6	1.9299	1.8613	1.4208	0.9331	0.7206	1.6581	2.2440	1.5693	1.7008	0.7432	1.2125	1.4708	1.6862	4.9632	1.4061	1.2911	0.6851	1.4270	1.7290	1.6328	2.3152	0.9334	1.0739	2.7892
A_23_P2973	OR4K1	1.6063	1.8937	1.4499	1.0636	2.1505	1.7128	1.6692	1.5501	1.5833	0.7432	1.2906	1.6618	1.5608	1.7790	1.6389	1.0691	0.7770	1.4700	1.5480	1.6887	1.3955	1.0636	0.9265	1.4412
CUST_1901_PI416261804	OR4K1	3.7555	2.2710	3.7414	5.0915	3.1501	3.3786	3.8575	1.8992	4.2505	4.2002	3.0082	2.1444	5.6480	1.6355	3.6441	6.5313	3.1362	4.1901	4.6433	1.5394	4.8790	2.4955	3.3573	3.6466
CUST_172_PI416573500	OR4K13	1.7685	2.4131	1.8511	1.4868	2.0663	2.3336	1.6307	1.7955	1.8186	0.9748	2.4733	2.2269	1.6863	3.6467	1.9644	1.8188	1.2056	3.0992	1.7290	3.7880	1.6590	2.5666	1.7098	1.5583
CUST_177_PI416379584	OR4K13	1.8603	2.4533	2.4213	1.3905	1.1418	2.1992	1.8953	1.9315	1.6322	0.8404	1.7714	2.0245	1.7167	2.0930	1.7515	1.3206	1.0311	1.7523	1.7290	2.2241	1.5767	1.4949	1.3122	1.6374
CUST_168_PI416573500	OR4K14	1.8368	2.3370	1.8785	1.3096	1.1819	1.9948	1.9132	1.7810	1.8453	0.7432	1.8169	2.0590	1.7607	2.0337	2.2387	1.3717	1.3007	1.7924	1.7515	2.1998	1.6195	1.3232	1.3449	1.6648
CUST_173_PI416379584	OR4K14	2.5010	3.0323	2.8790	1.9908	3.5144	3.7665	2.9543	2.9246	2.6132	2.7099	2.9792	2.3819	1.6902	3.0909	1.7304	1.6253	1.1606	2.3444	1.9227	2.1117	3.1092	2.7271	2.8101	2.1529
CUST_342_PI416573500	OR4K15	1.9052	2.1676	1.8017	1.5232	1.3197	2.1996	1.9812	1.7603	1.6793	0.7432	1.6372	1.9428	1.8023	2.1457	1.6950	1.6078	1.0183	1.7448	1.8295	1.9315	1.6299	1.5941	1.1923	1.7110
CUST_351_PI416379584	OR4K15	1.7203	2.2648	1.5061	1.1209	0.8486	3.2815	1.8585	1.8811	1.4563	0.7432	1.5193	1.8459	1.6576	2.8321	1.6263	1.1718	0.8847	1.6119	1.8819	2.0211	1.4470	1.1188	1.0927	1.5881
A_23_P54088	OR4K17	3.0211	2.3605	1.6673	1.3565	1.6039	2.1937	2.0988	1.8473	1.7345	0.7688	1.7098	1.9631	1.9137	2.8164	1.7240	1.2909	1.0108	1.7687	1.8280	2.1618	2.5040	1.4763	1.3553	2.8848
CUST_1168_PI416261804	OR4K17	2.0443	1.8791	4.0857	1.0378	2.4100	1.6531	2.3908	1.5236	3.7992	2.8203	2.3904	1.6076	1.7684	1.7012	3.2039	1.2649	1.1774	1.5005	1.9755	1.7438	2.9855	0.9995	3.2983	1.7199
CUST_352_PI416573500	OR4K2	4.2972	3.8573	4.5657	4.0597	5.1802	2.8006	3.8983	3.1230	4.5693	4.6444	3.5360	4.6100	2.6619	3.8670	4.8106	2.2591	3.7257	2.6290	4.3294	3.0940	4.2093	4.4143	4.6273	3.7596
CUST_361_PI416379584	OR4K2	1.7918	2.0341	1.4530	1.9328	0.8764	1.8922	1.9509	1.8522	1.4282	1.7401	1.2630	1.6529	1.6593	2.0553	1.5161	1.0483	0.7112	1.9006	3.7537	2.6848	1.8590	1.1832	1.0548	1.8035
CUST_338_PI416573500	OR4K5	1.9757	1.8676	3.1634	1.0849	3.1122	1.7276	2.2509	3.3496	2.4907	3.0523	1.1844	1.6350	1.7515	1.7677	1.7190	1.0176	0.6745	1.5461	1.8727	1.6596	3.5595	2.3636	2.5807	2.8834
CUST_347_PI416379584	OR4K5	1.9878	2.2898	1.6458	1.2431	1.2830	2.8655	2.1579	2.0301	1.6121	1.2595	1.7092	2.0032	1.8804	2.0543	1.7075	1.3843	1.0956	1.7434	1.8062	2.1283	1.5055	1.2469	1.2830	1.8234
CUST_175_PI416573500	OR4L1	1.9032	1.8418	1.4529	0.9120	0.8076	1.5778	2.2226	1.5404	1.5047	0.7432	1.2633	1.5438	1.7396	1.6639	1.4773	1.0867	0.8245	1.5249	1.7311	1.6534	2.6202	0.9408	3.8833	1.7189
CUST_180_PI416379584	OR4L1	2.1885	2.2426	1.7985	1.3425	1.2183	2.5468	2.4876	3.2233	1.8381	0.7432	1.6384	1.8552	1.8508	2.8383	1.5943	1.6150	1.0244	1.8197	1.9991	2.0587	1.8221	1.3598	1.9433	1.8045
CUST_252_PI416573500	OR4M1	2.1478	2.2081	3.0312	1.5278	3.5265	2.1438	2.3677	2.5541	3.1926	1.0360	2.9752	1.9683	1.9432	2.1736	2.5273	1.7235	2.9120	1.7518	2.0342	2.0901	2.0160	1.5399	3.6661	1.7766
CUST_260_PI416379584	OR4M1	2.7004	2.1958	2.4616	1.1966	0.8516	1.9183	3.0712	1.6854	1.9179	2.0202	1.5792	1.7267	2.0353	1.9082	1.5678	1.2451	0.9801	2.4903	2.3546	1.9555	3.0927	1.2446	1.6744	2.4588
CUST_329_PI416573500	OR4M2	1.9955	2.2087	2.9707	1.6202	2.7291	2.2904	2.0874	1.7999	2.6999	1.7871	3.0829	2.0505	1.8906	2.2280	2.3368	1.7255	2.1621	1.8176	1.8924	2.0084	2.1463	1.7040	2.3155	1.6375
CUST_338_PI416379584	OR4M2	2.2090	2.5702	1.6833	1.0808	0.8975	2.7445	2.5481	1.7602	1.5932	0.7432	1.5841	2.5889	1.7422	1.7961	1.4986	1.2681	0.9661	2.0013	2.1233	1.8732	2.8254	1.0223	1.1258	2.9566
CUST_656_PI417507815	OR4M2	1.7461	1.7802	2.9350	1.0051	1.8385	1.7733	1.9187	1.3951	3.2425	0.7432	1.5701	1.5858	1.6591	1.6377	3.4059	1.4135	1.4109	1.6088	1.6673	1.6257	2.1222	1.0948	2.0793	1.5410
CUST_394_PI416573500	OR4N2	1.7400	1.7204	1.3926	2.5617	2.0229	2.3173	1.9760	1.9414	1.8963	1.5601	2.3542	1.4899	1.6752	1.5873	1.5630	0.9308	0.7188	1.4647	1.8230	1.5328	1.9908	0.9607	1.8315	1.5231

CUST_403_PI416379584	OR4N2	2.9246	2.6464	2.0405	4.6765	3.3494	2.1057	3.2476	4.0027	2.2658	4.0926	3.3243	2.0459	1.9442	3.1452	3.0098	3.1629	2.7250	1.7785	2.8477	2.2848	1.9553	2.1912	3.3747	3.2346
CUST_10_PI416379584	OR4N3P	2.3066	2.3201	1.7361	3.6005	1.0845	2.1222	2.4938	1.6937	1.7647	3.9117	1.7617	1.7450	2.1218	2.3512	1.5478	2.2640	0.7818	2.3507	2.1587	3.3025	1.4858	3.4596	2.2896	3.3178
CUST_8_PI416573500	OR4N3P	1.6456	1.9905	1.5723	1.9950	1.8205	1.7995	1.6570	1.5423	1.5195	1.8756	1.3470	1.8004	1.5764	1.8596	1.7549	1.2082	1.8705	1.5206	1.6018	1.8565	1.4634	1.1662	0.9610	1.4518
A_23_P336176	OR4N4	1.9103	2.3790	1.7627	1.5271	1.1355	2.2747	1.9488	2.0225	1.6869	3.2218	1.7362	1.9479	1.7196	2.1282	1.7676	1.3725	0.9166	1.6889	1.7257	2.1150	1.5656	1.5747	2.0129	1.6110
A_23_P88643	OR4N4	2.0621	2.1740	2.2462	3.7282	1.3169	2.2520	2.3823	3.1767	2.2143	3.1585	2.7242	1.7732	1.7921	1.9691	1.6961	1.5451	1.0711	1.6357	1.9995	2.4568	2.5487	1.1757	1.0358	2.3779
A_24_P210611	OR4N4	1.7363	2.0238	1.6012	1.2145	0.7459	1.8786	1.8121	1.5445	1.5749	0.7432	1.4225	1.7871	1.6960	1.8716	1.6321	1.1915	1.2695	1.5633	1.6566	1.7433	1.5135	1.2374	1.1009	1.4849
CUST_331_PI416573500	OR4N5	1.5851	1.9032	1.4879	1.0678	1.4487	1.6849	1.6343	1.5396	1.5632	0.7432	1.3245	1.6865	1.5621	1.8093	1.6968	1.1104	0.8632	1.5575	1.5409	1.7039	1.4062	1.0521	0.9163	1.4294
CUST_340_PI416379584	OR4N5	1.6679	2.0111	1.5471	1.1404	2.3079	1.7831	1.7703	1.6646	1.6979	0.7432	1.4492	1.7567	1.6399	1.8961	1.6798	1.2617	0.9484	1.6557	1.6078	1.8347	1.9386	1.1808	0.9958	1.4651
CUST_271_PI416573500	OR4P4	3.7846	2.0561	4.4630	3.0299	1.4009	3.5139	4.2960	2.7080	4.3850	3.2668	1.6732	4.3222	2.4989	1.8257	4.1048	2.3459	2.6712	4.0894	3.8903	3.0506	3.8010	1.4765	3.4764	4.5573
CUST_279_PI416379584	OR4P4	2.0989	2.9729	1.6659	1.1856	0.9586	1.9799	2.3176	2.0829	1.5405	0.7432	1.7180	2.0170	1.8551	2.0689	1.6656	1.4638	1.0768	1.7139	2.4002	2.1836	2.2985	1.1535	1.2412	1.9248
CUST_364_PI416573500	OR4Q3	1.6138	1.9301	1.5016	1.0920	0.7870	1.7197	1.6692	1.5608	1.5206	0.7432	1.3466	1.7184	1.5957	1.8409	1.7025	1.1255	1.5863	1.5179	1.5558	1.7251	1.5955	1.0877	0.9522	1.7948
CUST_373_PI416379584	OR4Q3	1.9870	3.4776	1.7634	1.1696	0.7382	2.3206	2.2554	2.6602	1.4819	0.7432	1.4654	1.6702	1.7733	1.7915	1.4809	1.2033	0.8516	1.4962	1.8381	1.7497	1.9730	1.0902	1.1170	1.9555
A_23_P13479	OR4S1	1.7595	1.9390	1.9714	1.1959	3.1587	1.9255	2.2019	1.4863	1.8039	0.7432	2.5306	1.7838	2.1893	1.8675	1.6188	1.1661	2.4540	1.6168	1.7064	1.7760	1.5074	1.2764	2.2773	1.5880
CUST_2292_PI416261804	OR4S1	2.4034	3.3422	2.5814	2.0722	0.9963	1.6522	2.6838	1.5542	2.1824	0.7432	2.3504	1.6828	1.9267	1.7315	1.5374	2.3768	0.9748	1.6284	2.4258	2.1107	3.4576	0.9687	1.1787	3.6487
CUST_253_PI416573500	OR4S2	2.6791	2.0445	3.5406	0.8971	2.2147	2.0194	2.9669	1.9891	3.0718	2.4435	2.0123	1.4763	1.7131	1.5484	1.4971	1.1305	0.6745	2.1448	2.0730	1.6761	4.1953	2.0610	2.0706	2.3958
CUST_261_PI416379584	OR4S2	1.9867	1.8796	1.6121	0.8600	2.3859	2.1741	2.3439	1.5767	1.5578	0.7432	1.3649	1.5110	1.9851	1.6728	1.3900	1.1420	0.8986	1.5169	1.8114	1.7790	2.7888	0.7432	1.5496	1.8225
CUST_481_PI416573500	OR4X1	2.9357	2.0136	3.7311	2.4434	4.3594	1.7183	3.1149	3.5091	3.8562	2.9646	3.2281	1.5579	2.8706	3.2859	3.2702	2.7319	3.1772	1.7635	3.4358	2.3651	3.9575	1.1191	3.8617	1.8837
CUST_491_PI416379584	OR4X1	3.6806	3.7662	4.0741	2.0670	1.8901	1.7373	2.7514	3.0875	3.7909	1.7275	2.3738	3.2250	1.7787	3.7860	3.6554	1.0810	0.7105	2.4569	2.3970	2.6062	3.3392	2.3604	1.7102	3.6938
A_24_P349274	OR4X2	2.7108	1.9209	4.1507	1.5106	5.2839	1.7861	3.0831	3.5173	3.1250	2.4096	3.2641	1.6765	2.2514	2.6313	4.0990	1.2955	2.8407	1.8588	3.6843	1.6189	3.2657	1.1255	4.1316	2.2687
CUST_1743_PI416261804	OR4X2	1.8522	2.7048	2.2626	0.8272	0.7176	1.8142	2.2292	1.4433	1.3607	0.9334	1.1424	1.4157	1.6459	1.4748	1.3819	0.9746	0.7300	2.1718	1.9948	2.8049	2.4478	0.9818	0.8297	3.6503
CUST_273_PI416573500	OR51A2	1.8833	2.2829	1.7175	1.2402	1.2795	2.0315	1.9995	1.8045	1.6864	0.7688	1.6919	2.0074	1.8006	2.0773	1.8211	1.3193	1.2041	1.8466	1.8089	2.2180	1.9560	1.2680	2.4554	1.7482
CUST_454_PI416379584	OR51A2	1.7240	2.1701	1.5660	1.2016	1.0377	1.9576	1.7626	1.7728	4.1244	0.7432	1.5562	1.7956	1.6058	1.9139	1.6601	1.2168	0.8769	1.7689	1.6510	2.0058	1.4167	1.2659	1.1635	1.5062
**CUST_281_PI416379584**	**OR51A4**	**1.9207**	**2.9541**	**1.6066**	**1.2398**	**1.1497**	**2.0903**	**2.0408**	**2.0121**	**1.5236**	**0.8293**	**1.6470**	**1.9712**	**1.7708**	**2.0133**	**1.6736**	**1.2950**	**0.9949**	**4.2655**	**1.7741**	**2.1243**	**1.5001**	**1.2737**	**2.3520**	**1.7773**
**A_23_P13455**	**OR51A7**	**3.1660**	**2.2825**	**2.3604**	**3.5578**	**3.9145**	**1.6182**	**3.4189**	**3.8667**	**3.5667**	**3.3574**	**3.5442**	**1.7117**	**1.8885**	**2.8917**	**1.6188**	**4.3921**	**2.9496**	**1.4291**	**2.9237**	**2.9150**	**3.4446**	**3.5226**	**3.9657**	**2.0140**

CUST_1233_PI416261804	OR51A7	1.5820	1.9409	1.4584	1.0574	0.8318	2.0525	1.6452	1.5488	1.4439	0.7432	1.2656	1.6471	1.5156	1.7573	1.6551	1.1022	0.7160	1.4384	1.5436	1.7479	1.4066	1.1687	0.8723	1.4243
A_23_P116640	OR51B2	1.9906	2.3370	3.1444	1.1938	0.9218	2.0196	2.1153	2.9214	2.0853	0.7432	3.8916	1.9890	1.8725	2.4423	2.1352	3.3669	1.0819	1.7991	1.8630	2.1547	1.7257	1.2339	1.2601	1.8784
CUST_653_PI416261804	OR51B2	2.1079	4.1196	1.6014	2.6848	0.8249	2.1215	2.2813	3.6395	2.0941	0.7432	3.1794	2.6253	1.8716	2.8041	1.5273	1.3009	0.8950	1.9214	2.2470	2.1289	1.9625	2.3538	1.9957	3.1108
A_23_P128023	OR51B4	2.0162	2.2927	1.9653	1.4035	1.4349	2.2767	2.0716	1.7393	1.7060	0.7432	1.9916	3.6721	1.7887	2.0594	1.6975	2.3507	0.8585	3.1660	1.8263	1.9909	2.2367	1.5497	1.5773	2.6756
CUST_2130_PI416261804	OR51B4	3.5469	3.1437	3.5457	1.4279	1.2713	2.9792	3.0968	2.0215	3.3534	1.9282	2.6960	1.8295	2.1436	1.8081	2.3519	2.4820	2.4998	1.6461	2.3497	2.1470	3.5418	2.7519	2.2952	2.5845
A_24_P58204	OR51B5	2.2788	1.9539	2.3966	1.1637	3.1877	2.7374	2.5722	1.5547	2.4521	1.2552	2.1413	1.6362	1.9134	1.8075	1.7308	1.0936	2.6494	1.6687	2.0774	1.7777	3.0123	1.2526	2.6733	3.6464
CUST_1050_PI416261804	OR51B5	2.0630	1.8760	2.8198	0.9638	0.9875	1.5722	2.4448	1.5407	1.9664	0.7432	1.6455	1.5706	1.7847	1.6737	1.5172	1.2959	0.9115	1.4828	1.9071	1.7501	3.1156	0.9283	1.2147	2.9552
CUST_339_PI416573500	OR51B6	2.0243	1.9123	1.5858	0.9745	1.2469	1.6178	2.3520	1.5556	1.6879	0.7432	2.9117	1.6087	1.7746	2.7781	1.3902	1.2937	0.8721	2.4762	1.8610	1.7355	2.5689	0.9692	2.0502	2.7395
CUST_348_PI416379584	OR51B6	2.3399	2.1235	2.8960	1.3497	3.2239	2.1441	2.8023	2.9220	2.1852	0.7432	3.1383	1.9444	1.9976	2.1010	2.0507	1.3673	3.3819	2.4468	2.2398	1.9140	2.5959	1.5906	3.3081	2.0113
A_23_P24898	OR51D1	1.8434	1.7961	5.7444	1.1180	2.2089	4.0131	2.0286	1.4216	1.8195	0.7432	1.2095	1.6233	1.7595	1.7475	1.5382	1.0235	0.7411	1.5602	1.7108	1.5700	1.4928	1.1695	1.2996	1.5812
CUST_2412_PI416261804	OR51D1	1.9103	1.5845	2.9621	0.8028	1.6651	1.4688	2.6591	1.2868	3.0896	1.8299	2.7305	1.4135	1.8050	2.5275	3.4498	0.9024	2.3561	1.5701	1.7458	1.7239	2.8772	1.0761	1.2298	1.6901
A_23_P13493	OR51E1	1.8420	1.9134	1.4617	1.4683	1.9537	1.7744	2.0548	1.5612	1.6830	0.7432	1.6725	1.7016	1.7635	1.8104	1.5663	1.4214	0.8951	1.6593	1.6837	1.6974	1.9793	1.1360	1.9393	1.7274
CUST_8724_PI416261804	OR51E1	2.6560	2.2040	3.2974	1.3782	2.7279	1.8622	2.9821	1.5700	3.1879	0.7432	2.8888	1.8227	2.1593	3.0723	2.7545	1.1730	1.2204	3.2721	2.3952	2.1604	3.1102	0.9897	2.5724	2.6464
A_23_P139327	OR51E2	2.2368	2.1299	2.2600	1.0774	2.1116	1.8125	2.5151	1.9225	2.2010	0.7432	1.8275	1.8917	1.9656	1.9519	1.5798	1.3685	2.2854	1.6660	1.9847	1.9824	1.6463	1.0873	1.7558	2.0822
A_24_P235756	OR51E2	1.8277	2.1279	1.7511	1.4061	1.1211	2.2019	1.9204	1.6895	1.5538	0.7432	1.4630	1.8323	1.6575	1.9839	1.6010	1.4113	0.7903	1.5976	1.7393	1.9333	1.4866	1.4711	1.0825	1.4745
CUST_53_PI416573500	OR51F1	1.8531	2.2503	1.6340	1.2555	2.3502	2.8803	1.7538	1.9834	1.5672	0.7432	1.7533	1.8851	1.5553	2.0155	2.7808	1.5129	1.0398	1.5012	1.7061	2.0826	1.4604	1.1953	1.0931	1.4319
CUST_57_PI416379584	OR51F1	1.9275	2.0323	1.3913	1.0571	2.7356	1.8180	2.0080	2.7399	1.6556	2.4204	1.1069	1.5837	1.6959	3.1037	1.5236	0.9199	0.6745	1.6307	1.7698	2.4646	1.8025	1.1551	1.5898	3.2022
CUST_341_PI416573500	OR51F2	1.7523	1.9071	1.4608	0.9419	0.9342	1.5796	2.0111	1.5329	1.4251	0.7432	1.3600	1.6484	1.6764	1.7030	1.5784	1.2038	0.9022	1.4546	1.6820	1.7928	1.6683	0.9158	0.9352	1.5891
CUST_350_PI416379584	OR51F2	1.6081	1.9524	1.4534	1.0520	0.8404	1.8072	1.6506	1.5255	1.4139	0.7432	1.1867	1.6277	1.5042	1.7106	1.5692	1.0791	0.6745	1.4263	1.5632	1.7575	1.3711	1.0897	0.8650	1.4224
A_23_P105118	OR51G1	1.7028	1.9968	2.2031	1.1697	3.2944	1.8465	1.7631	1.6487	1.7095	0.7432	1.4059	1.8051	1.6782	1.9229	2.2373	1.1636	0.8830	1.6544	1.6863	1.7919	2.0624	1.2127	1.9461	1.5278
CUST_2178_PI416261804	OR51G1	1.7122	2.0189	1.5444	1.2009	0.7850	1.9467	1.7480	1.6909	1.5429	0.7432	1.4176	1.7118	1.5990	1.8564	1.5693	1.1475	0.7300	1.4714	1.6072	1.7679	1.4165	1.2328	1.0557	2.0019
CUST_200_PI416573500	OR51G2	1.6804	1.9781	2.8760	1.1259	3.3123	1.7743	2.3945	2.2395	3.1899	2.9504	2.2517	1.7390	1.6255	1.8805	1.9343	2.0226	2.8298	1.5671	1.7426	1.7602	3.0501	1.1603	3.6165	1.4730

CUST_205_PI416379584	OR51G2	1.7368	1.9581	2.0427	1.2299	3.9297	1.7051	1.9472	3.4898	2.3965	3.1968	2.6186	1.6613	2.5090	1.8111	2.5736	1.1171	0.6933	1.4474	1.6429	1.7516	2.7920	1.2111	2.8697	1.4568
CUST_417_PI416573500	OR51I1	1.9693	2.1243	1.9808	1.4943	1.8081	2.1166	2.1025	1.7271	2.0693	2.0121	2.6820	1.9336	1.8632	2.1266	1.7302	1.6156	1.0329	1.7516	2.4747	1.9135	2.4345	1.5402	2.7190	1.5909
CUST_426_PI416379584	OR51I1	2.0690	1.9117	1.4838	1.0684	0.9887	2.0098	2.3869	1.5220	1.4179	0.7432	1.3356	1.6288	1.7525	1.7497	1.5233	1.2924	0.9116	2.8320	1.9822	1.9861	1.7688	1.0578	0.9239	3.1859
CUST_458_PI416573500	OR51I2	1.6969	2.1117	1.6543	1.2380	1.1229	1.9126	1.7218	1.6593	1.5631	0.7432	1.4803	1.8993	1.6560	2.0139	1.6853	1.3246	0.8778	1.6051	1.6570	1.9437	1.5177	1.2653	1.6329	1.4585
CUST_468_PI416379584	OR51I2	2.9473	2.6271	1.5855	1.0886	1.9758	1.9303	2.7426	2.7856	1.8412	3.0614	1.6227	3.2101	1.9292	1.9748	1.5315	1.4265	1.2066	1.9204	2.7462	2.4615	2.1851	2.5129	2.3631	2.2258
CUST_246_PI416573500	OR51L1	2.0699	2.0342	1.6345	1.2574	0.8240	1.9396	2.2780	1.5881	1.5976	0.7432	1.5377	1.7508	1.8723	1.9072	1.6244	1.2485	0.9843	1.6546	1.9163	1.8286	1.6135	1.2861	1.3480	1.7737
CUST_254_PI416379584	OR51L1	1.6538	1.8853	1.4662	1.0265	1.2612	1.6476	1.8584	1.5116	1.7257	0.7432	1.2858	1.6111	1.6193	1.7006	1.5745	1.2115	0.8121	1.5363	1.6005	1.7441	1.4135	1.0011	0.8645	1.4364
CUST_343_PI416573500	OR51M1	1.6836	1.9975	1.5375	1.1590	1.8905	1.8106	1.7201	1.5482	1.7453	0.7432	1.3965	1.7686	1.6581	1.8465	1.6437	1.1521	0.8708	1.5308	1.6197	1.7335	1.6689	1.1515	1.0291	1.4687
CUST_352_PI416379584	OR51M1	1.5435	1.8873	1.4174	0.8968	0.8079	1.5808	1.5158	2.5097	1.8543	0.7432	1.5608	1.8058	1.4889	1.6205	1.5536	1.0647	0.7378	1.6026	1.5462	2.0318	2.3427	1.0750	2.3526	1.6592
A_23_P2180	OR51Q1	1.7593	2.0676	1.7374	1.3940	1.2033	2.0442	1.8170	1.6494	1.5612	0.7432	1.5125	1.8881	1.6974	2.0236	1.6786	1.4482	0.9193	1.6304	1.8200	1.8924	1.5444	1.4041	1.0514	1.4628
CUST_190_PI416573500	OR51Q1	2.0280	1.9466	2.4526	1.2563	2.6379	1.9307	2.2474	1.5581	2.7470	0.7432	2.9245	1.7681	1.8562	1.9236	1.8560	1.3533	0.7792	1.6600	1.9310	1.7583	2.1967	1.3106	3.4391	1.6559
CUST_195_PI416379584	OR51Q1	1.5873	2.3844	1.4929	0.9977	0.8219	2.7885	1.6594	1.5380	1.6814	0.7432	1.1916	1.5840	1.5038	1.7030	1.5520	1.0569	0.6745	1.7609	1.9998	3.0061	1.6124	1.0123	0.8708	1.5901
CUST_414_PI416573500	OR51S1	2.7862	2.6909	4.7549	3.7163	5.1787	2.6708	3.0970	3.2948	4.7261	5.2418	4.5371	2.6860	2.4520	3.0068	4.7564	2.9039	5.3435	2.5229	3.4183	3.6036	4.0334	3.7275	5.5886	3.5875
CUST_423_PI416379584	OR51S1	2.0608	2.0797	2.2679	0.7920	0.7006	2.4073	2.4081	1.4157	1.6802	1.2152	1.0587	1.4135	1.7055	1.8381	1.3661	0.9270	0.7105	1.4773	1.7372	1.4951	3.0400	0.7432	0.9409	3.6148
CUST_192_PI416573500	OR51T1	1.7126	1.9269	2.0744	1.1664	3.1655	1.8681	1.7318	1.4972	2.0456	0.7432	1.2825	1.7673	1.6809	1.8675	2.3551	1.0472	0.7495	1.6339	1.6338	1.6780	1.5290	1.2171	1.7346	1.5311
CUST_197_PI416379584	OR51T1	2.0569	2.3491	2.4074	2.1469	3.4963	2.0916	2.2026	1.9765	2.4197	1.8567	2.7058	2.0943	1.8476	2.1519	2.3305	1.3737	1.0877	1.9551	1.9463	2.1523	1.5993	1.3541	5.3610	1.8193
CUST_423_PI416573500	OR51V1	2.1719	2.1124	1.8846	2.1564	1.6900	1.8590	2.5156	2.9952	1.5952	1.4060	2.1654	1.7270	1.8318	2.9952	1.5881	1.2986	0.8125	1.6189	2.1825	1.9078	1.7278	2.1648	1.1355	1.8705
CUST_432_PI416379584	OR51V1	2.0330	3.0082	1.5638	2.1274	1.4706	2.4612	2.2037	2.7399	1.5364	1.6572	1.6549	1.8006	1.6974	1.9580	1.5812	1.2592	1.0080	1.6963	2.2095	2.1065	1.6365	1.3163	1.6973	2.5876
A_23_P125286	OR52A1	1.7857	1.9608	2.0658	1.2009	1.0426	1.8515	1.9523	1.5333	2.0058	0.7432	1.3488	1.7809	1.6962	1.8732	1.7006	1.2811	0.8030	1.5581	1.7087	1.8137	1.6194	1.2210	2.2976	1.4809
CUST_995_PI416261804	OR52A1	2.2729	2.2666	2.5391	2.7786	2.4812	2.6532	2.4548	3.1099	2.0358	1.3089	1.6186	1.8694	1.9017	2.8479	1.9413	2.9852	1.0355	1.7697	2.0250	2.0759	2.7504	1.3156	1.4366	1.8919
CUST_37_PI416573500	OR52A4	1.7369	2.2005	1.7245	1.3344	1.0690	1.9518	1.7921	1.9169	1.6919	0.7432	1.7412	2.0322	1.7240	2.1380	1.8356	1.4795	1.2678	1.7477	1.6440	2.0312	1.4855	1.3190	1.1822	1.5576
CUST_39_PI416379584	OR52A4	1.6323	1.9332	1.4867	1.0919	0.9274	1.7848	1.6584	1.5141	1.4498	0.7432	1.2668	1.7152	1.5527	1.7747	1.6974	1.1334	2.3792	1.4805	1.5903	1.7845	1.4100	1.1203	0.9335	1.4467
CUST_277_PI416573500	OR52A5	2.1028	2.2868	2.0466	1.3153	3.2869	1.9617	2.3110	2.3447	2.4152	0.7432	1.7539	3.2034	1.8198	2.0226	1.7529	1.3816	1.0368	2.0072	1.8956	2.0811	2.5241	1.2864	2.2287	1.9207
CUST_285_PI416379584	OR52A5	1.8268	2.1912	1.5892	1.1820	1.1076	1.8841	2.0260	1.8720	1.5107	0.7432	1.6295	1.7727	1.6444	1.9216	1.6042	1.4866	0.9526	1.6538	1.8145	2.0262	1.3989	1.1848	1.0609	2.5966
A_23_P36203	OR52B2	1.6570	2.0163	1.8174	1.1325	2.4564	1.8710	1.7021	2.4969	2.2344	2.3465	1.3172	1.7838	1.6070	2.0866	1.7882	1.1887	2.9289	1.5360	1.6141	1.8714	1.4786	1.1368	2.2653	1.4728

CUST_1006_PI416261804	OR52B2	1.7005	1.7278	1.4019	0.9482	0.7340	1.6389	1.9055	1.3514	1.7090	0.7432	1.0634	1.5244	1.6895	1.6181	1.5400	1.2810	0.6916	1.6072	1.6275	1.5403	1.9330	1.0090	0.9162	1.5322
CUST_530_PI416573500	OR52B4	1.7392	2.1438	1.5514	1.2045	0.8420	1.9609	1.8107	1.7305	1.5495	0.7432	1.4933	1.7811	1.6412	1.8590	1.5507	1.2054	0.7363	5.0219	1.6614	1.8317	1.4593	1.2388	1.0799	1.4725
CUST_542_PI416379584	OR52B4	1.9663	1.9893	1.6489	0.9919	0.9340	2.1405	2.5714	1.6195	1.6384	0.7432	1.3660	2.9107	1.6812	1.6878	1.4590	1.2723	0.7842	1.9541	1.9481	2.7696	2.3238	2.8203	0.9228	1.6459
**CUST_555_PI416573500**	**OR52B6**	**5.8248**	**3.3224**	**5.1790**	**3.4560**	**6.5649**	**3.9397**	**4.9538**	**5.9287**	**5.2621**	**4.6704**	**5.4512**	**1.4985**	**3.9371**	**5.4145**	**4.4207**	**3.4950**	**4.5347**	**2.1943**	**5.2443**	**2.7182**	**5.7162**	**3.6219**	**5.6797**	**3.3505**
**CUST_571_PI416379584**	**OR52B6**	**2.4346**	**2.5208**	**1.4622**	**1.0272**	**0.9271**	**1.8239**	**2.5446**	**1.5304**	**1.3811**	**0.7432**	**1.2907**	**1.9031**	**1.7892**	**1.6682**	**1.4340**	**1.2474**	**0.7964**	**1.6199**	**2.0341**	**1.7388**	**2.0409**	**2.1098**	**0.9486**	**1.8008**
CUST_390_PI416573500	OR52D1	1.6188	1.8351	1.4322	0.9389	0.8206	1.5710	1.7942	1.4677	1.4488	0.7432	1.2472	1.5821	2.7651	2.8055	1.6011	1.0759	0.8240	1.4394	1.6240	1.7140	1.3888	0.9322	0.9172	1.4711
CUST_399_PI416379584	OR52D1	3.6757	2.0204	1.5530	1.1447	1.0331	1.8096	2.2989	1.6189	1.4912	0.7432	1.4238	1.7110	1.8018	2.5389	1.5962	1.3343	0.9878	1.6024	2.0729	2.4180	1.5382	1.1998	1.1288	1.7933
CUST_169_PI416573500	OR52E2	1.8559	2.3712	1.7755	1.4295	1.7978	2.1427	1.8891	1.8544	1.7232	0.8901	1.8160	2.1167	1.7311	2.1238	2.1284	1.3926	1.1406	1.8384	1.7706	2.2253	1.6032	1.4939	2.2703	2.1218
CUST_174_PI416379584	OR52E2	2.6216	2.5934	2.3894	1.0790	0.7880	1.8133	2.5499	2.9165	2.3173	3.3185	1.5315	3.4219	1.7716	1.7814	1.4059	1.2375	0.8376	3.1010	2.5463	3.1661	3.1548	3.0992	1.1053	2.6440
CUST_280_PI416573500	OR52E4	1.8945	2.3861	1.7207	1.4203	1.0066	2.2269	1.9351	1.8483	1.6317	1.5287	1.7054	1.9476	1.7302	2.1037	1.7534	1.3264	0.9340	1.7010	1.7120	2.1094	1.5954	1.5039	1.3199	1.6510
CUST_288_PI416379584	OR52E4	2.3744	2.2751	2.2886	2.7695	1.0441	2.0123	2.3225	3.7627	1.8624	0.7432	1.6749	1.7182	2.5024	3.2280	1.5389	1.2844	1.1157	1.9921	2.2138	3.8092	3.0267	2.5517	1.1744	3.7608
CUST_173_PI416573500	OR52E6	1.7732	2.1882	1.6948	1.3043	1.0929	1.8598	1.9400	1.8684	2.1069	0.7432	1.7077	1.9645	1.6810	2.1011	1.8852	1.4634	1.1786	1.6706	1.6249	2.0130	2.0424	1.2553	1.1250	1.5061
CUST_178_PI416379584	OR52E6	1.8650	1.7433	1.6071	1.4781	0.7258	1.8017	3.1290	1.4460	1.3427	0.7432	1.1137	1.4157	1.5984	1.4516	1.3805	0.9672	0.6954	1.4110	1.6682	2.6320	2.7058	1.1024	0.7949	2.3313
**CUST_326_PI416573500**	**OR52E8**	**1.9323**	**2.3470**	**1.7913**	**1.3317**	**1.3390**	**2.7356**	**2.0043**	**1.8139**	**1.8178**	**0.8575**	**1.7957**	**2.1449**	**1.7740**	**2.1059**	**1.9157**	**1.3839**	**1.2932**	**1.8896**	**1.9910**	**2.2696**	**1.6888**	**1.3468**	**1.4486**	**1.6699**
**CUST_335_PI416379584**	**OR52E8**	**2.7171**	**2.3799**	**4.3256**	**5.2054**	**5.2760**	**4.9296**	**4.1110**	**3.0748**	**4.1171**	**5.1926**	**5.3154**	**5.6897**	**1.8131**	**5.1226**	**1.8948**	**4.2346**	**1.1733**	**5.4739**	**4.1296**	**2.1278**	**4.9591**	**3.8825**	**5.7999**	**5.3317**
**CUST_142_PI416573500**	**OR52H1**	**2.1923**	**2.1252**	**1.6549**	**1.2491**	**1.5126**	**1.8773**	**2.3153**	**1.6480**	**2.4377**	**2.1136**	**1.5968**	**1.7852**	**1.8345**	**1.9321**	**1.6652**	**1.3175**	**0.9890**	**1.5861**	**1.9090**	**1.8701**	**1.8739**	**2.1616**	**1.2774**	**1.7139**
**CUST_147_PI416379584**	**OR52H1**	**2.5743**	**1.8483**	**1.3903**	**0.8607**	**1.1267**	**3.2401**	**2.8146**	**1.5033**	**1.3816**	**0.7432**	**1.1728**	**1.7730**	**3.1713**	**1.5295**	**1.4362**	**1.0046**	**0.6987**	**2.3066**	**2.4232**	**2.2836**	**1.9731**	**0.7432**	**0.8976**	**2.9440**
CUST_465_PI416573500	OR52I1	1.8322	2.1281	1.7468	1.4943	1.2917	2.1281	1.9154	1.7233	1.6455	0.7432	1.5988	1.9391	1.7567	2.1160	1.6963	1.5710	0.9989	1.7128	2.7194	1.9207	1.6049	1.5470	1.1405	1.5035
CUST_475_PI416379584	OR52I1	1.5748	1.8466	1.6350	0.9385	1.9128	1.7740	1.8239	1.8362	1.7761	0.7432	1.3122	1.5284	1.5681	1.6613	1.6163	1.1036	0.8286	1.5848	1.5176	1.6613	2.3544	0.8652	1.1115	1.8647
CUST_466_PI416573500	OR52I2	1.7074	1.9941	1.5787	1.2101	1.9322	1.8565	1.7809	1.6412	1.9347	0.7432	1.4901	1.7278	1.6413	1.8775	1.6295	1.2152	0.8807	5.0546	1.6025	1.7772	1.4438	1.2034	1.0975	1.4566
CUST_476_PI416379584	OR52I2	2.2264	2.2519	1.6381	1.1132	1.2662	1.6762	2.5487	1.8716	2.1007	0.7432	2.8266	1.8893	1.8563	1.9233	1.8053	1.4950	1.4353	1.6191	2.0239	2.1886	2.2501	1.0019	1.3224	2.0617
CUST_449_PI416573500	OR52J3	1.8929	2.1126	1.7631	1.4912	1.3452	2.1324	2.0016	1.7182	1.6498	0.7432	1.6125	1.9276	1.8069	2.1093	1.7287	1.6218	1.0957	1.7213	1.8174	1.9339	1.6116	1.5407	1.1375	1.5335
CUST_458_PI416379584	OR52J3	1.8699	2.0531	1.6723	0.9438	0.7585	1.6220	2.5605	1.7142	1.4632	0.7432	1.3940	1.6173	1.6815	1.6791	1.4730	1.1019	0.8333	1.6009	1.7662	1.7890	2.3539	0.8816	0.9702	2.2755
**CUST_327_PI416573500**	**OR52K1**	**1.7033**	**1.6752**	**1.3717**	**0.8410**	**1.5079**	**1.5210**	**1.9711**	**1.3421**	**1.4570**	**0.7432**	**1.0511**	**1.4520**	**1.6792**	**1.5293**	**1.4943**	**0.9301**	**0.6745**	**1.4606**	**1.6156**	**1.5100**	**2.6211**	**1.0746**	**0.8798**	**1.5482**

**CUST_336_PI416379584**	**OR52K1**	**1.8822**	**1.9915**	**1.4233**	**0.9340**	**1.0499**	**1.5470**	**2.2203**	**1.5559**	**1.4346**	**0.7432**	**1.5418**	**1.6171**	**1.7447**	**1.6832**	**1.4988**	**1.2839**	**0.9228**	**1.4392**	**1.7499**	**1.7109**	**2.8699**	**0.8787**	**0.9794**	**1.9485**
**A_23_P98744**	**OR52K2**	**3.1524**	**2.5483**	**4.4692**	**4.2791**	**5.3539**	**2.6335**	**3.4825**	**4.1649**	**4.5352**	**5.5518**	**4.4952**	**2.1603**	**4.1785**	**3.5218**	**4.0351**	**4.1327**	**3.9295**	**2.1594**	**3.4418**	**3.5865**	**4.2760**	**4.1998**	**5.2549**	**3.3350**
**CUST_2283_PI416261804**	**OR52K2**	**3.6564**	**3.1500**	**4.6607**	**3.9582**	**5.0969**	**2.9080**	**3.9998**	**3.1439**	**4.4723**	**4.9081**	**3.5118**	**2.4586**	**3.4686**	**2.0601**	**4.5935**	**3.0683**	**4.1391**	**2.5636**	**3.5697**	**3.6360**	**4.1825**	**4.0037**	**4.8442**	**3.7921**
**CUST_929_PI416573500**	**OR52L1**	**2.1228**	**2.2634**	**1.7909**	**2.3686**	**0.9636**	**2.1633**	**2.2567**	**1.6665**	**2.0535**	**0.7432**	**1.6802**	**1.9620**	**1.9144**	**2.0772**	**1.8071**	**1.3442**	**0.9987**	**1.8075**	**2.5609**	**1.9883**	**1.9561**	**1.5008**	**1.4530**	**1.8254**
**CUST_960_PI416379584**	**OR52L1**	**1.6621**	**1.8026**	**1.6467**	**1.0551**	**2.3917**	**1.7317**	**1.7406**	**1.4749**	**1.6656**	**0.7432**	**1.1910**	**1.5881**	**1.6123**	**1.7178**	**1.5536**	**1.0019**	**0.7095**	**1.4599**	**1.5727**	**1.5896**	**1.9074**	**1.0803**	**0.9573**	**1.4548**
**CUST_469_PI416573500**	**OR52M1**	**1.8972**	**2.1165**	**1.7397**	**1.3803**	**0.8948**	**2.0654**	**1.9788**	**1.6365**	**1.6848**	**0.7432**	**1.6000**	**1.8556**	**2.0763**	**2.0059**	**1.7213**	**1.2942**	**0.9939**	**1.6960**	**1.7620**	**1.8883**	**1.7403**	**1.4093**	**1.3492**	**1.6080**
**CUST_479_PI416379584**	**OR52M1**	**1.8809**	**3.1105**	**1.9595**	**0.9604**	**2.1745**	**1.6638**	**2.1724**	**2.3836**	**1.6729**	**2.4580**	**1.8835**	**1.6768**	**1.6928**	**2.2087**	**1.4866**	**1.2239**	**2.3501**	**2.0059**	**2.9381**	**3.1186**	**2.1621**	**0.9461**	**2.3338**	**2.2894**
CUST_366_PI416573500	OR52N1	3.1920	2.1841	3.0072	1.3399	3.0480	2.1414	2.2368	1.7422	2.3728	0.7432	2.2559	1.9477	2.4170	3.2544	2.2181	1.5121	2.3065	1.7448	1.8680	2.6398	1.9871	1.5726	2.6289	1.7441
CUST_375_PI416379584	OR52N1	1.7553	1.9982	1.8891	1.0359	0.7512	1.7259	1.9752	1.6372	1.4522	0.7432	1.3173	1.5368	1.6227	1.6989	1.4698	1.0776	0.7178	1.5148	1.5855	1.6951	1.7516	1.0181	0.9768	1.6097
CUST_328_PI416573500	OR52N2	5.3709	4.7276	5.5084	4.8694	5.8559	4.9342	4.8960	5.4838	5.5433	5.3530	4.8103	5.1189	3.5989	5.1023	5.6985	4.3416	4.8043	5.0156	5.1298	5.2750	5.0018	4.7943	5.5095	4.9692
CUST_337_PI416379584	OR52N2	1.7428	1.9479	1.5495	0.9431	0.8441	1.6915	2.1761	1.6289	1.4006	0.7432	1.3188	1.5523	1.6047	1.6412	1.4552	1.0746	0.7405	1.4397	1.6122	1.7855	1.6359	0.9748	0.9619	2.2910
CUST_930_PI416573500	OR52N4	1.6043	1.8732	2.0246	1.0220	1.7782	1.6066	1.7247	1.8162	1.5127	0.7432	2.6018	1.5909	1.5931	2.5472	1.6535	1.1026	0.7922	1.4256	1.5417	1.7181	1.8453	0.9831	1.8929	1.4246
CUST_961_PI416379584	OR52N4	3.0394	2.1438	3.5839	3.3108	2.1404	2.9654	3.5403	1.5830	2.5140	3.1357	2.2099	4.9272	2.5285	2.0091	1.6901	4.3900	1.4794	3.0650	3.0886	1.9221	3.4524	1.6388	1.8530	4.2490
CUST_210_PI416573500	OR52N5	1.6372	1.8261	1.7950	1.0174	1.2362	1.6671	1.7505	1.4713	2.0178	0.7432	2.1745	1.6187	1.6329	1.7393	1.6330	1.0439	0.7469	1.5121	1.5755	1.6330	2.5619	1.0439	1.8194	1.4616
CUST_216_PI416379584	OR52N5	1.9974	2.2256	2.5369	2.6677	1.4943	2.6135	2.5830	1.8181	1.7016	1.7872	2.1555	1.8478	1.7044	1.8999	1.7197	1.3829	2.1383	1.6798	1.8872	2.8573	2.8460	3.4590	2.0171	1.7023
CUST_289_PI416573500	OR52R1	3.7924	2.2849	4.3343	3.5865	4.9691	2.2772	3.9456	3.1521	4.4688	4.3188	3.9686	1.8637	2.4535	3.4034	4.6368	2.7730	4.0738	2.5620	3.3224	3.1805	3.9407	1.7634	5.0586	1.9947
CUST_297_PI416379584	OR52R1	1.9985	1.7530	2.0084	0.8414	0.8462	1.4337	2.3271	1.4632	1.3374	0.7432	1.2200	1.4551	1.6997	1.4876	1.3996	1.1105	0.8056	1.4186	1.8068	1.6468	3.1464	0.7432	0.8516	1.7917
CUST_330_PI416573500	OR52W1	5.1912	4.1953	6.0077	5.0389	6.1451	5.1441	5.3245	5.1718	6.0315	5.2731	4.9364	5.1731	4.6054	5.0007	5.8228	3.8120	4.9940	4.5841	5.9065	4.6152	5.6202	5.0603	5.8817	5.2572
CUST_339_PI416379584	OR52W1	2.0934	2.0925	2.3600	1.2330	3.5471	1.9422	2.3201	1.8362	2.7352	0.7432	2.2241	1.9247	1.9006	2.0352	1.7468	1.3314	1.0359	1.7983	2.4495	1.9239	1.8501	1.3034	2.9935	1.8966
CUST_209_PI416573500	OR56A1	1.6903	2.1383	1.6784	1.2594	1.1872	1.9126	1.7070	1.6927	1.5840	0.7432	2.3714	1.9378	4.5546	2.0311	1.7729	1.3498	0.9197	1.6091	1.6463	1.9944	1.5209	1.2580	1.9294	1.4665
CUST_214_PI416379584	OR56A1	2.0753	2.3851	3.8929	1.1505	0.8774	1.8992	1.5034	2.6379	1.7315	1.9451	1.6982	2.7128	1.5038	1.9466	1.6417	1.3346	1.0589	1.5428	1.6060	2.0508	3.1053	2.0711	4.1454	1.5633
CUST_573_PI416573500	OR56A3	1.7675	2.1056	1.6347	1.3642	1.1465	2.0939	1.8359	1.6906	1.5375	0.7432	1.4940	1.8397	1.6529	1.9752	1.6731	1.4043	0.8502	1.6061	1.7099	1.9463	1.5064	1.3974	1.0493	1.4892
CUST_589_PI416379584	OR56A3	1.8123	1.8466	2.0531	0.9184	0.8862	1.5481	2.1386	1.4967	1.3554	0.7432	1.2382	1.5327	1.7293	1.5922	1.4668	1.1739	0.7654	2.6415	1.8668	1.7071	2.5819	0.8816	0.8550	1.7864
CUST_437_PI416573500	OR56A4	1.8196	2.3284	2.9551	1.2403	1.6042	2.1487	1.8590	1.7507	3.5998	0.8293	1.6094	2.0169	1.7871	2.0731	2.7609	1.2516	1.1265	1.9188	1.7840	2.2634	3.7530	1.2959	2.6343	1.6972

CUST_446_PI416379584	OR56A4	1.9036	1.9139	1.4952	1.0523	1.0267	1.9685	2.2171	1.5643	1.4286	0.7432	1.3990	1.6265	1.7293	1.7398	1.5323	1.3384	0.9598	1.5308	1.7897	1.7792	2.0781	0.9918	0.9017	1.6112
CUST_1132_PI416573500	OR56A5	1.6373	1.7412	1.3884	0.9685	1.0863	1.6625	1.7352	1.9849	1.6366	0.7432	1.0654	1.5400	1.6149	1.6367	2.6843	0.9361	0.6745	1.4883	1.5804	1.5500	2.5646	1.0364	0.9055	1.4633
CUST_1170_PI416379584	OR56A5	1.7910	1.7665	2.3833	0.8677	0.7026	1.4459	2.1687	1.4873	1.4035	1.2345	1.2474	1.4594	1.6950	1.5509	1.4547	1.0546	0.8979	1.4142	1.6467	1.5672	2.7302	0.7432	0.8734	1.8187
A_23_P139250	OR56B1	1.7968	1.9429	1.5181	1.1938	0.9188	1.9302	1.9381	1.4973	1.4492	0.7432	1.2462	1.7152	1.7161	1.8352	1.6203	1.1534	0.7100	1.6431	1.7355	1.7570	1.5284	1.2897	1.0319	1.5665
CUST_4745_PI416261804	OR56B1	1.8365	2.4435	2.8367	1.7963	2.7011	2.2356	1.8338	3.1230	1.5282	0.7432	1.2747	1.7040	1.6506	1.8171	1.5224	1.0710	0.7277	2.4245	2.4978	2.6781	1.5039	1.9528	1.8033	1.4619
CUST_395_PI416573500	OR56B4	2.0243	1.8022	1.4463	0.9435	0.7748	1.5797	1.7195	1.4269	1.6823	0.7432	1.1742	1.5581	1.5910	1.6230	1.5945	2.5517	0.7539	1.4324	1.5584	1.6592	1.4006	0.9455	0.8845	1.4294
CUST_404_PI416379584	OR56B4	1.8597	2.0302	1.5646	1.1473	1.1784	2.3057	2.0429	2.8810	2.4555	0.7432	1.4328	1.7502	1.7398	1.8231	1.5922	1.1949	0.8765	1.5324	1.7355	1.7413	1.5192	1.1348	1.1876	1.5423
A_23_P75764	OR5A1	2.5670	2.4063	3.9352	2.2319	5.1970	2.3522	2.8118	3.5090	4.0921	4.3020	3.9607	2.1167	2.5113	3.5463	4.4720	3.7967	2.8479	1.9361	3.0038	2.6758	4.2766	2.4760	4.6458	2.4319
CUST_2295_PI416261804	OR5A1	3.4272	2.2696	3.1907	1.3108	1.0280	1.8521	2.7677	2.3981	2.7434	2.2028	1.6988	1.7680	1.8382	1.8179	1.5859	1.2988	2.1853	1.6005	2.1033	2.6861	4.1223	2.1916	2.7319	2.9308
CUST_159_PI416573500	OR5A2	2.4730	2.2927	2.6045	1.2262	3.2472	4.0021	2.6491	2.8944	2.7091	3.0208	2.5813	2.9240	1.9655	1.9131	1.7298	1.2136	0.7332	2.0190	2.5754	1.8992	2.6669	1.3104	2.9656	2.1030
CUST_164_PI416379584	OR5A2	2.1551	3.2450	2.6741	0.9290	0.9465	1.6140	1.8167	2.9594	1.7242	0.9331	1.3638	2.2256	1.5961	2.2329	1.6619	1.0910	0.8556	2.7315	1.6204	3.2610	3.2289	2.4213	0.9248	1.5338
CUST_406_PI416573500	OR5AC2	1.9183	2.4472	1.6989	1.3344	0.9840	2.1242	1.8964	2.1185	1.6370	0.7432	1.7176	2.0106	1.7625	2.6921	1.7635	1.3510	0.8202	1.7426	1.8513	2.5809	1.6523	1.4123	1.2830	1.6604
CUST_415_PI416379584	OR5AC2	1.6574	2.0818	1.5146	1.1591	0.9112	1.9052	1.7205	1.6922	1.6183	0.7432	1.3767	1.7364	1.5439	1.8686	1.6542	1.1933	0.7223	1.6643	1.6334	1.8716	1.4107	1.8999	0.9769	1.4566
A_23_P1863	OR5AK2	1.6367	1.9889	1.4759	1.1175	0.8138	1.8506	1.6841	1.6548	1.4644	0.7432	1.3184	1.7044	1.5349	1.8190	1.6376	1.0832	0.7026	1.4953	1.5722	1.7737	1.3833	1.1524	0.9638	1.4641
CUST_1666_PI416261804	OR5AK2	2.1644	2.5129	2.6430	1.2509	2.8398	1.9897	2.3718	2.0550	2.6688	0.7432	2.2599	1.7057	2.3681	1.9602	1.4727	1.4276	2.6269	1.7834	2.6000	3.5931	2.8307	1.3115	2.5844	2.6072
CUST_238_PI416573500	OR5AN1	1.8219	2.1258	1.7673	1.3812	0.8266	2.1299	1.7590	3.1170	1.6823	0.7432	1.5029	1.8814	1.7356	2.0105	1.6957	1.2312	0.8846	1.7147	1.7204	2.8460	1.5888	1.4865	1.2642	1.5495
CUST_245_PI416379584	OR5AN1	1.8794	2.2828	1.8016	1.2076	1.1332	1.9907	2.1764	1.8152	1.5389	0.7432	1.6464	1.8531	1.6899	1.9495	1.6701	1.2697	0.9715	1.6471	1.7419	2.0828	1.4284	1.2656	1.2569	1.6629
A_23_P47464	OR5AP2	1.8317	2.1270	1.8396	1.4913	1.3173	2.1043	1.9118	1.7191	1.6612	0.7432	1.6060	1.9468	1.7755	2.1266	1.7240	1.5887	1.0268	1.7232	3.9838	1.9180	1.6197	1.5374	1.3069	1.5043
CUST_11239_PI416261804	OR5AP2	2.9972	4.0487	3.3835	4.1130	4.7119	2.3431	3.5776	4.3999	4.1039	4.5754	4.5434	2.0406	2.7553	4.7378	1.7514	2.0031	3.9956	2.9569	4.4368	4.1720	3.5088	3.6817	5.2255	4.6888
CUST_1030_PI416573500	OR5AR1	2.1858	2.2292	2.4200	2.5564	3.5451	1.9232	2.4670	2.0005	1.8350	0.7432	2.1519	2.0025	1.8644	2.0983	2.1562	1.5909	1.8101	1.6366	1.9435	2.0456	3.1664	1.2697	3.2508	2.0282
CUST_1062_PI416379584	OR5AR1	1.8799	2.4059	1.6669	1.8439	1.1264	2.1219	1.9581	1.8033	1.5372	0.7432	1.6774	2.0418	1.7288	2.0324	1.7644	1.2834	1.0623	1.7754	1.8371	2.2794	1.6498	1.2538	1.2754	1.6749
CUST_432_PI416573500	OR5AS1	1.8995	2.3065	1.7818	1.4028	0.9653	2.1164	1.9173	1.6734	1.7011	0.7432	1.7201	1.9861	1.8078	2.0593	1.8127	1.3822	1.0128	1.7577	1.8115	1.9989	1.6479	1.4332	1.3667	2.1557

CUST_441_PI416379584	OR5AS1	1.9788	2.1851	1.5517	1.1084	1.0168	1.8073	2.2361	2.0051	1.4678	0.7432	1.9391	1.8357	1.7305	1.9103	1.6060	1.3566	1.0718	1.5607	1.7399	2.0338	1.3602	1.0642	1.1598	1.7667
CUST_178_PI416573500	OR5AU1	1.6054	1.7938	2.1519	0.9894	0.7401	1.6517	1.6637	1.4073	1.4567	0.7432	1.1401	1.5750	1.5723	1.6669	1.6306	0.9889	0.7173	1.4540	1.5568	1.6258	1.4316	1.0171	1.7132	1.4458
CUST_183_PI416379584	OR5AU1	2.1604	2.3902	3.0762	1.0702	1.7606	1.7089	2.4960	1.8185	3.5373	1.9563	2.5092	1.5630	1.7948	1.7257	2.5284	1.1964	0.8329	1.5578	1.9273	2.6375	2.5763	1.0529	1.1383	1.8139
CUST_180_PI416573500	OR5B12	1.8243	1.9456	2.6038	1.1725	3.0627	1.9085	1.9551	1.5142	1.7126	1.1255	1.2788	1.7856	1.7618	1.8874	2.1621	1.0414	0.7608	1.7375	1.8924	1.7017	1.8036	1.2521	1.7222	1.6304
CUST_185_PI416379584	OR5B12	2.1892	3.0394	1.9069	1.1780	0.7435	2.9239	1.8621	2.2542	1.5142	0.7432	1.4031	3.5054	1.7136	1.8407	1.4625	1.1350	0.7147	1.6946	1.6964	2.4203	1.8230	1.2661	2.1271	2.3211
CUST_247_PI416573500	OR5B17	2.7116	2.5074	3.4871	4.2482	4.3770	1.9988	3.7534	4.9289	3.6690	5.3830	3.3397	1.8792	2.7063	3.4590	2.2953	3.1584	4.2787	1.9115	3.0401	1.9700	3.7733	3.5446	4.2071	2.9273
CUST_255_PI416379584	OR5B17	1.9502	3.3496	1.5994	1.4690	1.0950	2.1418	2.0758	1.8331	1.4992	0.7432	1.6440	2.6389	1.7471	2.0160	1.7114	1.2649	0.9969	1.7516	1.8178	2.6632	2.0422	1.2312	1.2752	1.7597
CUST_302_PI416573500	OR5B2	1.7880	2.1857	1.9179	1.2204	0.7851	1.9011	1.8581	1.5904	1.6708	0.7432	1.5428	1.8713	1.7362	1.8834	1.6349	1.2655	0.8830	1.6214	1.7469	1.8409	1.5779	1.4302	1.7829	1.5103
CUST_310_PI416379584	OR5B2	2.1637	3.7098	1.9218	0.9541	0.8133	4.2577	2.2352	2.1317	1.7375	1.8882	1.5816	1.7170	1.7207	1.9069	1.5098	1.2538	3.1060	2.3695	1.9562	2.8929	2.0469	2.0178	1.4457	2.7664
CUST_35_PI416573500	OR5B21	2.3625	2.0275	2.9242	1.2089	3.9182	3.5894	2.6866	1.5854	3.1568	1.6852	3.0795	1.6914	2.0161	1.9447	2.6357	2.5502	2.7986	1.6810	6.2049	1.8463	3.5863	1.2839	3.1064	1.9455
CUST_37_PI416379584	OR5B21	1.8915	3.6426	1.6337	1.3336	0.9742	2.2556	2.0929	3.5198	1.6290	0.7432	1.4613	3.3303	1.6470	2.9507	1.7127	1.1999	0.7948	1.8762	1.8225	3.6138	2.3552	2.0561	1.5582	2.9144
CUST_239_PI416573500	OR5B3	1.6945	2.0985	1.5697	1.2132	0.9741	1.9010	1.7610	1.7666	1.5214	0.7432	1.5263	1.8259	1.6171	1.9596	1.7012	1.2764	0.8927	1.6026	1.6244	1.9124	1.4344	1.2577	1.0647	1.4926
CUST_246_PI416379584	OR5B3	2.1838	2.1339	1.7639	1.3320	1.2056	2.0524	2.4831	1.7764	1.7102	0.7432	1.6038	1.7833	1.8440	1.9765	1.5487	1.6004	1.0174	1.6031	2.0041	1.9547	2.2921	1.3097	1.1636	1.7514
CUST_299_PI416573500	OR5C1	3.0980	1.8033	3.4275	1.6841	3.3177	1.8083	2.9837	2.9408	3.2631	2.6014	3.3672	2.1929	2.6243	1.7804	3.2237	1.1912	2.2625	1.7975	2.3472	1.8714	2.6285	2.7454	3.6398	1.9751
CUST_307_PI416379584	OR5C1	3.3678	2.8094	3.7584	3.6887	4.9126	1.8902	3.5578	3.9315	3.9011	3.6834	3.9905	1.8830	2.6203	3.3817	3.5337	1.2481	3.8956	2.0497	3.1751	4.1057	3.6354	2.4605	4.8956	2.9199
CUST_386_PI416573500	OR5D13	1.8003	2.0419	1.6396	1.2279	2.8711	1.9163	1.8998	1.5741	1.6614	0.7432	1.4631	1.7867	1.7347	1.8736	1.6040	1.2116	0.8299	1.5803	1.7071	1.7558	1.5750	1.2565	2.1139	1.5118
CUST_395_PI416379584	OR5D13	1.7686	2.4762	1.6796	1.4624	1.0304	2.2051	1.7495	3.4257	1.5767	0.8404	1.6501	1.9844	2.1085	2.8479	1.5867	1.4090	0.9450	2.9917	1.7057	2.0063	1.8716	1.5151	1.2030	3.2919
CUST_401_PI416573500	OR5D14	1.8531	1.9466	2.4433	1.2585	1.7830	2.0221	1.9622	1.4914	2.3634	0.7432	1.5362	1.8001	1.7812	1.9418	1.7381	1.0577	0.9688	1.8148	1.8753	1.7167	3.2349	1.4184	4.1767	1.6826
CUST_410_PI416379584	OR5D14	1.7201	3.1065	1.5605	1.2060	0.8144	2.0176	1.8181	1.7480	1.5496	0.7432	1.5098	2.7273	1.6167	1.8765	1.5705	1.2208	0.7707	2.0163	1.6225	3.0388	1.7164	1.2544	1.0641	1.4370
CUST_448_PI416573500	OR5D16	1.6866	1.9904	1.5630	1.2046	1.0243	1.9046	1.7173	1.5664	1.4715	0.7432	1.3651	1.7986	1.5968	1.8854	1.6266	1.2604	0.7792	1.5278	1.6386	1.8351	1.4510	1.2442	0.9672	1.4462
CUST_457_PI416379584	OR5D16	2.0218	2.3902	1.5572	1.2088	0.8237	3.6548	2.0988	1.7437	1.5469	0.7432	1.5578	1.7902	1.7642	1.9207	2.6707	3.1490	0.7564	2.1406	1.7483	1.9308	2.1707	1.2632	1.5227	1.5042
CUST_230_PI416573500	OR5D18	1.6927	1.8784	1.5317	1.1526	1.0799	1.7978	1.7308	1.4990	1.5422	0.7432	1.3245	1.6764	1.6519	1.8169	1.6372	1.1072	0.8410	1.5048	1.5958	1.6728	1.4615	1.1617	1.0413	1.4698
CUST_237_PI416379584	OR5D18	2.5459	2.3704	1.9043	0.9665	0.8943	2.3638	3.0012	2.4898	2.3961	3.2641	1.9288	1.5936	3.8887	3.3449	2.5299	3.9955	0.9252	1.9023	2.6037	3.1895	2.4476	1.0600	1.1206	2.7380
A_23_P127684	OR5F1	3.3563	2.3177	4.2558	3.9073	4.9883	3.7342	3.8121	3.4364	3.9766	4.4377	3.4940	2.0027	2.7305	3.5931	4.1150	1.0396	3.5299	2.0302	4.0207	1.9183	3.8820	3.7064	4.5232	1.9791
CUST_1793_PI416261804	OR5F1	1.8004	1.8847	2.1847	1.0550	1.8179	1.7057	2.0254	1.4918	1.9025	0.7432	1.8942	1.6279	1.7023	1.7413	2.4457	1.1921	0.7465	1.5216	1.6955	1.7271	1.6222	1.0915	1.6053	1.6432

A_24_P366566	OR5H1	3.8168	2.4501	5.3231	4.6802	6.1607	3.8347	3.7458	4.6252	5.4150	5.6017	4.5961	2.9851	2.9753	4.5186	5.3070	3.2124	3.9947	3.4379	4.6139	3.8339	4.8801	5.0892	6.0458	3.8820
CUST_252_PI416261804	OR5H1	1.7304	2.1206	1.5405	1.1472	0.8762	1.8386	1.8731	1.8037	1.5846	0.7432	1.5554	1.8454	1.7027	1.9329	1.6712	1.2312	1.0539	1.6553	1.6445	1.9261	1.4314	1.1539	1.1108	1.5966
CUST_357_PI416573500	OR5H14	2.0533	2.1400	1.4657	0.9956	0.8312	1.6753	2.3879	1.8339	1.5013	0.7432	1.5599	1.7641	1.8329	1.8223	1.5939	1.2314	1.0885	5.9889	1.8735	1.9478	1.4560	0.9607	1.7230	1.9603
CUST_366_PI416379584	OR5H14	2.6203	2.1570	2.5136	2.4125	3.4358	2.9201	2.8373	1.6629	2.4584	2.6359	2.6431	2.8428	2.0859	2.2696	2.2977	2.9167	3.0467	1.8418	2.4976	1.9289	2.0727	3.2855	3.4469	3.2880
CUST_42_PI416573500	OR5H15	1.6802	1.9986	1.5358	1.1926	0.9799	1.8954	1.7281	1.5738	1.4606	0.7432	1.3417	1.7437	1.5791	1.8488	1.6493	1.2523	0.7531	1.5129	1.6337	1.8362	1.4580	1.2345	0.9367	1.4525
CUST_45_PI416379584	OR5H15	1.9597	2.2486	1.7468	1.5100	1.8347	2.3567	2.0635	1.8335	1.6396	0.8926	1.6169	1.9408	1.7503	2.1104	1.6816	1.6004	0.9498	1.7019	1.8294	2.0741	1.5761	1.6432	1.7818	1.5947
CUST_410_PI416573500	OR5H2	1.8527	2.2242	1.6850	1.3301	1.5315	2.0498	1.9152	1.7431	1.6530	0.7432	2.6579	1.9093	1.7398	2.0138	1.8863	2.3921	1.0195	1.7685	1.7603	2.0666	1.5564	1.4338	1.7358	1.6430
CUST_419_PI416379584	OR5H2	1.6188	2.3419	1.4233	1.0245	0.7963	1.8278	1.6524	2.9159	1.3898	1.9226	1.1167	1.5703	1.5068	3.3497	1.4912	1.0198	0.6745	1.5518	1.5718	1.6982	1.3589	1.1089	0.8552	1.6589
CUST_186_PI416573500	OR5H6	1.7948	1.8984	3.2536	1.5882	1.9821	1.9198	1.9341	1.4510	2.1399	0.7432	1.1601	1.6808	1.6771	1.7759	2.1973	1.1024	2.3571	1.5821	1.7250	1.7167	1.9066	1.2540	2.7910	1.5183
CUST_191_PI416379584	OR5H6	1.8550	2.4055	1.6926	1.3421	1.2793	2.2172	1.8928	1.8830	1.6139	0.9169	1.7752	2.0897	1.7193	2.1056	1.7982	1.3499	1.1023	1.8177	1.7582	2.2685	1.5640	1.4020	1.3606	1.6479
A_23_P150467	OR5I1	1.6804	1.9787	1.6393	1.8054	2.6620	1.8490	1.7602	1.5613	1.4393	0.8816	1.9399	1.7578	1.6012	1.8300	1.6334	1.2195	2.4810	1.4997	1.6363	1.8302	1.4354	1.1719	1.4959	1.4472
CUST_11372_PI416261804	OR5I1	1.6620	5.4123	1.5016	1.0869	0.9202	1.8007	1.6745	6.9822	1.4665	0.7432	1.2915	1.7091	1.5998	5.5719	1.6314	1.0973	1.3355	1.5429	1.6218	5.8699	1.4253	1.1499	1.0091	1.4813
CUST_15_PI416573500	OR5J2	2.0483	2.1639	1.7184	1.1108	0.9199	1.7855	2.3044	1.9103	1.4875	0.7432	1.6528	1.8472	1.8046	3.7474	1.6386	1.3222	1.1601	1.6453	1.8830	1.9904	2.2159	1.0816	1.1709	1.8267
CUST_17_PI416379584	OR5J2	2.8289	3.6728	2.7838	0.9276	0.7840	3.7340	2.3036	3.5538	2.7651	2.3018	1.4541	1.9039	1.5932	3.7350	1.4181	1.7994	0.9445	3.2366	2.2833	3.7816	4.1680	2.2525	0.8822	4.2127
CUST_533_PI416573500	OR5K1	2.0429	1.9911	2.5013	2.5523	1.7626	2.5709	2.2242	1.7406	1.5629	0.7432	1.2433	2.9414	2.2449	1.9722	1.7449	2.0615	0.7338	1.9765	2.3495	2.2643	2.4437	1.4765	1.3751	2.7564
CUST_546_PI416379584	OR5K1	1.6717	2.0001	1.5091	1.1614	0.9316	1.9539	1.7050	1.5658	1.4522	0.7432	1.2795	1.7299	1.5341	1.8138	1.5541	1.1863	0.6745	1.4770	1.6146	1.8194	1.3871	1.2211	0.9371	1.4398
CUST_283_PI416573500	OR5K2	1.7730	2.1349	1.9909	1.2054	1.0383	1.8482	1.8095	1.8577	1.6373	0.7432	1.6204	1.8230	1.6891	1.9288	1.7574	1.5204	1.0913	1.6392	1.6690	1.9968	1.4962	1.2329	1.2260	1.5386
CUST_291_PI416379584	OR5K2	1.7775	2.0502	1.4763	1.3538	0.8742	2.3975	2.0120	1.8408	1.5138	0.7432	1.5331	1.6804	1.6436	1.7792	1.5154	1.2291	0.9504	1.4924	1.5901	1.8731	1.4444	0.9831	1.0385	1.5906
CUST_32_PI416573500	OR5K3	1.7615	2.0992	1.7058	1.3231	0.9089	2.0156	1.8106	1.7464	1.6323	0.7432	1.5540	1.7787	1.6576	1.9739	1.6699	1.2513	0.8751	1.5664	1.6445	1.9013	1.4788	1.3333	1.2142	1.5032
CUST_34_PI416379584	OR5K3	2.6859	3.1653	3.5375	3.4305	4.0682	3.0422	3.1178	3.8774	3.5853	3.0477	3.8896	1.5824	2.1973	3.4254	2.7843	2.4544	2.9861	2.6976	3.3597	2.9395	2.7811	3.2031	4.1135	3.5843
CUST_41_PI416573500	OR5K4	1.6853	2.0741	1.5370	1.1812	0.9801	1.9882	1.7094	1.6363	1.4615	0.7432	2.3990	1.7858	1.5561	1.8875	1.5579	1.2193	0.6954	1.5083	1.6243	1.8933	1.3924	1.8420	1.0025	1.4425
CUST_43_PI416379584	OR5K4	1.7351	3.1998	1.3807	0.8885	0.7137	1.4846	2.0421	2.6422	1.3622	0.7432	1.1342	1.4135	1.5575	1.4641	1.3075	0.9463	0.6745	1.7627	1.9003	1.5958	2.0514	0.7688	0.7907	1.5985
CUST_403_PI416573500	OR5L1	2.6438	1.8691	2.7214	1.0139	3.0057	1.7119	3.0398	2.7433	2.7163	1.3461	1.2892	1.5273	2.0812	1.6660	2.1768	1.0558	2.3380	1.6628	2.3684	1.7143	3.1827	1.0860	2.2073	2.3609
CUST_412_PI416379584	OR5L1	2.5648	3.7963	1.9142	1.6138	0.8928	3.5293	2.2630	2.0098	1.5264	0.9197	3.4004	3.8158	1.8399	2.0817	1.6108	1.3869	1.0464	2.3998	1.9726	3.1219	2.1953	1.3579	1.2998	3.5901
A_24_P258235	OR5L2	9.1148	8.2364	8.6448	6.8087	8.5240	8.9081	8.8247	7.9850	8.3494	6.5957	7.3278	8.7169	6.6631	8.8495	8.8615	6.2619	7.4311	8.4280	8.2242	7.5277	7.9545	6.7076	7.5277	7.5706

CUST_839_PI416261804	OR5L2	1.7684	1.7169	2.3802	0.8550	1.1063	1.5518	2.0522	1.3443	2.2469	1.6749	1.1025	1.4692	1.7065	1.5708	1.7225	0.9240	1.1359	1.4859	1.6379	1.5131	3.0819	0.9246	1.4366	1.5988
CUST_285_PI416573500	OR5M1	1.9991	2.3521	1.9995	3.0886	1.5673	1.9895	2.1578	1.8185	2.6578	3.6974	4.0183	2.0393	1.7990	1.9982	2.1132	2.8083	1.2591	1.7389	1.8719	2.2253	1.5946	1.2358	2.0351	1.8012
CUST_293_PI416379584	OR5M1	1.6646	2.2428	1.4907	1.1159	0.9149	2.3704	1.7093	1.5628	1.4311	0.7432	1.2702	1.7034	1.5431	2.1383	1.5347	1.1591	0.6745	1.7437	1.6079	1.8344	1.3678	1.1694	0.9350	1.8593
CUST_405_PI416573500	OR5M10	1.8240	2.1771	2.3587	1.2053	2.6135	1.9132	1.9826	2.0479	2.8030	1.8348	1.5317	1.9213	1.7761	2.0018	1.7891	1.2399	1.5413	1.7770	1.7115	1.9598	2.1530	1.2361	3.4009	1.6904
CUST_414_PI416379584	OR5M10	1.9680	3.4197	1.6509	1.3146	0.9347	2.1168	2.0398	1.7945	1.8040	0.7432	1.6646	1.8665	1.7474	1.9765	1.6336	1.3140	0.8227	1.8965	1.8079	2.4958	1.5532	1.3621	1.2649	1.6535
CUST_256_PI416573500	OR5M11	2.0345	2.1938	1.6908	1.2951	2.1105	2.4504	2.1864	1.6546	1.6646	0.7432	1.6733	1.8530	1.8289	1.9622	1.9910	1.3480	1.0271	1.6506	1.9200	1.9891	2.2359	1.2844	1.3174	1.7020
CUST_264_PI416379584	OR5M11	1.6053	1.7489	1.3838	0.8689	0.7110	1.5183	1.8461	1.4442	2.7281	0.7432	1.1299	1.4502	1.5932	1.5518	1.4707	0.9692	0.7090	1.4121	1.5338	1.5634	1.9463	0.8404	0.8542	1.6042
CUST_287_PI416573500	OR5M3	1.8235	2.3251	1.7001	1.3676	1.1369	2.0891	1.8712	1.8577	1.6590	0.7688	1.7476	1.9562	1.7060	2.0653	1.7629	1.3232	1.0658	1.7343	1.7218	2.1549	1.5388	1.4339	1.3244	1.6067
CUST_295_PI416379584	OR5M3	2.0026	3.5346	2.7117	1.1148	0.8896	2.3764	2.9075	2.0515	1.6754	2.7140	1.6935	2.8081	1.9313	2.6548	1.5601	1.4389	1.5117	1.8871	1.7875	3.7136	2.9779	1.0146	1.1286	3.7289
CUST_306_PI416573500	OR5M8	1.6442	1.8028	1.4937	1.0425	1.3807	1.6901	1.7097	1.4486	1.8777	0.7432	1.7968	1.5838	1.6092	1.7178	1.6188	1.0255	0.7495	1.4591	1.5764	1.6250	2.6383	1.0559	1.6650	1.4469
CUST_314_PI416379584	OR5M8	1.9347	2.0367	1.9146	1.0735	0.8936	1.7959	2.0283	1.5953	1.4042	0.7432	1.2885	1.6304	1.6255	1.7461	1.5625	1.1912	0.6916	1.5873	1.6885	1.8339	1.5912	1.0793	0.9289	1.6503
CUST_407_PI416573500	OR5M9	2.0632	2.0790	2.9876	1.4192	3.3003	2.1132	2.2264	2.2671	3.1640	2.0856	2.8084	1.9097	1.8349	2.0576	2.3703	2.0614	2.7411	1.7576	1.9805	1.8701	2.0539	1.5318	4.1149	1.6527
CUST_416_PI416379584	OR5M9	1.9669	2.7440	2.8078	2.1244	0.9660	3.5306	2.7873	4.0149	1.4573	2.1907	2.1817	1.8841	1.7173	3.5669	1.5639	1.3034	1.0265	2.9509	1.8261	4.2836	2.3966	2.6210	1.1290	2.2924
A_23_P47125	OR5P2	1.6288	1.9350	1.5074	1.0589	2.4673	1.6946	1.6808	1.5309	1.5435	0.7432	1.3670	1.6789	1.5952	1.7576	1.6933	1.1289	1.8367	1.4911	1.5847	1.8067	1.4320	1.0658	0.9853	1.4528
CUST_1455_PI416261804	OR5P2	1.8216	2.0134	2.5447	2.0509	2.5741	2.1045	1.8815	1.5299	2.5616	0.7432	1.2299	1.8182	1.6966	1.9244	2.0446	2.8601	1.0060	1.8860	1.7441	1.8342	1.8016	1.3722	1.9346	1.5815
A_23_P399217	OR5P3	2.2294	3.7963	1.7570	1.3874	1.1094	3.7548	1.8348	1.8314	1.6466	0.9024	1.6454	1.9298	1.6758	2.0769	1.7274	2.5950	0.9366	1.8449	1.7260	2.1197	1.5203	1.5582	1.3213	1.6266
CUST_2063_PI416261804	OR5P3	1.8348	2.1316	1.6313	1.4079	1.0993	2.1894	1.9125	1.7071	1.5625	0.7432	1.4968	1.8513	1.6935	2.0149	1.5564	1.4207	0.7770	1.6238	1.7578	1.9140	1.5041	1.4803	1.1212	1.4765
CUST_184_PI416573500	OR5R1	1.7474	2.7862	1.5061	1.0861	0.9829	1.7472	1.9570	1.6790	1.4977	0.7432	1.4596	1.8026	1.7332	1.8607	1.6687	1.1744	1.0171	1.6048	1.6552	1.8637	1.8357	1.0581	1.3751	1.6192
CUST_189_PI416379584	OR5R1	2.8720	2.0899	1.6316	2.0286	0.8776	1.6334	3.1576	3.2261	1.9281	0.7432	1.3124	2.5617	1.8119	1.6752	1.2790	2.9118	1.0435	1.5580	2.1413	3.3556	2.0326	0.9199	0.9624	3.9349
CUST_409_PI416573500	OR5T1	1.8110	2.2756	1.6756	1.3505	1.0635	2.0873	1.8627	1.8535	1.6462	0.7550	1.6867	1.8770	1.6816	2.0353	1.7173	1.2919	0.9825	1.6704	1.6963	2.0945	1.5051	1.4095	1.2887	1.5604
CUST_418_PI416379584	OR5T1	1.7376	2.3788	1.6068	1.1680	1.0034	1.9029	1.8393	2.9775	1.5419	0.7432	1.5776	1.7598	1.6255	1.9129	1.6441	1.2316	0.9228	1.5461	1.6412	2.0826	1.4161	1.2136	1.1497	1.7046
A_23_P87104	OR5T2	2.1736	2.1820	2.4786	1.2322	1.1638	1.9883	2.4319	2.1676	2.1868	0.7432	2.3576	1.9320	2.3153	1.9752	1.7787	2.6144	1.9326	2.2724	2.0327	2.0817	1.5900	1.2807	1.8953	1.9826
CUST_1226_PI416261804	OR5T2	2.2481	2.2085	2.0208	1.3546	3.7816	2.0968	2.2251	3.3062	2.6375	3.4009	1.3957	1.8964	2.8664	1.9956	1.6934	1.1943	0.6933	1.7373	2.1401	1.8716	2.2498	1.3999	3.0821	1.5441
CUST_337_PI416573500	OR5T3	1.8084	2.1910	2.9140	2.9035	3.8878	2.8448	1.9306	2.4128	2.4535	1.4728	3.1728	1.9104	1.6805	1.9935	2.8820	1.1427	2.1359	1.6971	1.6954	2.6603	2.4101	1.3114	2.2107	1.5520

CUST_346_PI416379584	OR5T3	1.7075	1.9420	1.5218	0.9196	0.7661	1.6249	1.9493	1.5711	1.6392	0.7432	1.2535	1.5720	1.7567	1.6446	1.4756	1.0389	0.7152	1.4142	1.7209	1.7264	2.2702	1.3033	0.9204	1.5069
**A_23_P42241**	**OR5V1**	**4.0242**	**4.7718**	**5.5486**	**5.1647**	**5.9859**	**2.5841**	**4.4313**	**4.0869**	**5.1961**	**5.6733**	**4.7423**	**5.0314**	**2.9392**	**4.5009**	**5.6086**	**3.0406**	**5.0774**	**4.2917**	**4.6723**	**4.4871**	**4.9047**	**5.8196**	**6.1169**	**3.2531**
**CUST_4707_PI416261804**	**OR5V1**	**1.6847**	**2.1023**	**1.7619**	**1.1668**	**1.0384**	**1.9406**	**1.7112**	**1.6717**	**1.4604**	**0.7432**	**1.4285**	**1.8295**	**1.5907**	**1.9333**	**1.5990**	**1.2355**	**0.7972**	**1.5564**	**1.6298**	**1.9523**	**1.4130**	**1.2227**	**1.0837**	**1.4632**
CUST_163_PI416573500	OR5W2	1.7540	1.7059	2.9985	0.8660	1.5866	1.4961	2.0675	1.4170	3.4615	0.7432	2.6964	1.4653	1.6982	1.5606	3.4578	0.9773	1.0346	1.4451	1.7004	1.5216	2.9203	0.8293	2.1717	2.0201
CUST_168_PI416379584	OR5W2	1.8202	2.2634	1.9568	1.5665	1.1497	2.6882	1.8718	2.4382	2.2817	1.2592	1.6749	1.9347	1.6975	2.0195	1.9271	3.4829	1.0234	1.7531	1.9119	2.1018	1.5001	1.4055	1.5793	1.6027
A_23_P64448	OR6A2	1.6905	1.8893	1.5310	1.1327	0.7547	1.7689	1.7531	1.5058	1.5433	0.7432	1.3073	1.6590	2.0077	1.8102	1.6684	1.0992	0.8297	1.5029	1.5975	1.7113	3.3687	1.1433	1.6694	1.4757
CUST_1951_PI416261804	OR6A2	2.0585	1.9264	2.3050	1.1196	1.0028	1.7702	2.3379	1.5428	2.0257	0.7432	2.4572	1.7050	1.8148	1.8327	1.4976	1.2897	0.9802	1.5694	1.9179	1.7499	2.6805	1.1483	2.3352	1.6863
CUST_599_PI416573500	OR6B1	1.6668	1.8375	1.8846	1.0718	0.8656	1.7484	1.7389	1.4662	1.4821	0.7432	1.2421	1.6632	1.6575	1.7686	1.6094	1.0216	1.0990	1.5328	1.5920	1.6131	1.4450	1.0993	1.0639	1.4819
CUST_615_PI416379584	OR6B1	2.1188	2.1443	1.7743	1.1117	0.8770	1.8438	2.3868	1.9350	1.5467	0.7432	1.6434	1.9017	1.9087	1.9735	1.5716	1.3851	2.4176	1.6709	2.6874	1.9845	2.0249	1.1323	1.1957	2.0501
CUST_368_PI416379584	OR6B2	2.2125	2.0923	1.9139	1.7793	2.8060	3.0193	2.6869	2.7725	1.4815	0.8652	2.2818	1.7511	5.0066	1.9368	1.8581	1.3135	0.8738	2.3189	2.1772	1.8713	1.8492	2.1107	2.0872	1.9978
CUST_359_PI416573500	OR6B3	2.3877	1.6485	3.4236	1.0543	2.9535	1.7814	2.8114	1.1723	3.4481	0.7432	2.2880	1.6371	2.2331	1.6848	2.5259	1.4444	2.9572	1.8288	2.3021	1.5224	2.6884	1.2029	4.7675	2.1973
CUST_562_PI416573500	OR6B3	2.0526	2.1111	1.9063	1.1711	0.7972	1.8803	2.3329	1.6559	1.9348	0.7432	1.4368	1.8716	1.9296	1.9495	1.8194	1.1624	1.0327	1.8199	1.9236	1.8740	1.6084	1.2054	1.2291	1.9306
CUST_578_PI416379584	OR6B3	2.2169	2.1900	1.8538	2.3557	1.6448	2.0293	3.0444	1.7361	1.9029	0.7432	1.5905	1.9466	2.9968	2.0639	2.5594	2.8962	2.2124	2.2391	2.1694	2.7597	1.7763	1.3784	1.6207	2.3732
CUST_177_PI416573500	OR6C1	1.7970	2.0236	1.6226	1.2500	1.5074	1.9768	1.8959	1.6596	1.5645	0.7432	1.4654	1.7526	1.6834	1.8900	1.6060	1.1965	0.7810	1.5406	1.6741	1.7758	1.4774	1.2842	1.9548	1.4980
CUST_182_PI416379584	OR6C1	1.6604	2.0151	1.4343	0.9721	0.7541	1.6559	1.9145	1.6372	1.5113	0.7432	1.3268	1.6280	1.6209	1.7160	1.5302	1.0841	0.7970	1.4363	1.6999	1.7140	1.3629	0.9511	0.9566	1.5022
A_23_P368909	OR6C2	2.5478	2.1839	2.8421	1.2443	3.5634	2.1292	2.8095	1.5355	3.1401	0.7432	1.8011	2.0075	2.1386	1.9602	2.0016	1.1558	0.9341	2.3027	2.9734	2.1056	2.0437	1.3510	3.3348	2.3013
CUST_3783_PI416261804	OR6C2	1.8689	1.8104	1.9067	0.8823	0.8752	1.5015	2.2214	1.4679	1.4431	0.7432	1.7334	1.5373	1.6977	1.5820	1.4807	1.1506	0.8459	1.4338	1.7454	1.6905	2.5870	0.7688	0.8796	2.1816
CUST_3813_PI416261804	OR6C2	2.3747	2.5337	2.2045	0.9451	0.9369	1.5381	2.8641	1.4792	2.1332	2.4293	2.8251	2.8135	1.9550	1.6932	1.5164	1.3244	1.1412	2.0694	2.2089	1.7193	2.9554	0.8909	1.1584	2.0803
CUST_182_PI416573500	OR6C3	1.6668	1.9325	2.2662	1.3075	2.6234	1.8056	1.8788	1.4850	2.3424	0.7432	1.3468	1.7613	1.6056	1.8155	2.0857	1.1506	2.2703	1.5938	1.6203	1.7848	1.4678	1.1597	2.7446	1.4729
CUST_187_PI416379584	OR6C3	1.8522	2.4423	1.6692	1.3357	1.2623	2.2126	1.8975	1.8900	1.5890	0.8991	1.7596	2.0432	1.7561	2.0952	1.7612	1.3388	1.0798	2.0529	1.7462	2.2496	1.5196	1.4050	3.7419	1.6346
CUST_422_PI416573500	OR6C4	1.7155	2.0796	1.6731	1.3286	1.1866	1.9520	1.7382	1.6409	1.5998	0.7432	1.5005	1.9063	1.6609	2.0118	1.7457	1.3977	0.9166	1.5984	1.6737	1.9180	1.5358	1.3080	1.0178	1.4591
CUST_431_PI416379584	OR6C4	2.0871	2.3005	1.9631	1.5111	1.0078	2.3452	1.9049	1.8416	1.7059	0.8735	1.6663	1.9258	1.7185	2.1129	1.7273	1.8558	0.8571	1.7110	2.1813	2.0230	1.5634	1.6447	1.3513	1.5635
CUST_29_PI416573500	OR6C6	1.7341	2.1691	1.6163	1.2651	0.9320	1.9902	1.7620	1.8102	1.5571	0.7432	1.5662	1.9390	1.6963	2.0318	1.7494	1.2426	1.0346	1.7532	1.6669	1.9572	3.1062	1.3003	1.1682	1.5797

CUST_31_PI416379584	OR6C6	1.7484	1.9350	1.4491	0.9945	0.8593	1.7252	1.9071	1.5422	1.3750	0.7432	1.2203	1.5538	1.5766	1.6456	1.4697	1.1452	0.6745	1.4316	1.6675	1.7530	1.3461	1.0030	0.8686	1.4468
CUST_45_PI416573500	OR6C65	5.6052	6.7829	5.2065	6.6102	6.0554	6.1657	6.9248	6.2612	4.9687	6.3194	5.0358	4.3354	4.4034	6.9070	4.7242	5.1250	5.3944	4.4774	5.2606	6.4460	5.0969	6.3125	5.7442	4.9153
CUST_48_PI416379584	OR6C65	2.1017	1.9117	1.4491	1.0659	0.7299	2.0503	1.8930	1.5788	1.5693	1.1574	1.2555	1.6079	1.6135	1.8052	1.5188	1.0388	0.7010	1.4473	1.8856	1.6641	1.3902	1.0756	1.2760	3.2175
CUST_34_PI416573500	OR6C68	1.5900	1.8635	1.4177	0.9497	0.7656	1.6732	1.7211	1.5481	1.4104	0.7432	1.2008	1.5605	1.5342	1.6676	1.5399	1.0156	0.6808	1.4271	1.5292	1.6608	1.3398	1.9633	0.8755	1.4221
CUST_36_PI416379584	OR6C68	1.8930	2.2932	1.4408	0.9423	0.7712	1.6246	2.2334	1.7397	1.3909	0.7432	1.3995	1.6025	1.6895	1.6559	1.4634	1.0936	0.8262	1.4591	1.7316	2.7647	1.3831	0.8826	0.9812	2.0190
CUST_44_PI416573500	OR6C70	1.7998	2.2756	1.6700	1.2928	1.2664	2.0896	1.8547	1.8372	1.6017	0.8936	1.7188	1.9897	1.7020	2.0550	1.7673	1.3236	1.0866	1.7641	1.7252	2.1658	1.4849	1.3429	1.3487	1.6195
CUST_47_PI416379584	OR6C70	1.8182	2.2329	1.6652	1.5663	1.1591	2.1626	1.9131	1.8463	1.5763	0.7432	1.6071	1.8785	1.6670	2.0739	1.6193	1.5012	0.9268	1.8068	1.7079	2.8134	1.4879	1.4505	1.1477	1.5203
CUST_1120_PI416573500	OR6C74	2.6016	4.2961	3.4867	6.5980	4.0847	1.9408	3.1247	4.1922	2.8577	4.4499	2.6359	3.3206	1.5645	4.6337	3.2542	3.0321	1.5260	1.4363	1.7891	4.3624	2.7151	2.9352	3.4116	2.9384
CUST_1157_PI416379584	OR6C74	4.5110	2.9324	3.8107	4.0162	4.4634	2.3931	4.6750	3.8573	3.7993	4.7456	3.8155	1.8336	2.4610	3.0259	3.8361	2.7816	3.2353	2.4360	4.1641	3.0023	3.5312	4.7015	4.8353	3.0565
CUST_31_PI416573500	OR6C75	1.7514	2.0333	1.6322	1.2440	1.3234	1.8828	1.8365	1.5978	1.6249	0.7432	1.5323	1.7856	1.7025	1.9048	1.6622	1.2596	0.9439	1.5470	1.6520	1.7903	1.4993	1.2357	1.6135	1.4735
CUST_33_PI416379584	OR6C75	2.4241	2.4934	1.7055	1.9479	2.5412	2.4780	2.7227	2.0281	1.8064	2.1912	1.6294	2.5221	2.5283	1.9716	1.5516	2.3512	0.9447	2.0402	2.2163	2.4247	1.5432	1.1482	1.2675	2.9709
CUST_36_PI416573500	OR6C76	4.3297	6.0286	4.2438	6.2422	5.3654	5.2924	5.4673	6.0108	3.5397	5.8221	4.3027	4.9281	3.1608	6.6015	4.0077	5.4203	4.9562	3.1926	3.4029	6.5106	3.8052	5.5801	4.8849	3.9476
CUST_38_PI416379584	OR6C76	1.5271	2.1482	1.7469	2.3711	0.9479	1.8560	1.8021	2.3720	1.4771	2.9380	2.9817	1.5638	2.1003	1.7502	1.4169	2.0865	2.5992	2.8277	1.6746	3.4803	2.8864	1.9282	1.8527	3.2874
**CUST_268_PI416573500**	**OR6F1**	**1.8861**	**2.2848**	**2.0607**	**1.3532**	**2.1508**	**2.0888**	**2.5681**	**1.7631**	**2.1742**	**0.7688**	**1.7754**	**2.0810**	**1.7946**	**2.0711**	**2.3233**	**1.3452**	**1.2192**	**1.8800**	**1.8138**	**2.1771**	**1.7388**	**1.4194**	**1.4245**	**1.6954**
**CUST_276_PI416379584**	**OR6F1**	**2.3601**	**1.9656**	**2.0441**	**1.0564**	**0.7535**	**1.9073**	**2.1533**	**2.1487**	**1.4977**	**1.1029**	**1.4425**	**1.6091**	**1.6815**	**1.7378**	**1.4945**	**1.1901**	**0.8542**	**1.5462**	**1.7019**	**1.7242**	**1.3790**	**0.9911**	**1.0196**	**1.5567**
A_23_P51761	OR6K2	3.7521	3.1015	4.4219	2.7968	5.4756	1.9316	3.6195	3.4613	4.2688	4.9818	3.7786	1.7422	2.9449	3.5418	2.9577	3.6685	4.9755	1.9714	3.6693	1.9819	3.3420	2.7418	5.0741	2.6562
CUST_2234_PI416261804	OR6K2	3.1895	2.4660	4.0393	3.2523	5.1037	2.1618	3.5890	4.2191	4.1046	3.4721	4.2434	2.0070	1.9351	2.9123	3.7928	3.1352	2.4408	2.1158	3.2578	3.2641	3.8006	1.6401	4.4396	2.0460
CUST_382_PI416573500	OR6K3	1.7057	1.8606	1.4386	1.0211	1.1877	1.9708	1.9121	1.4976	1.8007	0.7432	1.2566	1.6258	1.7176	6.3446	1.6068	1.0892	0.8358	1.5439	1.6130	1.6661	1.9630	1.0566	1.6541	1.5361
CUST_391_PI416379584	OR6K3	1.6050	1.8930	1.4307	1.0487	0.7390	1.7961	1.6274	1.5483	1.4395	0.7432	1.1740	1.5928	1.5060	1.7247	1.5528	0.9811	0.6745	1.5503	1.5421	1.9367	1.8730	1.7426	0.8915	1.4321
CUST_397_PI416573500	OR6K6	1.8829	2.6993	1.4570	3.4585	3.3242	2.5627	1.9519	1.8961	1.5889	3.7668	0.9913	3.1280	1.7220	2.1492	2.6872	0.7432	1.5108	1.6007	1.7962	2.0576	2.1027	4.3869	2.4827	2.3501
CUST_406_PI416379584	OR6K6	2.0555	2.7908	2.4169	0.9626	1.4235	1.6691	2.4253	2.1444	2.5096	1.8339	2.1120	1.7797	1.8397	4.0265	1.6235	2.7888	1.2852	1.6083	1.8884	2.9314	3.3756	2.1925	1.3008	2.0131
A_23_P150428	OR6M1	5.5169	4.0185	5.2129	4.2519	5.6946	3.7111	4.8320	5.0380	5.0815	5.0676	4.7515	3.0620	4.3412	5.2312	5.3601	4.1980	4.6456	3.6949	4.5429	2.8513	4.5163	4.0523	5.0540	4.1608
CUST_2003_PI416261804	OR6M1	2.0694	2.1971	2.3805	1.0728	2.8376	1.7683	2.3490	1.8760	1.7792	0.7432	2.8013	1.8489	1.8858	1.9147	2.4034	1.2847	3.5866	1.7122	1.9041	2.0204	2.6240	1.0477	2.9153	1.9519

A_23_P23502	OR6N1	2.3183	2.2245	2.6640	2.8707	4.2373	2.0630	2.8430	1.9109	2.4603	0.7688	2.8908	1.9546	2.0177	1.9898	1.9758	1.2567	2.9940	1.8372	2.1124	2.0531	2.2801	1.2656	3.6500	2.1514
CUST_820_PI416261804	OR6N1	1.9978	2.1772	1.7465	1.3352	1.1882	2.0313	2.1879	1.8107	1.6618	0.7432	1.6266	1.9494	1.8579	2.1138	1.6670	1.5754	1.1326	1.7235	1.8659	1.9874	1.6001	1.4609	1.1980	1.7310
CUST_303_PI416573500	OR6N2	2.6503	2.1624	3.5323	1.7770	4.6789	1.8894	2.9671	3.4939	2.9778	3.0021	3.2629	1.7350	2.0225	1.7817	2.8582	1.2547	2.0085	1.7030	2.6253	1.7393	3.2552	1.8833	3.7513	2.0531
CUST_311_PI416379584	OR6N2	1.9003	1.9147	1.7687	0.9002	0.9982	1.4957	2.2388	2.1041	1.3752	0.7432	1.2874	1.4846	1.6801	1.6131	1.4606	1.1654	0.8364	2.1083	1.8939	1.7201	2.1841	0.7688	0.8601	1.6799
CUST_151_PI416573500	OR6Q1	2.0291	2.1760	3.8398	2.1041	4.2231	2.1981	2.1439	2.7192	3.4135	3.8102	3.3342	2.0192	1.8752	2.1774	3.1453	1.4980	2.7547	1.8404	2.0458	1.9427	3.2894	2.2922	4.3288	1.6490
CUST_156_PI416379584	OR6Q1	1.8681	1.6481	2.1248	0.8271	3.1881	1.4391	2.2573	1.3948	1.9026	0.7432	1.0983	1.4164	1.7088	1.4793	1.4369	0.9601	0.7279	3.9380	2.0167	1.4883	3.2525	0.7432	1.3344	2.5359
CUST_298_PI416573500	OR6S1	1.7748	2.2261	1.7325	1.1970	2.5775	1.9240	2.3763	1.7360	1.9846	0.7432	1.4793	1.9236	2.1500	1.9784	1.7617	1.1949	1.5804	1.7569	1.7752	1.9662	1.5556	1.2229	2.7013	1.5906
CUST_306_PI416379584	OR6S1	1.7238	1.7520	1.3822	0.8781	0.7294	1.5166	2.0151	1.4903	1.8625	0.7432	1.1591	1.4604	1.6197	1.5549	1.4555	0.9889	0.7147	1.8670	1.5620	1.5694	1.9314	0.7688	0.8668	1.5465
CUST_179_PI416573500	OR6T1	1.7482	2.0704	3.0744	2.3950	3.4224	1.8688	1.8501	2.9118	3.7431	1.3365	1.9278	1.8718	1.6734	1.9321	2.7137	1.2397	2.7051	1.6376	1.6808	1.9467	3.4379	1.2318	3.6772	1.5322
CUST_184_PI416379584	OR6T1	1.7727	2.0396	1.6759	1.3726	1.2147	2.0027	2.3095	1.6396	1.5678	0.7432	1.5170	1.8674	1.7243	2.0087	1.6858	1.4748	0.9764	1.6287	2.7183	1.8775	1.5486	1.3704	1.0337	1.4721
CUST_874_PI416573500	OR6V1	2.3584	2.0787	2.2977	2.3435	4.0408	1.7965	1.9952	2.8549	2.9029	1.8423	2.0465	1.7865	1.6862	1.8909	2.2966	2.4330	2.5771	1.5909	1.8802	1.9373	2.8410	1.1884	2.3454	2.4882
CUST_903_PI416379584	OR6V1	2.0488	2.1139	1.9504	1.2493	2.5150	1.8741	2.2804	1.6832	1.6041	1.8543	2.2552	1.7673	1.8303	1.9336	1.6776	1.4428	0.9872	2.3240	1.8794	1.9239	2.1994	1.2329	2.6782	1.7423
A_23_P123164	OR6W1P	6.1003	3.0091	5.2955	4.1259	5.7726	4.9220	5.7066	5.1455	5.3080	4.5970	5.0564	2.7674	4.6236	4.8069	5.3788	3.8194	5.2780	3.5976	5.7501	3.8846	5.3293	4.9727	5.5192	4.1490
CUST_7136_PI416261804	OR6W1P	1.9129	1.9414	2.9505	1.2665	2.8767	1.9786	2.0782	1.4919	2.6244	0.7432	1.9567	1.8156	1.7935	1.9124	2.2028	1.2474	0.6745	1.6850	1.8334	1.7796	1.5717	1.7255	3.6571	1.6191
CUST_282_PI416573500	OR6X1	1.8088	2.2126	3.4435	2.2214	3.0489	1.8933	1.9711	3.4541	3.0864	3.5747	1.3166	1.8343	1.7304	1.8972	3.4017	1.1809	2.5270	1.9759	1.7619	1.9714	3.1191	3.1835	2.6247	1.6846
CUST_290_PI416379584	OR6X1	2.3398	3.7694	2.5716	0.9699	0.9739	1.5720	2.8127	4.2247	2.6303	2.5584	1.6128	3.7499	1.8502	2.9170	1.3693	1.5317	1.2086	1.4719	1.9671	3.2209	3.8366	0.9369	1.0060	3.6857
A_23_P85963	OR6Y1	2.5020	1.8990	2.3949	2.4695	4.0364	1.8186	2.1129	3.6347	3.0149	3.8219	1.9464	1.6193	1.7225	2.8825	1.9537	0.9897	2.8713	1.6018	1.8941	1.7048	2.2005	3.1282	2.2554	1.6043
CUST_2075_PI416261804	OR6Y1	1.7251	2.0977	1.6265	1.1732	1.1343	1.8845	1.8119	1.6656	1.7569	0.7432	1.5178	1.8833	1.6986	1.9578	1.7383	1.2632	0.9849	1.6606	1.6764	2.0001	1.7756	1.2015	1.8835	1.5600
A_24_P357535	OR7A10	1.7930	2.0204	1.8544	1.2288	2.4636	2.2915	1.9123	1.6669	1.5337	0.7432	1.4388	1.8552	2.1975	1.9754	2.0000	1.1766	0.9213	1.7672	1.6985	1.8165	1.5095	1.3054	1.8731	1.6414
CUST_1751_PI416261804	OR7A10	3.0055	1.9689	3.5278	1.1254	3.5228	1.7704	3.8113	1.5518	3.4077	0.7432	3.3728	1.6844	1.8635	1.8293	2.4139	1.2697	3.5287	1.6304	2.2746	1.7998	3.4943	1.1735	4.6216	1.8188
A_23_P28023	OR7A17	2.1034	2.1599	2.8466	1.5158	2.9096	2.2736	2.2351	1.7271	3.1055	2.5927	2.7815	1.9804	1.9642	2.1837	2.2181	1.5917	1.0254	1.8320	1.9673	1.9280	2.2442	1.6742	2.5271	1.7678
A_23_P16630	OR7A5	1.9068	2.1784	1.5886	1.1885	1.6177	2.0422	2.0416	1.6942	1.6987	0.7432	3.2419	1.8444	1.7704	1.9893	1.5741	1.1529	1.8655	1.7771	1.8054	2.0471	1.5215	1.3565	2.4225	2.1344
CUST_2370_PI416261804	OR7A5	1.9217	1.7225	1.8187	2.0947	2.6111	1.8074	2.1344	1.5625	3.0641	0.7432	0.9564	1.5676	1.7978	1.6654	1.9206	1.6122	0.7783	1.6590	1.8170	1.5093	3.0397	1.2031	1.1534	1.7252

CUST_113_PI416573500	OR7C1	1.7301	2.5983	1.5509	1.2321	0.9600	2.5663	1.7908	2.6740	1.6986	0.7432	1.4476	2.6502	1.6156	1.9567	1.6379	1.2492	0.9504	1.9988	1.6583	1.9237	2.1758	1.3256	1.4638	1.5037
CUST_117_PI416379584	OR7C1	1.8657	2.0130	1.6048	1.2645	1.1518	1.9327	2.0496	1.6262	1.5023	0.7432	1.4664	1.7669	1.7277	1.9212	1.5947	1.4265	0.9653	1.5820	1.7637	1.8588	1.5042	1.2897	1.0142	1.4956
A_23_P108265	OR7C2	2.3993	2.2516	2.9551	1.2669	4.3348	2.0402	2.6101	2.8559	3.1152	2.4142	3.0732	1.9095	2.3025	1.9633	2.4145	2.4059	2.3342	1.8299	2.1367	2.0828	2.5494	1.3798	4.5615	1.9757
CUST_1059_PI416261804	OR7C2	1.7872	2.2873	1.6824	1.5330	1.2217	2.7202	1.8078	3.1902	2.0297	1.4824	1.6375	1.9899	1.6605	2.1274	1.7433	2.2012	1.2945	1.9184	1.6782	2.0419	2.3353	1.9306	1.8638	2.2218
A_23_P410408	OR7D2	1.6603	1.9544	1.4582	1.0700	1.3703	1.8289	1.7540	1.5404	1.8032	0.7432	1.1933	1.6142	1.5638	1.7228	1.5463	1.0942	0.7173	1.4712	1.6077	1.7540	1.4015	1.1302	0.9170	1.4467
CUST_1031_PI416261804	OR7D2	1.8898	2.1490	1.6488	0.9609	0.8706	1.6817	2.1229	1.6103	1.5532	0.7432	1.6640	1.5838	1.6476	1.6906	2.0094	1.1082	0.8630	1.4547	1.7251	1.8270	2.2761	0.9747	1.0067	1.5990
A_23_P130547	OR7D4	2.0498	1.9539	2.1599	1.0877	2.2569	1.7739	2.3434	1.5904	1.6588	0.7432	1.7493	1.6491	1.7691	1.7776	1.8918	1.2080	0.7166	1.5858	2.2956	4.1310	2.6718	1.1415	2.6951	1.7223
CUST_6531_PI416261804	OR7D4	2.0072	2.1275	1.6375	1.1617	2.1334	1.8205	2.2065	1.6158	1.8760	0.7432	1.6049	1.7902	1.8170	1.8556	1.6146	1.3098	0.9575	1.5689	1.8854	1.8306	1.5454	1.1674	1.8393	1.6746
A_24_P384018	OR7E156P	5.7400	5.9271	6.5679	6.7950	6.5084	5.6726	6.2831	5.7441	6.2838	6.4531	6.0732	5.7277	6.1975	5.2063	6.6759	5.7731	5.8723	5.6738	6.7090	6.0965	6.3473	6.9341	6.3415	5.6516
A_32_P32391	OR7E156P	6.6077	5.9516	6.3955	6.9488	6.7984	6.1202	6.9087	5.7035	6.2756	6.7532	6.4249	5.4979	6.3072	5.2973	6.4317	5.7429	5.7464	5.7122	6.7634	6.3178	6.8083	6.8768	6.5231	6.6627
A_23_P16110	OR7E24	5.9905	5.3210	6.3571	6.4132	6.7403	5.6703	6.3397	5.5408	6.1132	6.1744	6.0864	5.0488	6.2271	4.8133	6.3211	5.4875	5.5251	5.2661	6.3875	5.4288	6.2885	6.5072	6.3373	5.6042
CUST_1091_PI416261804	OR7E24	2.1967	2.0204	1.8166	1.0770	1.7964	1.6700	2.0436	2.2446	1.8864	0.7432	1.5137	1.6975	1.7264	1.7979	1.8562	1.2147	1.0633	1.5363	1.7267	1.8993	1.5891	1.0529	1.1400	1.6198
CUST_7089_PI416261804	OR7E36P	7.5332	5.9325	6.9958	7.1068	7.0255	6.4193	6.9187	6.0146	6.7352	7.0171	6.5852	5.7676	6.8643	5.8895	6.9889	6.3537	6.3065	6.4158	7.4280	6.4673	7.0203	7.3861	6.7825	6.5952
A_23_P408930	OR7E5P	3.9919	2.7195	4.5750	4.7061	3.7692	2.9104	4.4548	2.9227	4.5281	5.1208	2.8015	2.1988	3.7077	2.3458	4.3326	4.3118	3.5401	2.6532	4.5818	3.1150	4.0724	3.9373	2.8032	3.1775
A_23_P433218	OR7E91P	6.3591	6.8110	6.0276	6.4728	6.0630	5.4326	6.8862	6.3122	5.9274	6.4964	5.1968	4.1610	6.2662	6.3500	5.7453	5.8467	5.5580	5.8282	7.0174	6.3322	5.9218	6.2565	5.7065	6.4184
A_24_P332718	OR7E91P	2.2537	2.3893	2.7264	1.7713	2.8776	2.5572	2.3044	1.7066	3.0870	3.5869	1.7032	2.1600	1.9764	2.2665	2.4295	3.0455	3.5367	2.0940	2.0692	2.1273	3.0525	1.9599	2.5490	1.9234
CUST_248_PI416379584	OR7G1	3.9726	3.3759	3.9527	3.5511	3.5907	3.8383	3.8466	4.0437	3.7672	4.1135	2.6432	2.4443	4.2833	3.7920	4.0254	3.4786	3.2281	3.8615	3.3649	2.1581	3.1863	4.4143	2.8806	4.0900
CUST_335_PI416573500	OR7G2	1.6555	1.9325	1.5598	1.1499	1.9587	1.7811	1.6961	1.5645	1.8043	0.7432	1.4123	1.6835	1.6100	1.8384	1.6866	4.4351	0.9059	1.4667	1.5649	1.7508	1.4385	1.1271	1.0312	1.4121
CUST_344_PI416379584	OR7G2	2.2807	2.4055	3.2748	2.4170	4.0865	1.9420	2.5085	1.7796	4.0252	3.6518	2.0217	2.0011	1.8836	2.0358	3.2092	1.3542	3.8978	1.7591	2.3067	2.2059	3.6853	1.3218	2.8640	1.9950
A_23_P141939	OR7G3	1.7634	2.1556	1.7578	1.3026	1.6484	1.9621	1.8616	1.8664	2.1344	0.7432	1.6548	1.9396	1.7113	2.0741	1.7545	1.3882	1.0688	1.7054	1.6582	1.9754	2.1409	1.3185	1.5994	1.5482
CUST_1434_PI416261804	OR7G3	2.2514	2.2448	2.2851	1.1141	0.9938	1.8874	2.5965	2.3253	1.5124	2.7266	1.6999	2.4015	1.8318	2.6379	2.1816	1.5173	1.0587	2.5091	2.5451	2.0120	2.6556	1.5675	1.1828	2.9958
CUST_404_PI416573500	OR8A1	1.7624	2.1108	1.6389	1.2326	0.9708	1.8964	1.8949	1.8074	1.6209	0.7432	1.6214	1.9139	1.7463	2.0392	1.7703	1.3320	1.1500	1.7205	1.6526	1.9323	1.5853	1.2575	1.1515	2.0501

CUST_413_PI416379584	OR8A1	1.6658	1.7930	1.8821	0.9586	1.9348	1.6617	1.7996	1.3430	2.1086	0.7432	1.0365	1.5216	1.6296	1.6312	1.7092	0.9266	0.6745	1.5095	1.6081	2.1918	1.4410	1.9193	0.9130	2.9904
CUST_241_PI416573500	OR8B12	2.9706	2.3472	3.5082	2.5789	5.3273	2.2696	3.2462	4.5451	4.7163	4.3576	3.9125	1.8899	3.2023	4.5611	4.6961	4.1084	4.5194	1.9831	2.7325	2.9424	3.8805	3.8011	4.6270	2.3716
CUST_249_PI416379584	OR8B12	2.3343	2.0859	2.2395	1.2283	1.1756	2.2239	2.6334	2.2908	1.5951	0.7432	1.5398	1.7363	1.9914	2.4382	1.5204	1.4851	1.6394	1.6807	2.1178	1.7979	2.5615	1.2633	1.1693	1.9614
CUST_333_PI416573500	OR8B2	1.6904	1.6993	1.9810	0.8761	2.6575	1.5336	1.9554	1.3996	2.0657	1.3793	1.5427	1.4669	1.6556	1.5722	1.8546	0.9690	1.5774	1.4257	1.5585	1.5192	2.2973	0.8919	2.4404	1.4907
CUST_450_PI416379584	OR8B2	2.6435	3.2443	1.7792	1.3125	1.1318	2.5751	2.3674	2.1109	1.7754	1.4242	1.6004	1.8490	1.7892	2.5881	1.5340	1.5278	0.9632	2.4136	2.0001	3.6373	1.5688	1.3843	1.2006	2.4685
CUST_342_PI416379584	OR8B3	3.0624	1.9781	2.8260	1.3672	4.7468	2.0303	2.9481	3.5464	3.1404	1.4500	3.5229	1.7046	2.2068	1.8813	3.5985	2.4412	1.5339	1.6690	2.1420	2.3720	3.0048	1.3702	3.8171	1.7951
CUST_336_PI416573500	OR8B4	1.8271	2.3492	1.8744	1.3655	1.2865	2.0828	1.8708	1.7984	1.7160	0.7432	1.8315	2.1437	1.7715	2.0880	1.9062	1.3958	1.3217	1.8620	1.7704	2.2325	1.6474	1.3955	1.3998	1.6676
CUST_345_PI416379584	OR8B4	2.2318	2.0150	1.4517	1.0916	0.7138	1.7305	1.8801	2.1098	1.4615	0.7432	1.3548	1.6953	1.6656	1.7787	1.5545	1.1023	0.8701	1.6994	1.6024	1.7272	1.4401	1.0288	0.9847	1.4754
A_23_P1819	OR8B8	3.4319	2.1282	3.8309	4.3411	4.6489	3.3316	3.5966	3.2559	4.0320	4.9351	4.0529	1.7425	2.6949	4.0435	4.1154	3.5596	3.0694	2.1335	2.7640	1.6966	3.7214	5.3053	3.8556	3.1300
CUST_1581_PI416261804	OR8B8	2.1776	3.9976	1.7512	1.5122	1.0670	3.7718	2.3200	2.8665	1.8608	1.1762	1.6794	2.0411	1.8659	2.6144	1.7772	1.5713	0.9341	2.8983	3.0896	3.4541	2.2530	2.5437	1.4728	2.6555
A_23_P127662	OR8D1	1.8623	2.4179	2.3565	1.2811	3.1304	2.1278	1.9423	2.8606	2.5059	0.8910	2.8648	2.0788	1.7846	2.1789	1.9076	1.3502	3.7287	1.9036	1.8812	2.3412	1.8979	1.3163	3.3406	1.7178
CUST_1503_PI416261804	OR8D1	1.8357	2.1226	1.9954	1.0103	0.7126	2.1032	2.1060	1.6750	1.4757	0.8622	1.2983	1.6192	1.6871	1.8352	1.3764	1.0299	0.7332	3.2527	1.7122	3.6226	2.0054	0.9361	1.2562	1.8444
A_23_P139050	OR8D2	2.1353	2.1349	1.8339	1.0981	2.9912	1.9518	2.4138	1.6487	1.4862	0.7432	1.6364	1.7884	1.9341	1.8125	1.5346	1.0055	0.7492	1.8265	2.6622	1.8627	1.9494	1.1961	2.7496	2.0415
CUST_2150_PI416261804	OR8D2	2.1942	2.0955	2.2232	1.8350	2.1453	3.6052	2.3939	1.6824	2.1053	0.7432	2.2933	1.8708	2.1633	2.1006	1.7032	1.4862	1.0837	1.7986	2.0447	2.0796	2.3963	1.4758	2.1887	1.8947
CUST_444_PI416573500	OR8D4	1.6821	1.9982	1.5911	1.1643	1.5754	1.8913	1.7421	1.5760	1.5778	0.7432	1.3085	1.7119	1.5785	1.8244	1.6250	1.2096	0.7159	1.5155	1.6325	1.8230	1.4462	1.2186	1.1105	1.4579
CUST_453_PI416379584	OR8D4	1.6156	1.9539	1.5806	0.9328	1.9288	1.8512	1.8265	1.5175	1.3686	0.7432	1.1707	1.5387	1.5336	1.6406	1.4976	1.0735	0.6745	1.4176	1.5633	2.5906	1.3430	0.9675	0.8425	1.4530
A_23_P13195	OR8G1	1.9480	2.1577	1.5392	1.1717	0.9085	1.8919	2.0836	1.6457	1.5807	0.7432	1.5877	1.7801	1.7680	1.8621	1.5777	1.1999	1.0658	1.6739	1.8060	1.9849	1.5333	1.2531	1.2692	1.7106
CUST_1487_PI416261804	OR8G1	1.7727	2.2208	1.6367	1.2657	2.2094	2.0174	1.8218	1.7999	1.5904	0.8231	3.1490	1.9245	1.6750	1.9991	1.7468	1.2941	0.9948	1.7049	1.7018	2.0974	1.4611	1.3156	5.4196	1.5772
A_23_P52868	OR8G2	2.1785	2.3448	1.5120	1.5646	1.5637	1.7361	2.4999	1.7301	1.7575	1.2299	1.5808	1.7485	1.7725	3.0997	1.5913	1.2653	1.0332	1.5814	1.9448	2.0398	2.3232	2.3030	2.1950	1.9175
CUST_185_PI416573500	OR8G2	2.2737	2.4680	1.4551	0.9000	0.8778	1.5132	1.9736	1.6257	1.4468	0.7432	2.0205	1.5569	1.6276	1.6493	1.5192	1.1940	1.0432	1.4235	1.6066	1.8513	1.4497	1.5948	0.9860	2.5664
CUST_190_PI416379584	OR8G2	3.1100	2.9094	4.1689	3.4292	5.0484	1.9028	3.4134	2.9982	4.1414	3.5299	3.6112	1.6587	2.1008	2.4858	3.9656	0.9611	2.5899	2.0224	3.2973	2.6532	3.9028	2.3652	4.6613	2.4522
CUST_242_PI416573500	OR8G5	1.6582	1.9597	1.4869	1.1195	0.7345	1.7977	1.7235	1.5797	1.4857	0.7432	1.3611	1.7155	1.6201	1.8101	1.6044	1.1112	0.8191	1.4835	1.5880	1.7146	1.4311	1.1074	0.9954	1.7367
CUST_250_PI416379584	OR8G5	2.6503	2.3727	2.4766	1.9709	0.8129	1.5841	2.3588	2.3133	1.7899	0.7432	1.7517	1.7275	1.6660	1.7815	1.4820	1.2444	0.7920	1.5834	1.8001	3.7568	2.8575	1.5718	1.1736	2.5071
A_23_P75707	OR8H1	1.9512	1.9068	2.7791	1.9984	3.8147	1.8744	2.1672	1.5444	2.8617	2.7255	2.4097	1.7381	1.8454	2.1129	3.3039	1.0534	0.8379	1.7465	1.7999	1.6878	3.0657	1.2502	2.8437	1.7576

CUST_1761_PI416261804	OR8H1	2.3348	2.0316	2.9973	1.3044	2.2914	2.1735	2.6111	1.6355	2.2191	2.2970	1.3752	1.8764	2.0291	2.0316	1.6936	1.2890	2.8473	1.9037	2.2837	1.8181	3.1591	1.6334	2.6138	2.0260
CUST_457_PI416573500	OR8H2	2.3674	2.1932	3.8327	2.6408	4.0966	2.3242	2.5993	2.7311	3.8943	3.5926	3.1965	1.9874	2.1144	2.2074	3.8195	2.6348	3.5023	1.9149	2.6296	1.9409	2.9551	1.8202	4.3723	2.9370
CUST_466_PI416379584	OR8H2	1.8858	1.9353	2.6450	1.2082	3.4007	1.9503	2.2110	2.1580	2.7484	0.9334	5.3362	1.7516	1.7212	1.8409	2.2436	1.1889	2.6699	1.6089	1.7803	1.7585	1.5169	2.3878	3.3704	1.5648
CUST_243_PI416573500	OR8H3	1.6734	2.1170	1.7195	1.1593	0.8167	1.8348	1.7295	1.7052	1.5473	0.7432	1.4919	1.8602	1.6857	1.9357	1.7317	1.1970	1.0391	1.6547	1.6311	2.0089	1.4817	1.1586	1.0642	1.5242
CUST_251_PI416379584	OR8H3	2.2143	2.3456	2.2636	1.4451	3.4317	3.4052	2.5496	1.7442	2.6133	1.6000	1.2102	1.6402	1.8441	2.4239	2.0043	1.1197	2.4553	1.6025	2.0740	1.7605	2.3835	1.7388	3.1849	1.8823
CUST_362_PI416573500	OR8I2	1.7143	1.8930	1.4844	0.9668	1.0280	1.5911	1.9241	1.5308	1.4784	0.7432	1.3796	1.6172	1.6702	1.6728	2.1306	1.1331	0.9431	1.4725	1.6313	1.7898	1.4903	0.9523	1.0013	1.5605
CUST_371_PI416379584	OR8I2	1.7902	2.0363	1.4471	1.0565	0.8519	1.8600	1.9857	1.4969	1.4158	0.7432	1.1667	1.5878	1.6252	1.7056	1.4378	1.2579	0.6745	1.4826	1.6738	2.8711	1.8619	2.0446	0.9163	3.3486
A_23_P13244	OR8J1	2.6702	2.1146	2.4796	1.2184	1.1916	1.9222	3.1199	1.7275	2.6923	1.1617	1.9263	1.8080	2.3677	1.9942	2.4317	1.5293	1.0844	2.3059	2.9078	1.9266	2.3710	1.3004	1.7853	3.3645
CUST_1234_PI416261804	OR8J1	1.9114	2.0258	2.9380	1.1639	1.9461	1.7670	2.1296	1.6224	3.5133	2.6473	1.4779	1.7166	1.7451	1.8803	2.4607	1.2140	0.9137	1.5408	1.7668	1.8659	3.6480	1.1392	2.2795	1.6201
CUST_369_PI416573500	OR8J3	1.7952	1.9173	1.5503	1.1888	1.7705	1.8946	1.9370	1.4951	1.5336	0.7432	1.3505	1.7118	1.9294	1.8293	2.1418	1.1304	2.3426	1.5648	1.6803	1.6710	2.1134	1.2193	1.1338	2.3612
CUST_378_PI416379584	OR8J3	1.8286	2.5683	1.6482	1.3216	1.2164	2.8363	1.8676	1.8814	1.6316	0.7688	1.7833	2.0954	1.6831	2.8985	1.7783	1.3447	1.0837	1.7441	1.7142	3.4545	1.5744	3.2491	1.2602	1.5928
CUST_347_PI416573500	OR8K1	1.7131	1.8276	1.4162	1.0020	0.8231	1.6806	1.9311	1.4426	1.4563	0.7432	1.1620	1.5707	1.6677	1.6802	2.1215	1.0652	0.7160	1.5110	1.6317	1.6439	1.4321	1.0620	0.9031	2.4586
CUST_356_PI416379584	OR8K1	2.0755	2.2578	1.7460	2.0747	0.9348	2.8840	2.0956	1.5625	1.4781	8.2915	1.2577	2.1648	1.5042	1.7654	1.5095	1.0813	0.6987	2.8463	1.5383	3.6009	1.9859	2.8614	0.9101	1.7670
A_23_P36050	OR8K3	1.9688	2.3307	1.6214	1.1664	0.8478	2.1524	2.0066	2.0416	1.6585	0.7432	1.5159	2.0030	1.8127	1.9715	1.6027	1.2752	0.8226	1.6885	1.8183	2.1735	1.6011	1.2178	1.8383	1.8472
CUST_855_PI416261804	OR8K3	1.7580	2.1069	1.5734	1.2211	0.8846	1.9066	2.1766	1.7524	2.8014	0.7432	1.6617	1.7061	1.6375	1.9135	1.6331	1.2130	0.8333	1.6414	1.6244	1.9031	2.0764	1.2521	1.4801	1.4828
CUST_138_PI416573500	OR8K5	1.9333	2.3560	1.6666	3.2638	0.9279	2.0477	2.1789	1.6746	1.6151	0.7432	1.6723	1.9420	1.7861	1.9892	1.7538	1.3105	0.8937	2.5891	1.7867	1.9941	1.6440	1.3232	1.2907	1.7242
CUST_143_PI416379584	OR8K5	2.0351	2.2731	1.6051	1.2358	0.8840	2.0004	2.1759	2.5692	1.5826	0.7432	1.6394	1.8108	1.7567	1.9128	1.5678	1.3020	0.8107	1.5693	1.8756	1.9257	2.0961	1.2688	1.2214	1.6689
CUST_198_PI416573500	OR8S1	3.7575	1.9229	2.9964	1.1548	4.6058	2.3798	3.8838	3.8849	3.6503	2.1916	2.4355	3.3154	2.5109	3.4300	1.7103	2.4730	0.6841	2.4988	4.1058	1.8538	3.6428	1.2512	3.9541	4.2638
CUST_203_PI416379584	OR8S1	2.7996	3.4726	2.9499	3.4471	2.5178	3.4451	3.1997	3.2815	2.9903	3.5171	3.8355	3.5372	1.7750	1.8286	2.6540	2.0013	2.4468	2.5217	2.2799	2.6528	2.4689	2.4505	2.6806	3.3869
A_23_P24676	OR8U1	1.7773	1.7083	2.4066	0.9752	3.7371	1.7038	1.9416	3.0502	2.0269	1.9076	2.3101	1.5188	1.7007	1.6041	1.6554	0.9711	0.6745	1.5509	2.1785	1.5273	1.9050	1.0913	2.3532	1.5560
CUST_147_PI416573500	OR8U1	1.7830	2.0594	1.5977	1.3158	1.0531	2.0662	1.8741	1.6264	1.4977	0.7432	1.4163	1.8335	1.6747	1.9688	1.5650	1.3236	0.7591	1.6033	1.7229	1.8667	1.4997	1.3727	1.0762	1.4739
CUST_5340_PI416261804	OR8U1	1.9417	1.9567	1.5286	1.1765	0.7728	1.8683	1.7263	1.6318	1.5324	0.7432	1.3876	1.6877	1.5953	1.8445	1.5969	1.1338	0.7606	1.4664	1.5835	1.7362	1.4206	1.1987	1.0364	1.4585
CUST_152_PI416379584	OR8U8	2.2377	2.2002	1.5505	1.1144	0.9437	1.8456	2.5517	1.9985	1.8020	0.7432	2.5279	1.9145	1.8475	1.9229	1.5843	1.3434	1.0499	1.6630	1.9455	2.0393	2.2070	1.0869	1.2487	2.0452

CUST_1408_PI416261804	OR8U9	2.1205	1.8883	2.0880	1.1973	3.8414	1.9104	2.3955	1.7397	3.1659	1.2339	2.4498	1.7160	2.0629	1.8276	1.6337	1.0428	2.7728	1.6725	1.9531	1.6216	2.2775	1.2611	2.5976	1.7748
CUST_467_PI416573500	OR9A2	2.4133	1.8931	1.6317	1.2266	1.0275	2.0851	2.7379	1.4852	1.6794	0.7432	1.1954	1.7980	2.1758	1.9124	1.5709	0.9869	0.7838	2.1200	2.2179	1.6573	1.7072	1.4610	1.6182	2.3364
CUST_477_PI416379584	OR9A2	3.1375	2.9641	3.1017	1.2423	3.3071	2.1290	3.1071	3.6616	3.3306	3.5808	3.8140	1.8320	2.0271	3.0048	1.8661	2.5785	2.8750	1.7224	2.8773	3.2731	3.6224	2.6427	3.5294	1.7446
CUST_600_PI416573500	OR9A4	1.8100	2.2546	1.7720	1.4075	2.6223	2.0174	1.8540	1.7541	1.7280	0.7432	1.7420	1.9408	1.7197	2.0367	1.7788	1.4433	1.3554	1.6245	1.7085	1.9759	1.8748	1.3383	1.6695	1.4937
CUST_616_PI416379584	OR9A4	1.9102	2.2710	1.6231	1.2661	1.1546	2.0092	2.0222	1.8453	1.5845	0.7432	1.7133	1.9026	1.7221	1.9923	2.1536	1.3004	1.0430	1.6952	1.7763	2.1203	1.4807	1.3141	1.3040	1.6798
CUST_1065_PI416379584	OR9G1	2.0022	2.4858	2.2453	2.1463	3.5809	2.6553	2.1916	2.5808	2.9401	1.7867	3.0454	3.4946	1.8855	2.2005	1.9266	2.7918	3.4197	1.8494	1.9400	2.2077	2.4653	2.7296	3.1668	2.1395
CUST_149_PI416573500	OR9G1	1.7304	2.1275	1.7091	1.1787	1.1341	1.9176	1.8129	1.6994	1.6571	0.7432	1.5196	1.8855	1.6924	1.9757	1.7048	1.2578	0.9656	1.6708	1.6773	2.0236	1.7971	1.2207	1.2228	1.5693
CUST_1013_PI416573500	OR9G4	1.5997	1.7873	1.4204	0.9891	0.7299	1.6469	1.6691	1.4067	1.4580	0.7432	1.1345	1.5700	1.5695	1.6692	1.6203	0.9833	1.1021	1.4508	1.7561	1.6142	1.4321	1.0123	0.8884	1.4440
CUST_1045_PI416379584	OR9G4	1.6577	3.4562	1.9602	3.6801	1.3823	2.9874	1.8416	4.1127	1.3573	0.7432	1.2174	2.6833	1.8241	3.3013	1.4903	1.1452	0.7251	2.2055	1.6321	2.7740	1.6576	2.3729	0.7791	2.7057
CUST_154_PI416379584	OR9G9	1.6836	2.1444	1.5038	1.1398	2.4296	1.8948	1.7543	1.7182	1.4936	0.7432	1.4172	1.7740	1.6050	1.8176	1.5515	1.1304	0.7152	1.4955	1.6342	1.8336	1.9785	1.1579	1.0614	1.4730
CUST_415_PI416573500	OR9I1	2.3601	2.2710	2.1125	1.3101	1.1030	2.0608	2.0703	1.7495	1.8167	2.6051	1.6868	1.9521	1.7865	2.8383	2.0449	1.2758	1.0529	1.8068	1.8501	2.1124	1.5929	1.4089	1.3820	1.7264
CUST_424_PI416379584	OR9I1	1.6027	3.0672	1.7062	0.9662	0.6924	1.6195	1.5955	2.5509	1.4392	0.7432	1.6601	1.6370	1.5463	2.5811	2.3494	1.4477	0.9446	1.6052	1.5956	1.7810	2.2916	0.9199	0.8780	3.1778
CUST_452_PI416573500	OR9K2	1.5653	1.8642	1.4595	1.0270	0.7582	1.6436	1.6011	1.4911	1.4878	0.7432	1.2550	1.6323	1.5290	1.7527	1.6731	1.0689	0.8092	1.4377	1.5304	1.6649	1.9178	1.0092	2.6082	1.4201
CUST_461_PI416379584	OR9K2	2.0043	2.2432	1.7364	1.3785	1.0888	2.2704	2.1271	2.1547	1.6164	5.6809	1.6165	2.0134	1.7910	2.1291	1.5876	1.4899	0.9300	1.7227	1.7950	2.0177	1.9788	1.5960	1.2854	1.7254
CUST_270_PI416573500	OR9Q1	2.0726	1.8428	3.2281	0.8162	1.2339	2.1381	2.2649	2.5172	2.3946	0.9206	1.3124	1.4135	1.7995	3.2506	1.3874	1.1113	0.9946	1.4789	1.9921	1.6394	4.4376	0.7432	1.3783	3.3594
CUST_278_PI416379584	OR9Q1	3.0915	2.1760	2.6533	1.2000	2.7659	2.1076	3.1319	3.3307	2.1269	2.6684	1.4565	1.6113	2.2982	2.0817	2.3606	1.3492	2.7262	2.8990	2.4004	3.8469	3.2332	1.2529	3.5821	3.3478
CUST_345_PI416573500	OR9Q2	2.1563	2.0917	2.4079	1.2527	3.0096	1.9812	2.4026	1.8222	2.9468	0.7432	1.6100	1.9488	1.9532	2.0718	2.4935	2.9290	1.0990	1.8286	2.2575	1.9144	2.8141	1.3470	1.6414	1.8953
CUST_354_PI416379584	OR9Q2	3.4175	2.0769	2.4462	1.0638	0.7943	2.2305	2.9296	3.2136	2.2381	0.7432	1.4637	2.4494	1.7903	2.2077	1.4191	1.1999	1.1523	1.8589	2.1933	2.3804	3.4890	0.7432	0.9332	3.5289

Supplementary Table 1 : Expression values of each olfactory receptor in the four amygdala nuclei of six individuals, according to Allen brain atlas. The 376 genes coding for human olfactory receptors (second column) were quantitated, using at least one DNA probe (first column). Number of copies are reported as log2 values. The 20 olfactory receptors selected for the overall area mapping are highlighted in bold while pseudogenes appear in white font on a grey-black background.
