# Supplementary figures and images for: Limbic Expression of mRNA Coding for Chemoreceptors in Human Brain—Lessons from Brain Atlases
Source: Int J Mol Sci. 2021 Jun 25;22(13):6858. doi: 10.3390/ijms22136858 (PMC8267617; doi:10.3390/ijms22136858)

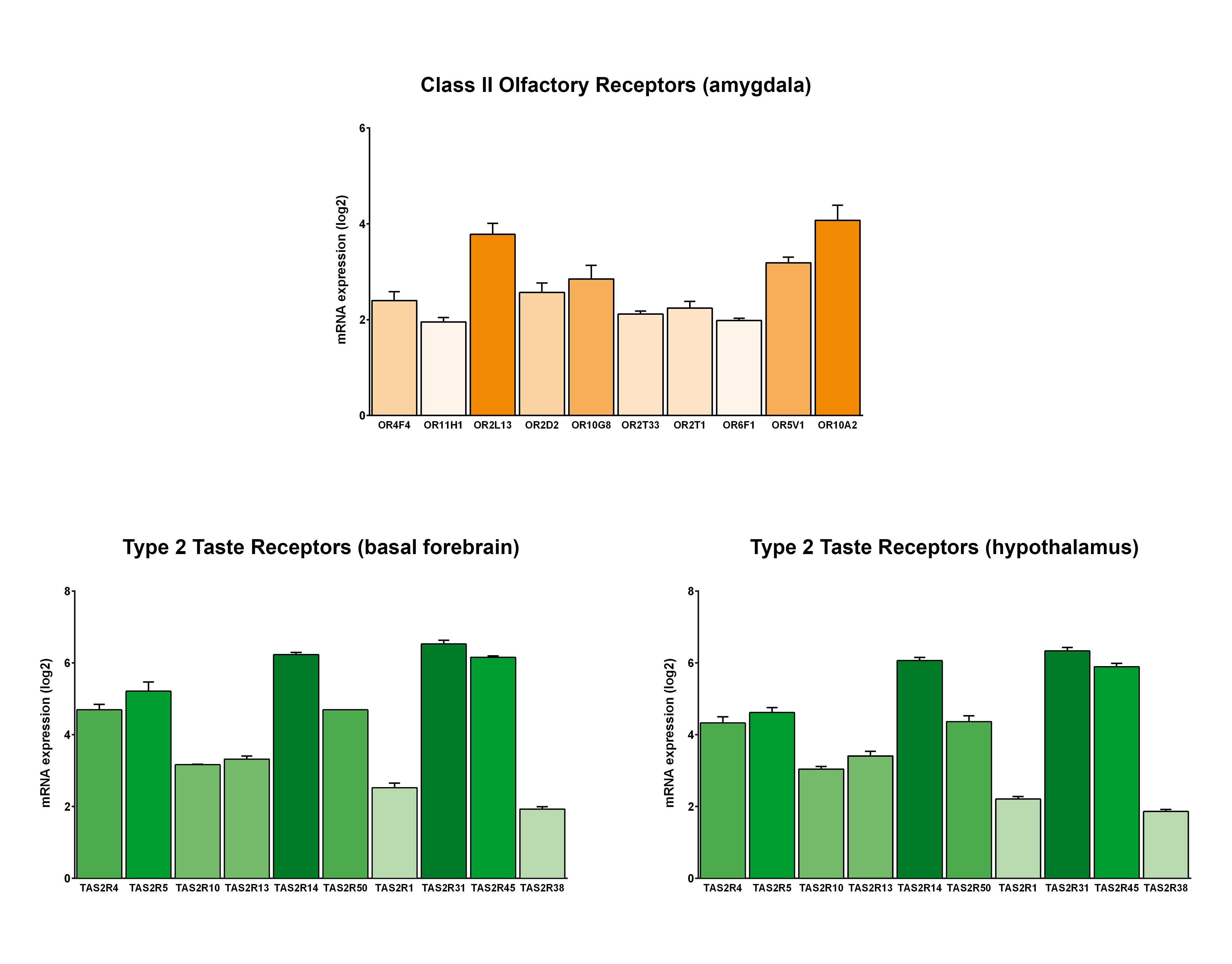

Supplement: Supplementary file 1 [file ijms-22-06858-s001.zip › FigS2.tif]

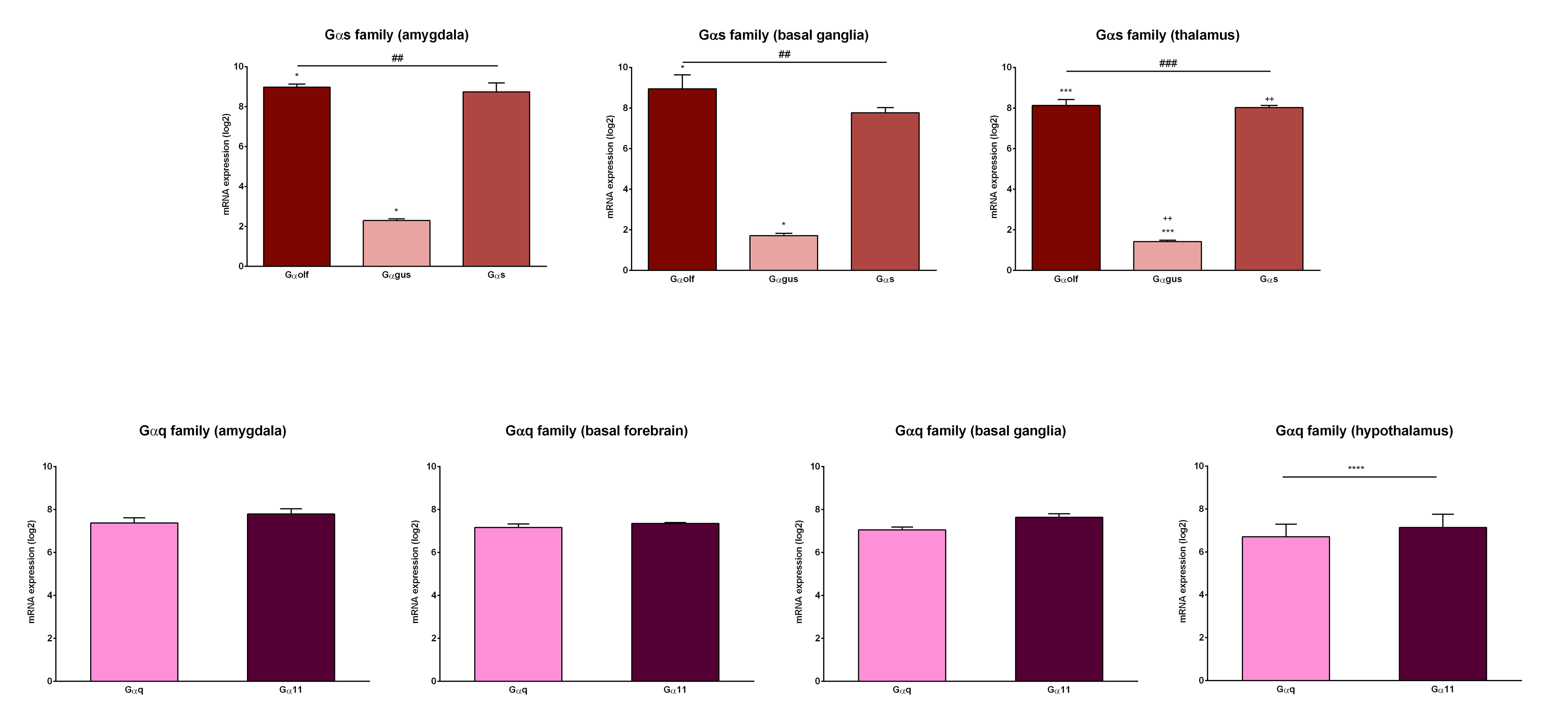

Supplement: Supplementary file 1 [file ijms-22-06858-s001.zip › FigS3.tif]
